# Comparative proteomics as a tool for identifying specific alterations within interferon response pathways in human glioblastoma multiforme cells

**DOI:** 10.18632/oncotarget.22751

**Published:** 2017-11-29

**Authors:** Irina A Tarasova, Alesya V Tereshkova, Anna A Lobas, Elizaveta M Solovyeva, Alena S Sidorenko, Vladimir Gorshkov, Frank Kjeldsen, Julia A Bubis, Mark V Ivanov, Irina Y Ilina, Sergei A Moshkovskii, Peter M Chumakov, Mikhail V Gorshkov

**Affiliations:** ^1^ Talrose Institute for Energy Problems of Chemical Physics, Russian Academy of Sciences, 119334 Moscow, Russia; ^2^ Engelhardt Institute of Molecular Biology, Russian Academy of Sciences, 119991 Moscow, Russia; ^3^ Chumakov Institute of Poliomyelitis and Viral Encephalitides, Russian Academy of Sciences, 142782 Moscow, Russia; ^4^ Moscow Institute of Physics and Technology, 141701 Dolgoprudny, Russia; ^5^ Department of Biochemistry and Molecular Biology, University of Southern Denmark, 5230 Odense M, Denmark; ^6^ Orekhovich Institute of Biomedical Chemistry, 119121 Moscow, Russia; ^7^ Pirogov Russian National Research Medical University, 117997 Moscow, Russia

**Keywords:** comparative proteomics, glioblastoma multiforme, oncolytic virotherapy, interferon signaling alterations, JAK/STAT pathway

## Abstract

An acquisition of increased sensitivity of cancer cells to viruses is a common outcome of malignant progression that justifies the development of oncolytic viruses as anticancer therapeutics. Studying molecular changes that underlie the sensitivity to viruses would help to identify cases where oncolytic virus therapy would be most effective. We quantified changes in protein abundances in two glioblastoma multiforme (GBM) cell lines that differ in the ability to induce resistance to vesicular stomatitis virus (VSV) infection in response to type I interferon (IFN) treatment. In IFN-treated samples we observed an up-regulation of protein products of some IFN-regulated genes (IRGs). In total, the proteome analysis revealed up to 20% more proteins encoded by IRGs in the glioblastoma cell line, which develops resistance to VSV infection after pre-treatment with IFN. In both cell lines protein-protein interaction and signaling pathway analyses have revealed a significant stimulation of processes related to type I IFN signaling and defense responses to viruses. However, we observed a deficiency in STAT2 protein in the VSV-sensitive cell line that suggests a de-regulation of the JAK/STAT/IRF9 signaling. The study has shown that the up-regulation of IRG proteins induced by the IFNα treatment of GBM cells can be detected at the proteome level. Similar analyses could be applied for revealing functional alterations within the antiviral mechanisms in glioblastoma samples, accompanying by acquisition of sensitivity to oncolytic viruses. The approach can be useful for discovering the biomarkers that predict a potential sensitivity of individual glioblastoma tumors to oncolytic virus therapy.

## INTRODUCTION

Glioblastoma multiforme (GBM) is highly aggressive still incurable malignant brain tumor [1, 2]. Challenges in glioblastoma treatment include a problematic complete surgical removal of the tumor, a deep penetration of GBM stem cells into normal brain tissue [3, 4], a fast proliferation of malignant cells, and a high resistance of glioblastoma stem cells to radiation [5] or chemotherapy [6] that drive tumor recurrence [7]. The notorious inter- and intra-tumoral heterogeneity of GBM cells [8] explains eventual failures of existing targeted and immune therapy approaches [9, 10].

Currently, replication competent oncolytic viruses are being developed and tested as promising approach to therapy of GBMs [11–16]. Cancer cells commonly acquire increased sensitivity to replication and killing by viruses of many families. Oncolytic viruses are non-pathogenic naturally occurring or engineered strains of viruses that can selectively infect and kill cancer cells while sparing cells of normal tissues [17–20]. Besides the direct virus replication-dependent cell lysis, the infection initiates complex changes in tumor microenvironment and activation of both innate and adaptive branches of anticancer immunity that contribute to a long-term therapeutic effect even after the virus is cleared from tumor sites [21–25]. Another important advantage of oncolytic viruses is their ability to kill the cancer-initiating stem cells, thus, limiting the probability of relapses [26–29]. The activity against GBM stem cells has been demonstrated for many oncolytic viruses [30–35], and some long-term remissions following GBM virotherapy in limited clinical trials [36–39] suggest that viruses could provide a long lasting cure for the patients.

During malignant progression, the cancer cells accumulate multiple molecular alterations enabling the accelerated growth and invasion. However, the loss of important control mechanisms comes at the expense of the increased sensitivity to viruses [40]. Cancer cells are more accessible to viruses because of the disorganized architecture of tumors, overexpression or overexposure of the surface virus entry receptors and leaky neovascular network [41]. Besides, cancer cells generally have relaxed metabolic regulation, disrupted cell-cycle control, and lost suicidal reaction in response to stresses and pathogens [42, 43] that provide additional benefits for a robust replication of viruses. Among the main targets for inactivation of antiviral mechanisms during cancer progression are multiple components of antiviral innate immunity, including the IFN induction and IFN response mechanisms [40, 44–49]. Inactivation of these mechanisms by mutations or epigenetic silencing renders cancer cells sensitive to selective killing by oncolytic viruses. In previous studies, a number of important examples demonstrating a variety of responses of cancer cells or patients treated with a particular oncolytic virus strain have been reported. The differences in responses may be explained by the tremendous variability of specific molecular defects in cancer cells. Apparently, an analysis of specific defects in antiviral defense mechanisms in individual cancer cases may facilitate a personalized prediction of clinical responses to oncolytic viruses and promote the introduction of this type of therapy to clinical practice.

**Figure 1:**
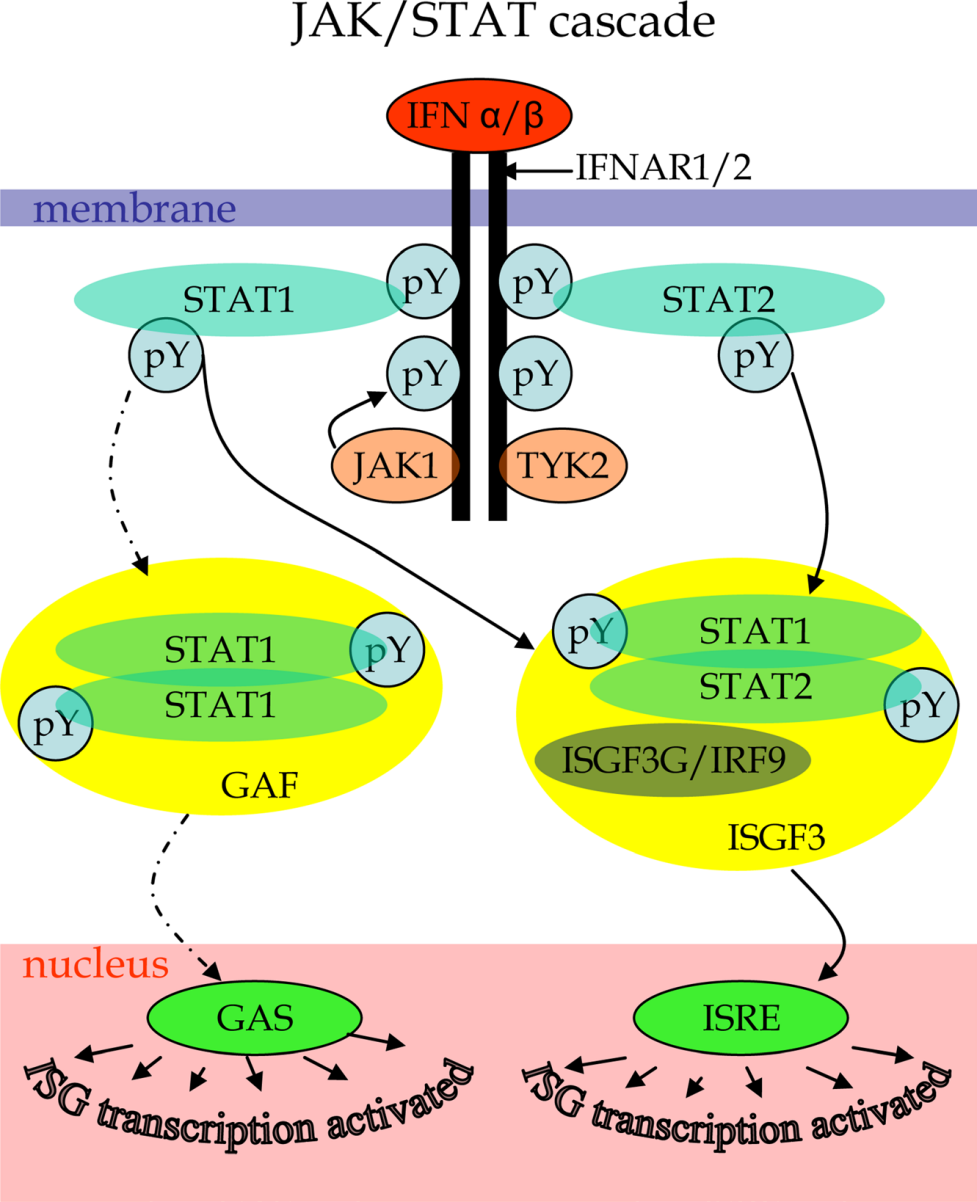
Scheme of canonical JAK/STAT cascade initiated by type I IFN binding to cytokine receptor as described elsewhere [50–51] Labels: pY – phosphorylated tyrosines.

Because of the still lethal prognosis, GBM is one of the forefront candidates for oncolytic virus therapy. To reveal specific defects affecting the sensitivity of GBM cells to viruses, we performed a comparative proteomics analysis of two GBM cell lines that differ in their responses to treatment with Type I IFN. The core of the signaling mechanism behind the IFN response is the JAK/STAT pathway [50, 51]. The JAK/STAT cascade (Figure 1) is initiated by an interaction of type I IFNs (IFNα or IFNβ) to cell-surface receptor molecules leading to their dimerization and formation of IFNAR1/2 complex that recruits Janus kinases JAK1 and TYK2. The activated JAK kinases promote the formation of complex between signal transducer and activator of transcription proteins STAT1 and STAT2 and its release from the intracellular portion of the IFN α/β receptor. The STAT heterodimers associate with the IFN regulatory factor ISGF3G/IRF-9 to form the ISGF3 transcription factor that in the nucleus activates IFN-stimulated genes (ISGs) by binding to specific IFN-stimulated response elements (ISREs) within the genes. The transcription of ISGs can be also affected by alternative branches of IFN induced pathways. For example, phosphorylated STAT1 can form homodimers acting as GAF transcription factor that activates ISGs transcription by binding to GAS elements. The JAK/STAT cascade can be negatively regulated by suppressors of cytokine signaling (SOCS), protein inhibitors of activated STATs (PIAS) and protein tyrosine phosphatases (PTPs), thus, mediating a downregulation of IFN signaling. Alteration of the pathways at different levels may result in deregulated responses to IFNs in malignant cells. However, there are branches of other signaling pathways that affect IFN response mechanisms and may be altered in cancer cells [45]. A treatment with Type I IFNs can induce apoptosis of some malignant cells in tumors by stimulating antitumor innate immune responses [52, 53]. However, IFNs can also mediate opposite effects, such as an increased survival of malignant cells [52, 54]. The ambiguities can be explained by different functional conditions of IFN signaling in malignant cells.

In the present study, we explored specific defects in antiviral mechanisms of two cell lines derived from GBM patients. One of the cell lines, DBTRG-05MG, was found to demonstrate a strong response to IFN-a treatment, which completely protected the cells from infection with vesicular stomatitis Indiana virus (VSV), while the A-172 GBM cell line retained high sensitivity to the virus regardless of the IFN treatment. By applying a shotgun LC-MS/MS-based proteomics and label-free quantitation of identified proteins, we mapped proteomes of both cell lines and analyzed the changes in protein expressions after IFN-a treatment. The revealed changes in protein components of IFN signaling reflect the molecular alterations that suggest being responsible for the individual sensitivity/resistance of GBM patients to oncolytic viruses.

## RESULTS AND DISCUSSION

### Analysis of changes in protein abundances induced by the IFN treatment

**Figure 2:**
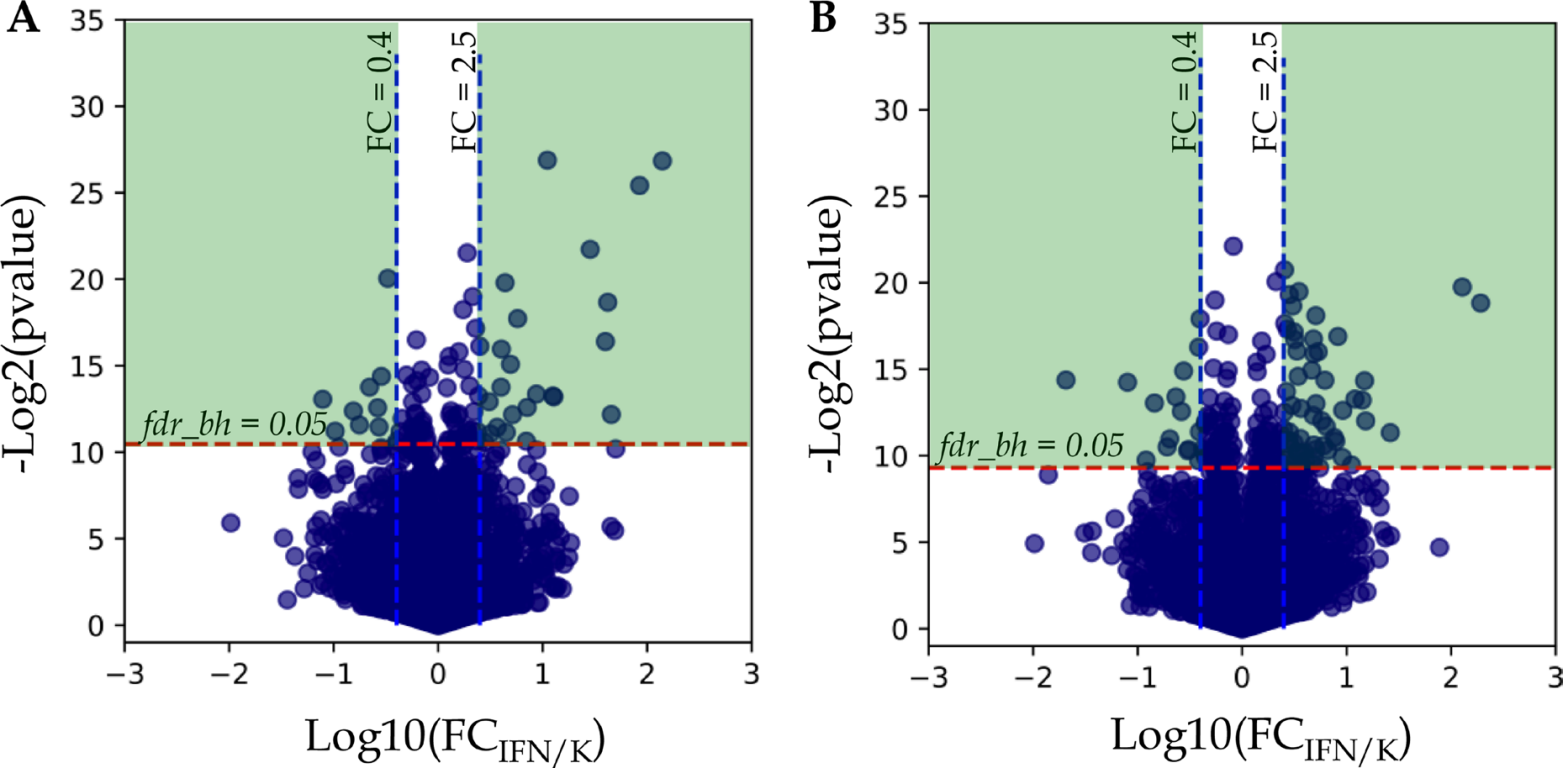
Analysis of protein fold changes in Volcano plots (**A**) A-172 cell line sensitive to VSV after IFN treatment; (**B**) DBTRG-05MG cell line resistant to VSV after IFN treatment. Legends: FC_IFN/K_, FC – fold change expressed as a ratio of the average protein normalized spectral index in IFN treated samples to the average spectral index of the same protein in control samples; *p*-value – *t*-test uncorrected *p*-value; *fdr*_BH_ – Benjamini-Hochberg FDR. *Green areas* denote differentially expressed proteins with Benjamini-Hochberg FDR < 0.05 and abundance fold changes ≥ 2.5 and ≤ 0.4 for up-regulated and down-regulated proteins, respectively.

Statistical analysis using paired *t*-test for dependent samples (*workflow A,* see M&M) revealed 109 and 199 proteins with Benjamini-Hochberg false discovery rate *fdr*_BH_ < 0.05 for A-172 and DBTRG-05MG, respectively. To understand how the calculated *p*-values are correlated with abundance fold changes (*FC*) for all proteins examined in *t*-test, the uncorrected protein *p*-values were plotted against the *FC* in logarithmic scale, −*log*_2_ *(pvalue) vs. log*_10_ *(FC*_IFN/K_) (Figure 2).

As shown in Figure 2A and 2B, there is a group of proteins above the threshold *fdr*_BH_ = 0.05, but the abundance fold changes for many of these proteins do not diverge significantly, and it ranges from 0.4 to 2.5. Based on this observation, we considered only proteins with abundance fold changes *FC* ≤ 0.4 and *FC* ≥ 2.5 for down- and up-regulation, respectively, for the further analysis (Figure 2, *Green Areas*).

### Comparison of statistical workflows

Since an application of the paired *t*-test to both normally distributed (ND) and not normally distributed (NND) data (*workflow A*) can generate inaccurate *p*-values for the NND data, and, thus, bias results, we split the data into two groups, ND and NND, based on the results of the Shapiro-Wilk test. At the next step, the paired *t*-test and the Kruskal-Wallis test were respectively applied to the ND and NND data and the results for each group were corrected for multiple comparisons before being combined (*workflows B and C*). *Workflow C* also reflects the effect from the imputation of missing protein identifications. Comparison of the workflows is shown in Venn diagrams in SI Supplementary Figure 1. Notably, results of the paired *t*-test applied to both ND and NND data were supported at least by either *workflow B* or *C*; *workflow B* actually contains only the results of paired *t*-test applied to ND data, because the correction of results of the Kruskal-Wallis test for multiple comparisons did not pass any proteins. The proteins identified as differentially expressed according to *workflow A* and *B* are provided in Supplementary Table 1. Relative protein quantities measured in the control and treated samples and processed using *workflow A* visibly differ (Supplementary Table 1). Note, that results obtained using the paired *t*-test (ND+NND, *fdr*_BH_ < 0.05) (*workflow A*) contain 15% and 9% of proteins with NND abundances for A-172 and DBTRG-05MG lines, respectively. Most of these proteins (exceptions: O75534-2 in A-172 and P07602-3 in DBTRG-05MG) were supported by running the *Kruskal-Wallis* test applied to NND data after the missing value imputation *(fdr*_BH_ *< 0.05)* (*workflow C*); Kruskal-Wallis *p*-values are also provided (Supplementary Table 1). Since the Kruskal-Wallis test is a non-parametric test, zeroing the missing protein identifications does not bias the results. Therefore, we are confident with the proteins identified using *workflow A*. Further analysis in the study is basically grounded on the results obtained using *workflow A*, if not stated otherwise.

### Studying the proteins with statistically significant changes in expression

When analyzing the proteins with statistically significant changes in expression, we divided proteins into two groups: (1) differentially expressed proteins identified both in IFN treated and in control samples; and (2) proteins identified either in IFN treated, or in control samples, only. Note that the first group includes all proteins inside the *Green Areas* in the Volcano plots (Figure 2), while the second group includes proteins with null abundance in either treated, or control samples, i.e. log_10_ FC _*IFN/K*_ cannot be determined for them.

**Figure 3:**
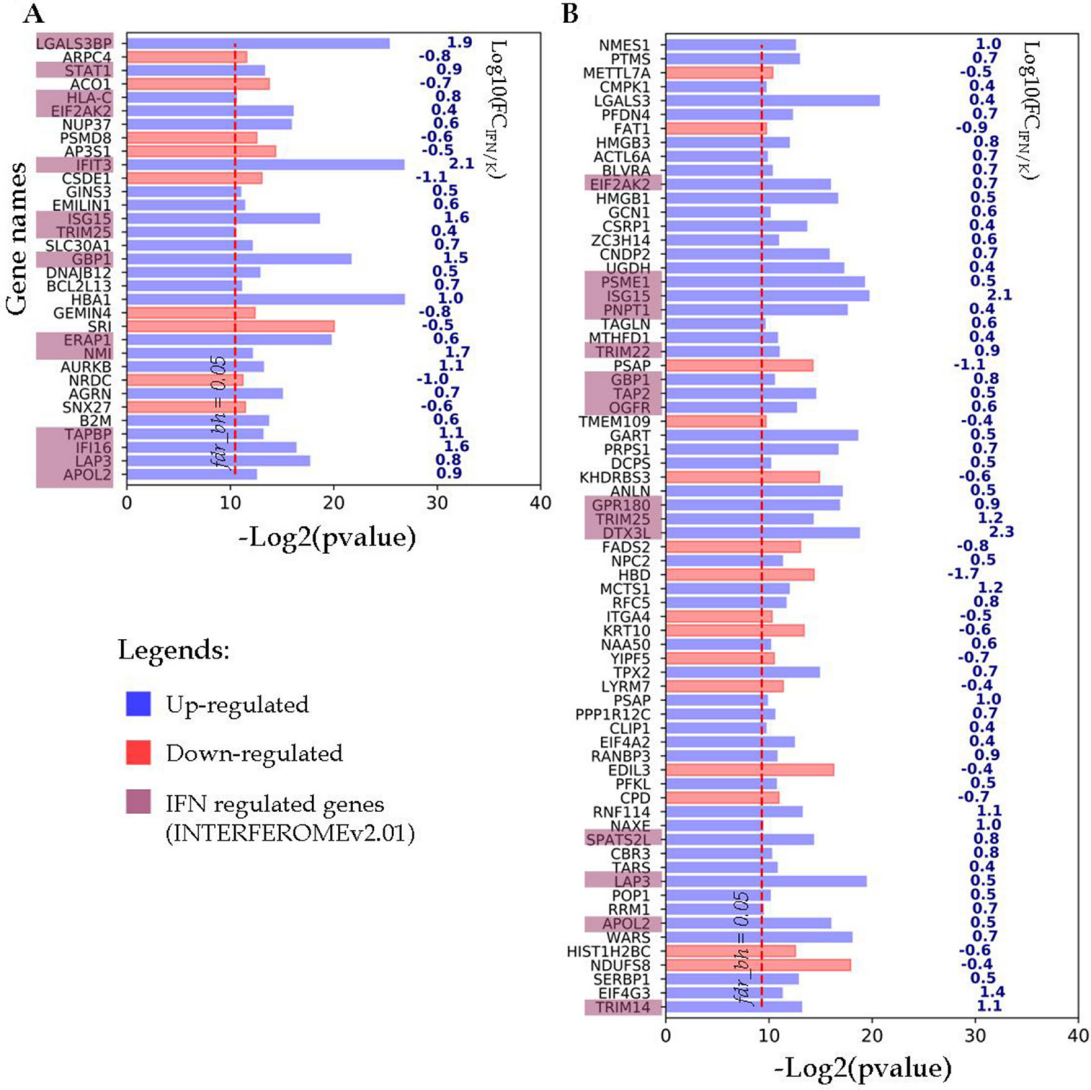
Proteins identified in both IFN-treated and control samples, change their abundance after IFN treatment (**A**) A-172 cell line sensitive to VSV after IFN treatment; (**B**) DBTRG-05MG cell line resistant to VSV after IFN treatment. Proteins satisfy criteria *fdr*_BH_ < 0.05, FC ≤ 0.4 and ≥ 2.5. The *p*-values and FC values characterize proteins encoded by the respective genes which are denoted at the Y axis.

The differentially expressed proteins from the first group and their encoding gene names are shown in Figure 3. As it can be seen for the A-172 cell line, 24 up-regulated and 9 down-regulated proteins with more than 2.5-fold differences in abundance have passed through the chosen filtration criteria (Figure 3A). Among the up-regulated proteins, 14 proteins were encoded by IFN-regulated genes. For the DBTRG-05MG cell line, 55 up-regulated and 15 down-regulated proteins were identified as responsive to IFN treatment (Figure 3B). Among the up-regulated proteins, 15 proteins are encoded by the IFN-regulated genes, according to the INTERFEROME db search.

**Figure 4:**
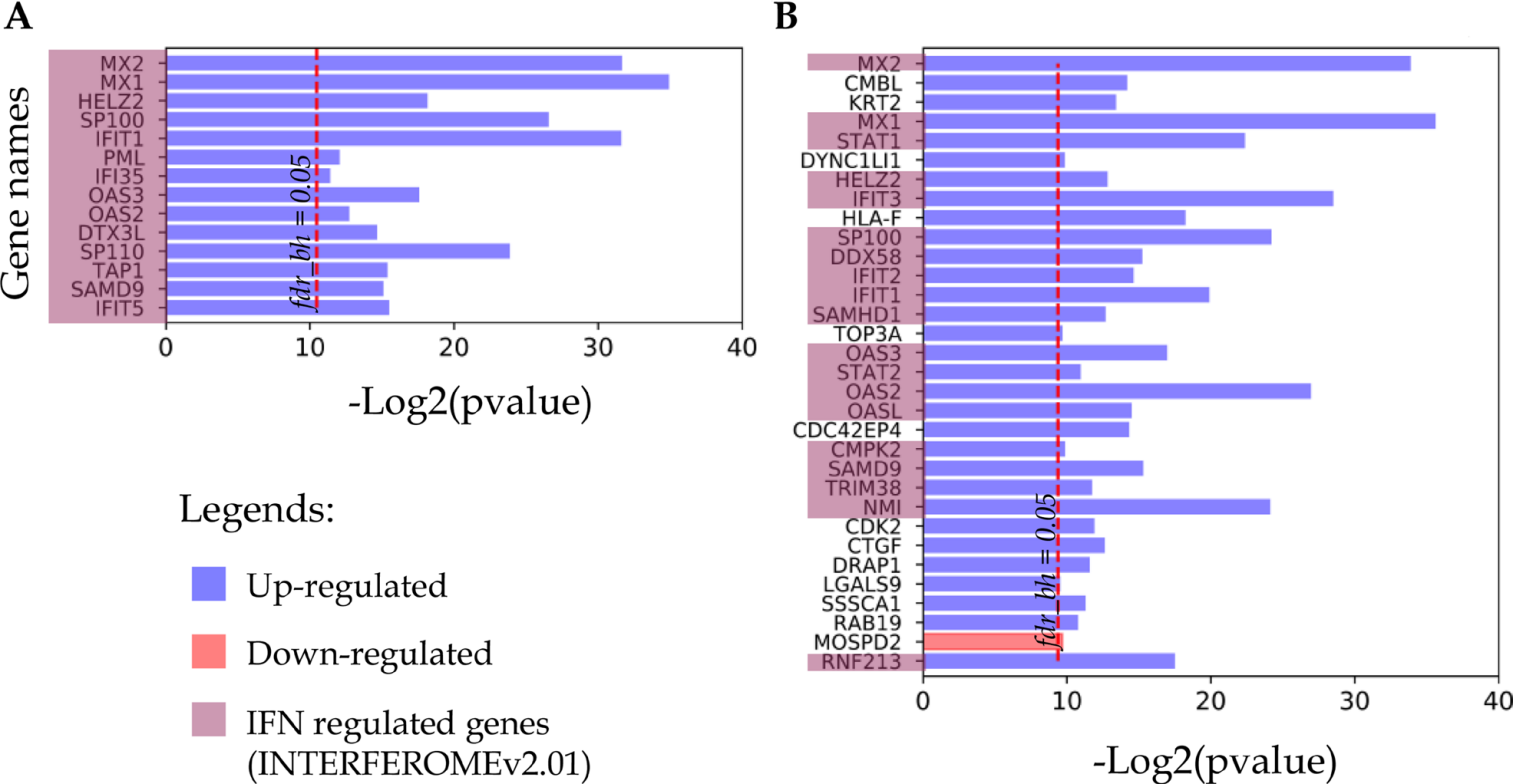
Proteins identified in either IFN treated samples or control samples (**A**) A-172 cell line sensitive to VSV after IFN treatment; (**B**) DBTRG-05MG cell line resistant to VSV after IFN treatment.

All proteins that changed their detection status (i.e. detected to non-detected and vice versa) in response to IFN treatment are listed in Figure 4. As noted above, for both cell lines this group contains up-regulated proteins, most of which are IRGs encoded. Thus, all 14 proteins identified only in the IFN-treated samples of the A-172 cell line were referenced as the IFN-regulated genes (Figure 4A). For the DBTRG-05MG cell line, 19 of 31 up-regulated proteins are the products of IRGs.

In summary, the following results for differentially expressed proteins satisfying Benjamini-Hochberg's criteria of FDR < 0.05; FC ≥ 2.5; FC ≤ 0.4 were obtained: 34 and 28 IRGs proteins were up-regulated after IFN treatment for DBTRG-05MG and A-172 cell lines, respectively. Thus, the number of IRGs proteins identified for the IFN-responsive DBTRG-05MG cells was higher by ~20% compared with the IFN-unresponsive A-172 cells. Note that the two glioblastoma cell lines have shared only ~40% of the identified IRGs proteins (Supplementary Figure 2).

The experimental evidences reported earlier have shown that the strength of antiviral defense correlates with the number of detected products of IFN-stimulated genes. It has been suggested that a larger number of IFN-stimulated genes is detected for the cells that exhibit stronger antiviral defenses [55]. Our results further support this hypothesis.

### Gene ontology (GO) analysis

To find the biological processes that were enhanced after the IFN treatment, gene ontology analysis of differentially expressed proteins satisfying criteria *fdr_BH_* < 0.05, FC ≤ 0.4 and ≥ 2.5 was performed. The proteins derived using statistical *workflows A* and *C* were analyzed using INTERFEROME, STRING and GOrilla databases [56–58]. The results are shown in Supplementary Table 2 and Supplementary Table 3 for workflows *A* and *C*, respectively.

**Figure 5:**
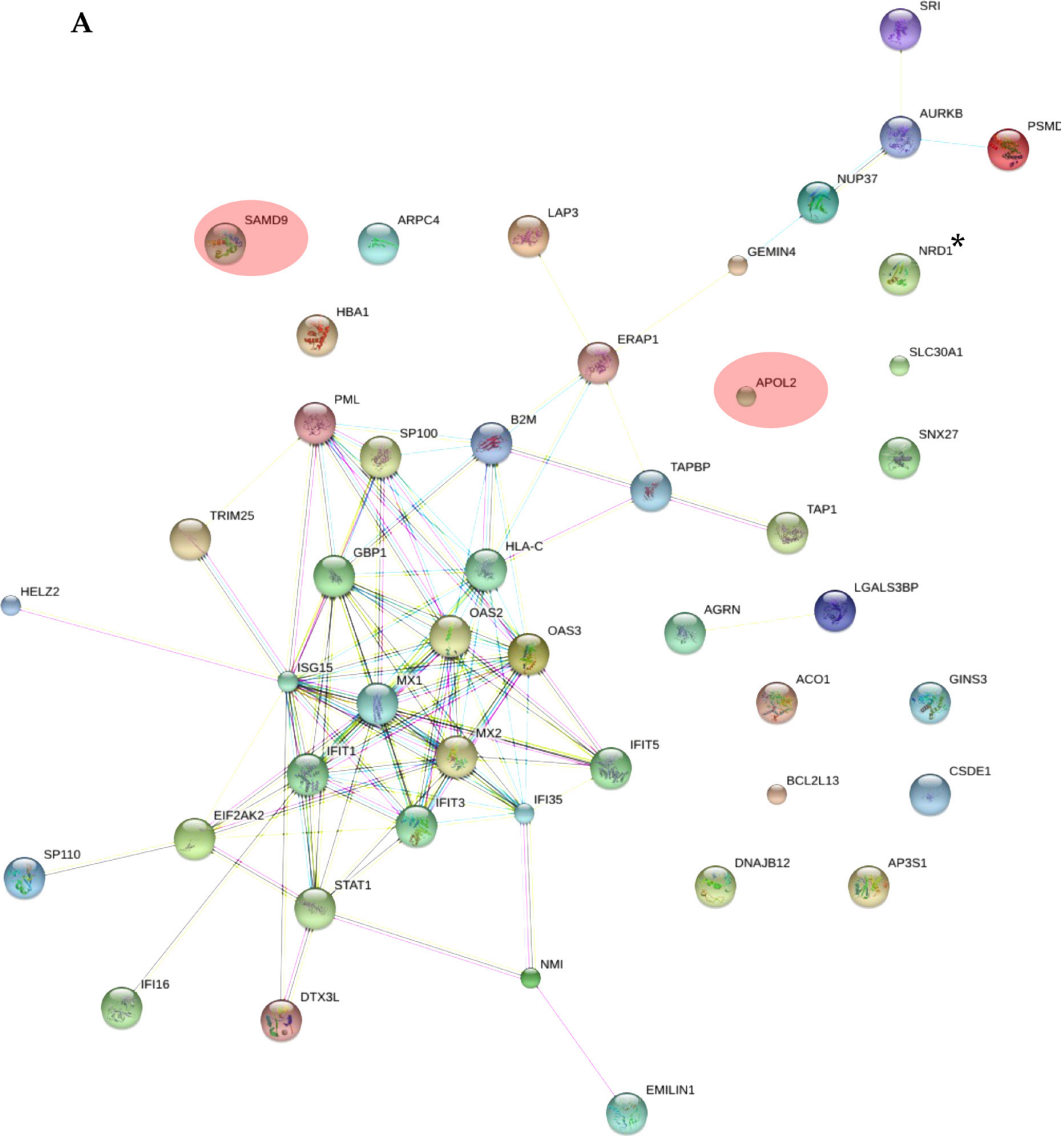

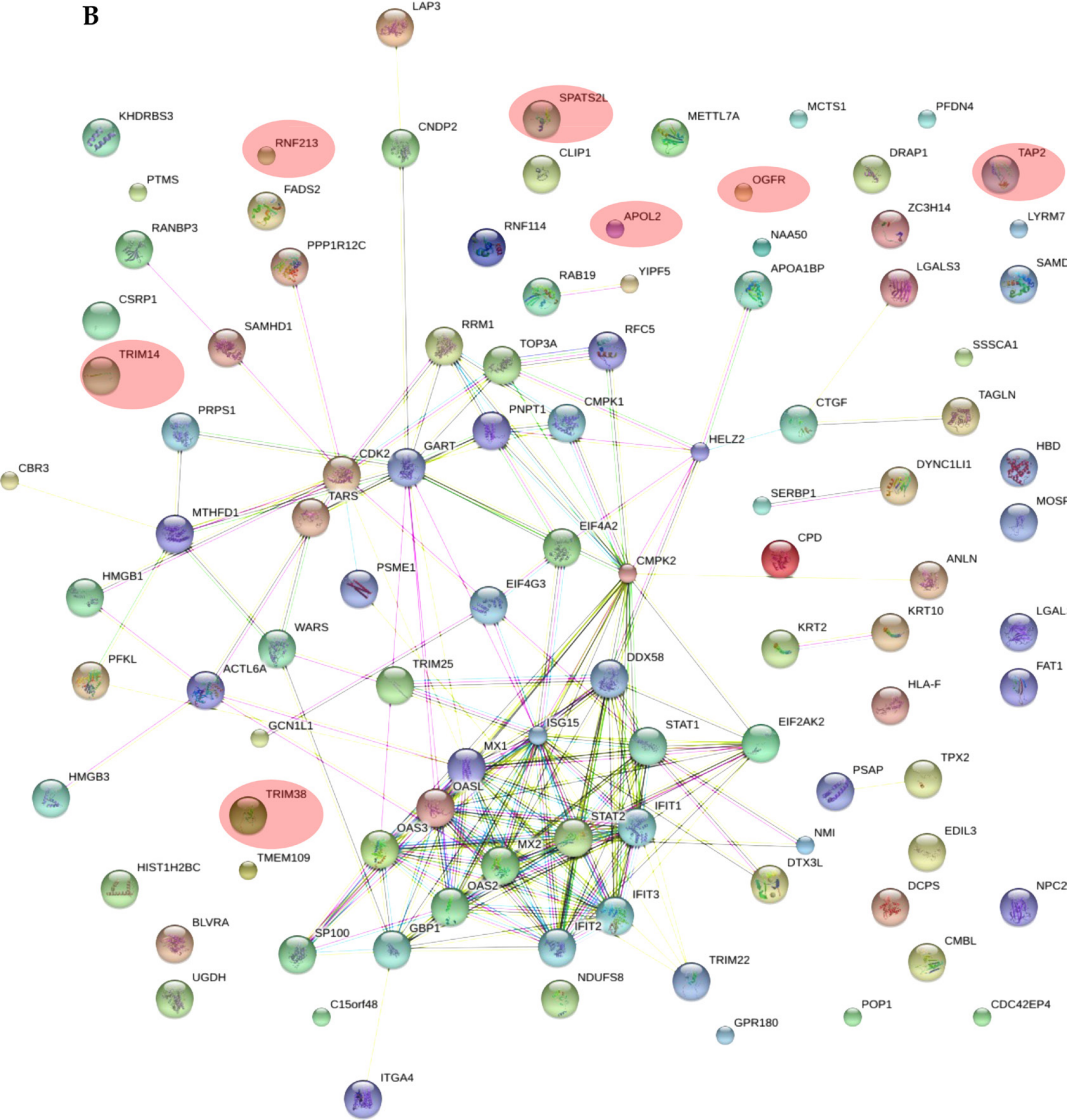
Protein-protein interaction networks for proteins differentially expressed in: (A) A-172 cell line, which is sensitive to VSV after type I IFN α-2b treatment; (B) DBTRG-05MG cell line, which develops resistance to VSV after type I IFN α-2b treatment Pink ovals denote the IRG-encoded proteins not involved in the interactome network. The networks were generated using STRING v.10.0. Proteins obtained using statistical workflow A were used in evaluation. ^*^ *Note*: Since nardilysin isoform 2 (NRD2) was not found in the STRING db, nardilysin NRD1 was submitted instead.

As shown in Supplementary Table 2, all the applied tools for both A-172 and DBTRG-05MG cell lines reveal that the prevailing changes belong to processes related to immune responses and IFN-mediated signaling. In addition, results for the A-172 cell line reveal antigen processing and presentation of peptide antigens by MHC class I processes. In case of the STRING database analysis, the antigen processing and presentation of peptide antigens by MHC class I processes were observed at higher false discovery rate of 0.02 and did not fall into top-20 biological processes. Protein-protein interaction networks for proteins expressed differentially in the treated samples were also generated (Figure 5A, 5B). The interaction networks for both cell lines cover most of the statistically relevant differentially expressed proteins. It can be noticed that most of the identified IRGs-encoded proteins are present in the interactome patterns: ~90% and ~80% for A-172 and DBTRG-05MG cell lines, respectively. In Figure 5, the networks cover ~70% and ~60% of all differentially expressed proteins identified for A-172 and DBTRG-05MG, respectively. Both networks can be considered as biologically related groups; however a functionally related protein group in the DBTRG-05MG cell line with preserved ability to develop antiviral resistance in response to IFN counts noticeably more proteins.

A GO analysis was also performed with the differentially expressed proteins identified in *workflow C*. As this workflow revealed significantly more proteins, we expected to get better insight into the pathways and clarify whether the enrichments within the MHC class I processes are specific to A-172 line. Results of the GO analysis (*workflow C*) are presented in Supplementary Table 3 (Supplementary Information). INTERFEROME and GOrilla provided very similar results revealing the top enriched processes for the A-172 cells line, that are cytokine signaling, defense / immune response and antigen processing and presentation of peptide antigens by MHC class I. The results agree well with the GO enrichments obtained from *workflow A* protein lists (Supplementary Table 2). However the results of STRING show noticeable differences, as the tool identifies first cellular component / organelle organization processes, instead of the IFN signaling and defense response (Supplementary Table 3). We assume that STRING could generate inaccurate *p*-values for the biological processes involving hundreds of proteins when the large target list (here from 500 to 600 proteins) is searched against the *whole genome* statistical background.

In summary, gene ontology analysis performed with three different tools identifies type I IFN signaling and defense response pathways as those showing major changes after IFN treatment. These pathways are the expected responders to the treatment. Besides, we suggest that the enrichment of the MHC class I antigen presentation process relates to the response to IFN treatment in A-172 cells that are not protected against VSV infection. These processes are considerably less stimulated by IFN in the IFN-responsive DBTRG-05MG cells. Reportedly, downregulation of MHC class I genes is responsible for the increased survival of cancer cells due to immune escape mechanisms [59]. The activation of STAT1 by IFNs may promote antitumor activity on oncolytic viruses *in vivo*, partly by upregulating MHC class I-mediated antigen presentation by tumor cells [60].

### Analysis of the type I IFN signaling in A-172 and DBTRG-05MG cell lines

We performed a comparative analysis of major components of the IFN response pathway to find functional differences between type I IFN signaling in A-172 and DBTRG-05MG cell lines. JAK/STAT (Figure 1) is the principal cascade behind the type I IFN signaling. The canonical scheme for this pathway includes several major components: IFNAR1/2 receptors, JAK1/TYK2 kinases, signal transducers and activators of transcription STAT1/2, as well as the IFN regulatory factor IRF9. A functional JAK/STAT cascade induces a transcription of multiple IFN-stimulated genes that drives the cell into the antiviral state in response to IFN treatment. Supplementary Table 4 shows matching major protein components of the JAK/STAT cascade within the proteomes of GBM lines under study. Specifically, interferons (IFNs), IFN receptors (INAR, INGR), Janus kinases (JAK, TYK), signal transducers and activators of transc ription (STAT), and interferon regulatory factors (IRF) were searched against the proteome of each cell line. Then, the protein's *q*-values and *t*-test's *p*-values for matched proteins were extracted from the search and *t*-test results as measures of protein false discovery rate and statistical significance of the abundance change. As the result, the STAT protein family can be highlighted due to its elevated presence in the treated samples and a regular identification in most runs of samples. Noteworthy, STAT1 and STAT2 from the STAT protein family were identified in all data sets obtained for the IFN-treated DBTRG-05MG line, while being absent in the control samples. That resulted in an extremely low *t*-test's *p*-values and statistically significant increase in the corresponding protein expression. The A-172 cell line exhibited different results for identification of proteins of the STAT family. Specifically, STAT1 was first identified in the untreated control samples. Then, it was found in the treated samples at significantly higher concentrations. STAT2 was not observed in untreated samples and was identified at < 0.01 protein FDR in half of all runs performed for the treated samples. Thus, in *workflow A*, STAT2 protein has not passed statistical threshold; therefore, its regulation was initially treated as unchanged.

**Figure 6:**
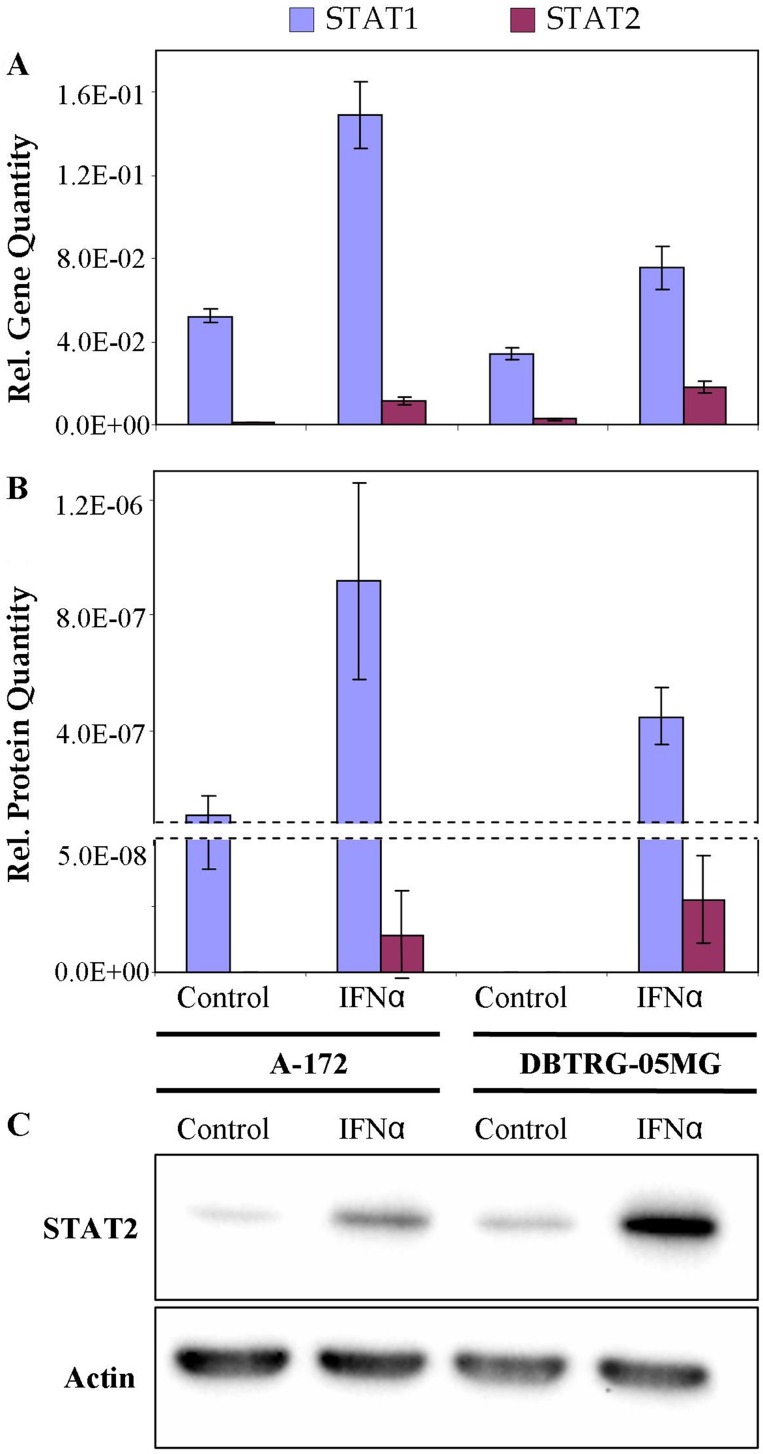
Relative quantities of STAT1 and STAT2 in IFN-resistant A-172 and IFN-sensitive DBTRG-05MG cell lines: real time qPCR (**A**), LC-MS/MS-based LFQ (**B**), western blot (**C**). Whiskers show standard deviations, ±σ. β-Actin equally expressed in the cells under study was used as a reference in western blot analysis.

Deficiency in the product of signal transducer and activator of transcription STAT2 for the VSV-sensitive (not protected by IFN) cell line could suggest a downregulation of the canonical JAK/STAT cascade that involves the formation of the ISGF3 complex required for the induction of antiviral state. To reveal the expression status of STAT1 and STAT2 genes, levels of corresponding transcripts were quantified by real-time qPCR. Relative levels of STAT2 protein were also measured by Western blot analysis. Results of real-time qPCR, LC-MS/MS-based label-free quantitation and Western blot analysis are compared in Figure 6. The results demonstrate that patterns of expression of the genes measured at the level of transcripts and proteins are consistent. Higher normalized expression levels of STAT1 can be noticed for both IFN-treated cell lines, compared with the lower expression levels of STAT2. By PCR measurement the STAT1 to STAT2 ratios were 4 and 13, for DBTRG-05MG and A-172 cells, respectively. In the IFN-treated cells STAT1 expression increased 2.0 and 2.8-fold, while expression levels of STAT2 increased 6.8 and 18.5-fold for DBTRG05MG and A-172 cells, respectively. Meantime, a normalized expression of STAT2 in the IFN-treated DBTRG-05MG cells was 1.6-fold higher, as compared to the IFN-treated A-172 cells, while the expression of STAT1 was 2-fold lower. Probably, a higher response of STAT1 in A-172 cells represents a compensatory effect due to the deficiency of STAT2. Results of western blot analysis clearly show a significantly higher level of total STAT2 protein in the IFN-treated DBTRG-05MG cells. Note that protein quantities in this study were measured for the whole cell extracts without specific analysis of protein modifications. Meantime, the quantities of phosphorylated STAT1 and STAT2 proteins correlate strictly with a regulation status of the JAK/STAT cascade. Thus, in A-172 cells, a downregulation of the direct signaling via ISGF3 complex formation and/or an upregulation of the minor pathway involving GAF element could be suggested. However, more studies are required to verify this hypothesis.

### Analysis of the defense responses in A-172 and DBTRG-05MG cells

**Figure 7:**
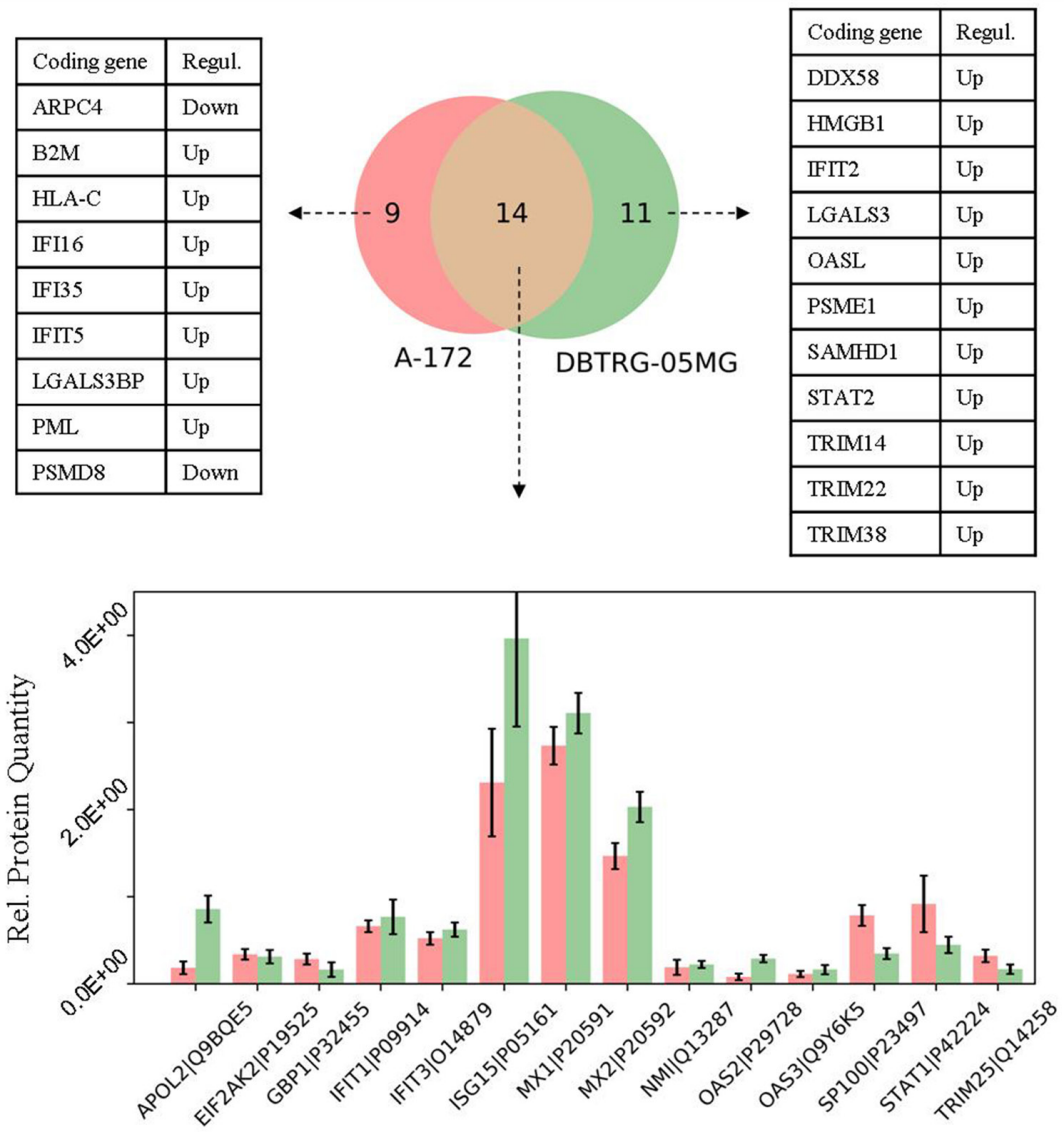
Comparison of the proteins related to defense response pathway (GO: 0006952) in the cell lines under study Whiskers show standard deviations: ±σ.

Next, in the GBM cells we compared expression profiles of proteins that belong to the defense response pathway (GO: 0006952). The use of STRING db has identified 23 and 25 protein-encoding genes that belong to this pathway and are increased in A-172 and DBTRG-05MG cells, respectively. The intersection between the observed genes is not high and comprises ~40% of all genes of the pathway (Venn diagram, Figure 7). The comparison for the two GBM cell lines has revealed a number of similarities and differences. For example, the MX, OAS, and EIF2AK2/PKR proteins comprise core proteins that reportedly protect against many different viruses [61]. The expression of MX and EIF2AK2 proteins were identified at similar levels for both cell lines, while the OAS proteins demonstrate a higher expression in the IFN-responsive DBTRG-05MG cells. Note that an up-regulation of OAS1 was found in the IFN-treated DBTRG-05MG cells at a level not reaching the statistical threshold, and the OAS1 protein was not detected in the A-172 samples (not shown in Figure 7). A product of the NMI (NMYC interactor) gene is capable of stimulating transcription of many STATs, except STAT2 [ http://www.genecards.org/cgi-bin/carddisp.pl?gene=NMI]. We found that the NMI protein has similar expression levels in both cell lines.

Proteins of the TRIM family are also expressed at higher level in the VSV-resistant (IFN-responsive) DBTRG-05MG cells. The upregulated proteins include TRIM25, a positive regulator of RIG-I/DDX58 helicase [62] that plays a key role in sensing viral components and initiating secretion of IFNs by infected cells. Meantime, ISG15 that can interact with IFIT1, MX1/MX2, EIF2AK2/PKR, and DDX58/RIG-I proteins is up-regulated at similar levels in both cell lines.

An isoform protein of PML gene (PML-2) was found increased in the IFN-treated A-172 cells, while this protein was not identified in the DBTRG-05MG cells. Reportedly, serine phosphorylation induces PML accumulation and sumoylation that is required for the pro-apoptotic activity of PML in response to a DNA damage (source: http://www.uniprot.org/uniprot/P29590). Nuclear autoantigen SP-100 was upregulated 2-fold in the VSV-sensitive A-172 cells. SP-100 and PML proteins are the main components of the PML bodies that are involved in a range of processes including tumor suppression, transcriptional regulation, apoptosis, DNA damage response, and antiviral defense [source: http://www.uniprot.org/uniprot/P23497].

Galectin-3 protein stimulating immune/defense response was found up-regulated in the DBTRG-05MG cells, while an up-regulation of galectin-3-binding protein LGALS3BP was observed in the A-172 cells. In the IFN-treated DBTRG-05MG cells the expression of LGALS3BP gene was detected at increased level, however it did not pass a statistical threshold (*Workflow A*). While all components of the IFN-dependent multiprotein complex comprising antiviral IFIT1, IFIT2, and IFIT3 proteins were found in the DBTRG-05MG cells, there were no statistically significant changes in the abundance of IFIT2 in the A-172 cells. However, IFIT5 protein was found at higher abundance in these cells.

In general, the comparative analysis has revealed higher expressions of proteins belonging to OAS, IFIT, and TRIM families in the DBTRG-05MG cells, as the main difference between the cells lines that can account for the differential sensitivity of GBM cells to viral infection after IFN treatment. Core components of JAK/STAT cascade, STAT1 and STAT2 proteins, were differentially expressed in the cell lines under study. STAT1 was observed at higher level in the IFN-treated A-172 cells compared to the DBTRG-05MG, while the expression level of STAT2 was higher in the IFN-treated DBTRG-05MG cells. The IFN-induced expression of PML-2 isoform implicated as a potential suppressor in tumors of central nervous system [63], is characteristic to A-172 cells. These results suggest that although A-172 cells have apparently lost the ability to respond to IFN by forming resistance to viral infection and have alterations within the JAK/STAT pathway, they still retain some IFN-mediated responses that might be beneficial for the malignant phenotype.

In conclusion, by LC-MS/MS analysis we compared proteomes of two GBM cell lines that exhibit different sensitivity to viral infection after type I IFN treatment. Of these, the A-172 cells were highly sensitive to VSV infection even after the treatment with IFN, and the DBTRG-05MG GBM cells demonstrated a strong protection against VSV infection even after treatment with low doses of IFN and seemingly retained the intact antiviral IFN response mechanisms. Label-free quantitation and statistical analysis of protein abundances performed for both IFN-treated and control samples have revealed the up-regulation of proteins encoded by the IFN-regulated genes in the treated cell lines. Specifically, the number of proteins encoded by IFN-regulated genes and identified for the DBTRG-05MG cell line that can be protected by IFN treatment from virus infection was up to 20% higher than those in the A-172 cells with a broken antiviral IFN response. GO analysis of proteins that are differentially expressed in response to IFN treatment has revealed the enrichment of biological processes related to type I IFN signaling and defense responses to viruses. In particular, an enrichment of the MHC class I-mediated antigen presentation was observed in IFN treated samples of virus-sensitive A-172 cells. Analysis of protein expression profiles has also revealed a lower expression of STAT2 protein in A-172 cells. Particularly, the change in abundance of STAT2 after IFN treatment was found statistically insignificant, and, thus, the A-172 cells can be considered STAT2-deficient, unlike the DBTRG-05MG cells. On the contrary, the relative abundance of STAT1 product was found at higher level in virus-unprotected A-172 cells. Presumably, the observed overexpression of STAT1 could be due to a compensatory process. In A-172 cells, a downregulation of the direct signaling via ISGF3 complex formation and an upregulation of the pathway involving GAF element could explain the compromised antiviral response. However, testing this hypothesis would require additional experiments to quantify active phosphorylated forms of STAT1 and STAT2 proteins, reflecting the actual status of IFN-signaling in the lines under study. Expression levels of STAT1 and STAT2 were also proved by real time qPCR and western blot analysis. For both cell lines, normalized gene expression coincided well with LC-MS/MS-based label-free quantitation of STAT1 and STAT2 proteins. This further suggests that the type I IFN signaling, particularly, the JAK/STAT cascade leading to the formation of ISGF3 complex, can be compromised in the VSV-sensitive A-172 cells. The revealed deficiency comes along with a lower number of IFN-stimulated genes encoding proteins with antiviral activity and, thus, a weaker antiviral defense response.

Changes in JAK-STAT pathway are common in cancer, as alteration in STATs not only modify cytokine-mediated responses, but also may affect epigenetic regulation, promote the self-renewal of niche formation for cancer stem cells, and assist in the establishment of the immunosuppressive tumor microenvironment. This is why the direct and mediated mechanisms of JAK-STAT signaling in tumor cells is becoming a favorite target for cancer therapy [64]. Our results indicate that changes in JAK-STAT pathway may also correlate with the increased sensitivity of cancer cells to oncolytic viruses. Further identifying most common changes in the pathway in connection with the functional state of IFN-mediated antiviral responses may provide useful biomarkers for predicting responses to oncolytic viruses in patients. We conclude that proteome analysis represents a potentially powerful tool for the identification of functional alterations within pathways that account for antiviral defenses of cancer cells. Certainly, a direct killing of cancer cells that can be seen in the *in vitro* models represents only part of the mechanisms underlying activities of oncolytic viruses in patients. Modulation of anticancer immunity, as well as virus-induced changes in tumor microenvironment may strongly affect the outcome of oncolytic virus therapy [18, 65]. However, the selective ability of cancer cells to support viral infection is believed to be of prime importance, ensuring multiple rounds of viral replication in the tumor and the induction of secondary events, including the immune-mediated attacks on cancer cells. This is why we believe that the analysis tested in this study could be extended to more tumor samples from GBM patients to find new expression biomarkers useful for predicting clinical responses to oncolytic virus therapy.

## MATERIALS AND METHODS

### Cell lines and virus sensitivity analysis

Two commercially available cell lines of GBM, DBTRG-05MG (ATCC^®^ CRL-2020™) and A-172 [A172] (ATCC® CRL-1620™) have been obtained from American Type Culture Collection. The cells were grown in DMEM medium (PanEco, Moscow, Russia) supplemented with 10% fetal bovine serum (Gibco, Thermo Fisher Scientific, Waltham, MA, USA). For IFN sensitivity tests, the cells were seeded at the density of 5000 cells/well to 96-well plates and incubated for 24 hrs at 37°C in 5% CO_2_ atmosphere. IFN α-2b (Farmaclon, Moscow, Russia) was added to samples at concentrations of 100, 200, 500, 1000, 2000, 5000 units/ml and incubated for the next 24 hours under same conditions. The cells were kept sub-confluent by the time of infection with VSV. The treated and untreated (control) samples were infected with VSV (Indiana strain) at the multiplicity of infection (MOI) of 1. Cytopathic effects (CPE) were estimated after 24 and 48 hrs of the treatment. IFN-treated DBTRG-05MG cells were found resistant to VSV (no CPE was observed even at minimal (100 units/ml) concentration of IFN α-2b). IFN-treated A-172 cells have demonstrated a complete degradation after 48 hrs of infection with VSV, even if pretreated with maximal dose (5000 units/ml) of IFN α-2b (Supplementary Figure 3). The DBTRG-05MG cell line was assumed to be an IFN-sensitive, in terms of the protection against VSV infection, and the A-172 cell line was found to be IFN-insensitive, meaning that it does not reach the antiviral state.

Two independently prepared sets of both DBTRG-05MG and A-172 cells, one treated with IFN α-2b at concentration of 100 units/ml for 24 hours, and the untreated control (C), were subjected for LC-MS/MS-based proteomic analyses. The sub-confluent cell cultures were grown and treated in 6 cm culture plates. The cells were scraped from the surface, washed 3 times with cold PBS (phosphate buffered solution), pelleted by low speed centrifugation and stored in liquid nitrogen until further use.

### Real time qPCR, relative quantitation

Real-time PCR was performed with SYBR Green qPCR master mix (USB) in a CFX96 Touch™ Real-Time PCR Detection System (Bio-Rad, U.S.A.). The PCR protocol was: initial activation at 95°C for 5 min, 40 cycles at 95°C for 10 s and 60°C for 40s. Ct values were converted into relative gene expression levels compared to that of internal control gene, GAPDH. Each PCR run was performed in quadruplicate. The primer sequences were as follows: STAT1-F, ATGGCAGTCTGGCGGCTGAATT; STAT1-R, CCAAACCAGGCTGGCACAATTG; STAT2-F, CAGGTCACAGAGTTGCTACAGC; STAT2-R, CGGTG AACTTGCTGCCAGTCTT.Primers were purchased from DNA-Synthesis Ltd. (Moscow, Russia).

### Western blot analysis

Cells were lysed with the M-PER^TM^ mammalian protein extraction reagent (Thermo Scientific, Germany) with addition of 1% cOmplete^TM^ EDTA-free protease inhibitor cocktail (Roche Diagnostics, Germany). The supernatant was separated by centrifugation at 13,000 rpm for 10 min at 4 °C. Total protein concentration was measured using Quick Start^TM^ Bradford protein assay kit (Bio-Rad, USA). Protein samples were mixed with 5×Laemmli loading buffer [66], denatured at 100°C for 5 min, then cooled and stored at −20°C until further use. Protein aliquots from each sample (10μg) along with a protein marker (PageRuler^TM^ Plus pre-stained protein ladder, Thermo Scientific, Germany) were separated by 12% SDS-PAGE, and transferred to a membrane (PVDF, Amersham^TM^ Hybond^®^ P, GE Healthcare) at 80V for 3 h. The electrophoresis and transfer to PVDF membrane were performed using Mini-PROTEAN Tetra cell (Bio-Rad, USA). Membranes were blocked in a phosphate buffered saline - Tween solution (PBST, 137 mM NaCl, 2.7 mM KCl, 10 mM Na_2_HPO_4_, 1.76 mM KH_2_PO_4_, pH 7.4, and 0.1% Tween-20) containing 5.0% of nonfat dry milk for 1 h at room temperature. Blotted membranes were then incubated with primary Rabbit monoclonal antibodies to Stat2 (D9J7L, Cell Signaling Technology, USA) at 1:1000 dilution, overnight at 4 °C, washed 3 times in PBST for 10 min, and incubated for 1 h at room temperature with secondary HRP-labeled mouse anti-Rabbit antibodies (Santa Cruz Biotechnology) at 1:5000 dilution. Finally, blots were rinsed in PBST 3 times for 10 min and visualized using ECL^TM^ Plus Western Blotting Detection System (Amersham^TM^, GE Healthcare) and ChemiDoc™ Imaging System (Bio-Rad, USA).

### Sample preparation for LC-MS/MS

Aliquotes of 10^6^ cells were resuspended in 100 μL of lysis buffer containing 0.1 % w/v ProteaseMAX Surfactant (Promega, USA) in 50 mM ammonium bicarbonate, and 10% v/v ACN. The cell lysate was stirred for 60 min at 1000 rpm at room temperature. Cell lysis was performed using ultrasonic homogenizer Bandelin Sonopuls HD2070 (Bandelin Electronic, Berlin, Germany). Cells were sonicated for 5 minutes at 30% amplitude on ice. The supernatant was collected after centrifugation at 13000 rpm for 10 min at room temperature (Centrifuge 5415R; Eppendorf, Hamburg, Germany). Total protein concentration was measured using Pierce quantitative colorimetric peptide assay (Thermo Scientific, Bremen, Germany). Protein extracts were reduced in 10 mM DTT at 56°C for 20 min and alkylated in 10 mM IAA at room temperature for 30 min in dark. Then, samples were overnight digested at 37°C using trypsin protease (Sequencing Grade Modified Trypsin, Promega, Madison, WI, USA) added at the ratio of 1:50 w/w. Enzymatic digestion was terminated by the addition of acetic acid (5% w/v). After the reaction was stopped, the sample was stirred (500 rpm) for 30 min at 45°C followed by centrifugation at 15 700× g for 10 min at 20°C (Centrifuge 5415R; Eppendorf). The supernatant was then added to the filter unit (10 kDa; Millipore, Billerica, MA, USA) and centrifuged at 13 400 × g for 20 min at 20°C. After that, 100 μL of 50% formic acid was added to the filter unit and the sample was centrifuged at 13 400× g for 20min at 20°C. Samples were dried using a vacuum concentrator at 45°C. Dried peptides were stored at −80°C until the LC-MS/MS analysis.

### LC-MS/MS analysis

Prior to LC-MS/MS analysis, the samples were desalted using Oasis cartridges for solid phase extraction (Oasis HLB, 1cc, 10mg, 30μm particle size, Waters). Then, the peptide concentration for each sample was measured using amino acid analysis. Loaded sample quantity was 1 μg per injection. LC-MS/MS analysis was performed using Orbitrap Fusion Lumos mass spectrometer (Thermo Scientific, San Jose, CA, USA) coupled with UltiMate 3000 nanoflow LC system (Thermo Scientific, Germering, Germany). Trap column μ-Precolumn C18 PepMap100 (5μm, 300μm i.d.x 5 mm, 100Å) (Thermo Scientific) and analytical column EASY-Spray PepMap RSLC C18 (2 μm, 75μm i.d. x 500 mm, 100Å) (Thermo Scientific) were employed for separations. Column temperature was set to 50°C. Mobile phases were as follows: (A) 0.1% FA in water; (B) 95% ACN, 0.1% FA in water. Samples were pre-concentrated for 10 min on a trap column at 5%B. Then, peptides were eluted using a linear gradient from 5%B to 20%B for 105 min followed by a linear gradient to 32%B for 15 min at 270 nL/min flow rate. The column was washed at 95%B for 10 minutes and equilibrated to the start concentration of mobile phase B.

Mass spectrometry measurements were performed using data-dependent acquisition (DDA) mode (Top Speed, 3s/cycle). Electrospray high voltage was set to 2.0 kV. Electrospray capillary temperature was 275°C. MS1 settings were as follows: mass range m/z from 375 m/z to 1,500 m/z, resolving power of 120,000 at m/z 200, maximum injection time was set to 50 ms, the automatic gain control (AGC) for MS1 was 4.0e5, the dynamic exclusion was set to 60 s. Precursor ions were isolated in the *m/z* window of 0.7 Th followed by their fragmentation using higher-energy collision dissociation (HCD) at normalized collision energy (NCE) of 30%. Fragment ions were measured in the Orbitrap mass-analyzer with resolving power of 30,000 at *m/z* 200. Maximum injection time during MS/MS was 60 ms with AGC value of 2.0e5.

### Protein identification and quantitation

Thermo raw files were converted to mgf format using a command line tool msсonvert distributed via ProteoWizard project (http://proteowizard.sourceforge.net/tools.shtml). Database search was performed using X!Tandem (version CYCLONE 2012.10.01.1) against the combined target-decoy database (db). The target db was SwissProt human (access date Dec 2016); the decoy db was compiled by reversing the protein sequences from the target db. Search parameters were as follows: 10 ppm for precursor mass tolerance; 0.01 Da for fragment mass tolerance; maximum 2 missed cleavage sites; fixed carboxyamidomethylated cysteines; potential methionine oxidation, phosphorylation on tyrosine, serine and threonine, and acetylation of lysine and protein N-terminus. Pepxmltk was used to convert tandem t.xml into pep.xml format [67]. Filtration of the results to 1.0% protein false discovery rate [68], post-search validation, as well as label free quantitation were performed using MPscore [69]. MPscore reported ~3000 and ~4000 protein groups for DBTRG-05MG and A-172 samples, respectively. Calculations of protein abundances using three label-free quantitation (LFQ) methods are also implemented in MPscore: normalized spectral index (SIN) [70], normalized spectral abundance factor (NSAF) [71] and exponentially modified protein abundance index (emPAI) [72]. To eliminate global technical biases, protein intensities were normalized. The results of this normalization showing no global shifts for the data are presented in Supplementary Figure 4. In this study, we evaluated the statistically significant changes in protein expression for the results obtained using all three quantitation methods. Data analysis using NSAF- and emPAI-calculated protein abundances resulted in almost identical patterns for differentially expressed proteins, while statistical analysis using SI_N_ method provided 20% and 30% larger amount of differentially expressed proteins for DBTRG-05MG and A-172 cell lines, respectively, as well as more enriched protein interaction networks. However, the use of either of the LFQ methods did not affect the main conclusions of the study. Therefore, here we show the results obtained using the SI_N_ method.

### Statistical analysis

Statistical analysis was performed using a Python module *statsmodels,* which implements many known statistical models (http://statsmodels.sourceforge.net/). The rationale for the statistical test choice is based on published recommendations [73]. Provided that our data originate from the same cell lines measured before and after the treatment, and that our goal is to identify protein changes occurring after the treatment, we have chosen a statistical test to compare two groups of dependent samples, namely, not treated against treated. According to [73], that could be the paired *t*-test for normally distributed (ND) data. For each identified protein, we evaluated statistically significant variations between ten measurements of the protein abundance in the control samples (5 LC-MS/MS technical replicates X 2 biological sample repetitions) and ten measurements of abundance for this protein in the IFN-treated samples (5 technical replicates X 2 biological repetitions). LFQ values for proteins unidentified in some sample replicates were either equaled to zero or imputed (see below). Corrections for multiple comparisons were taken into account. We considered Benjamini-Hochberg false discovery rate (FDR) for correcting *p*-values. Level of significance for corrected *p*-values was set to 0.05. To check normal distribution, the Shapiro-Wilk normality test was run for the data. Approximately 50% of the data has passed the normality test, while the remaining data has observed outliers, mainly due to missing measurements for some proteins. As recommended in [73], we run non-parametric tests for the data with not normal distribution (NND). Wilcoxon and Friedman tests have demonstrated a loss of statistical power due to the small sample size (10 measurements vs. ≥ 20 required), while Kruskal-Wallis test has shown a reasonable performance. Finally, we tested and compared three statistical workflows: (A) The paired *t*-test applied to both ND and NND data with the Benjamini-Hochberg FDR correction; (B) The paired *t*-test and the Kruskal-Wallis test followed by the Benjamini-Hochberg FDR correction, applied to the ND and NND data, respectively; and (C) The paired *t*-test (ND, *fdr*_BH_)/Kruskal-Wallis test (NND, *fdr*_BH_) applied to the data after imputation of missing protein identifications. One of the reasons for missing protein identifications is that the protein can be omitted by target-decoy filtering in some technical runs. To address this issue, we retrieved the unfiltered list of identified proteins for the proteins with missing values. If a protein was found in the unfiltered list, the missing value was imputed by the respective normalized spectral index. Otherwise, the protein was treated as absent or being under the detection limit and the missing value was equaled to zero. Such missing value strategy combined with a complementary evaluation of ND and NND data allowed revealing extra proteins and provided a better insight into the signaling pathways (Supplementary Information, Supplementary Table 2, 3). Note that the latter strategy can be considered as an analogue to lowering the threshold for filtering the results of statistical analysis. Comparison of statistical workflows and main results on differentially expressed proteins are provided in Supplementary Information (Supplementary Figure 1, Supplementary Tables 1–3). The strategy of a complementary use of the parametric and non-parametric statistical tests for an evaluation of ND and NND data is similar to the approach proposed in [74].

### Gene ontology and IFN-regulated genes (IRGs) analysis

Genes encoding the proteins with statistically significant changes in expression were searched against the INTERFEROME v2.01 database [72], containing type I, II, and III IFN-regulated genes, compiled using publicly available microarray datasets (http://interferome.its.monash.edu.au/interferome/home.jspx). Specified search parameters were as follows: type I IFNα, Homo sapiens, nervous system, brain. Additionally, differentially expressed proteins were analyzed using STRING v.10.0 database [57] to reveal physical and/or functional protein-protein interactions (http://string-db.org/). For evaluation, protein names were submitted as they are given in the reviewed SwissProt db; default statistical background was the whole genome. Also, GOrilla tool [58] was used for identifying enriched processes allowing an analysis of a target list of genes against the user-defined statistical background (http://cbl-gorilla.cs.technion.ac.il/). The combined proteome of treated and untreated lines (either A-172 or DBTRG-05MG, respectively) was set as a background.

## SUPPLEMENTARY MATERIALS FIGURES AND TABLES










