# Supplementary material for: Comparative proteomics as a tool for identifying specific alterations within interferon response pathways in human glioblastoma multiforme cells
Source: Oncotarget. 2017 Nov 29;9(2):1785–802. doi: 10.18632/oncotarget.22751 (PMC5788599; doi:10.18632/oncotarget.22751)
Supplement: Supplementary file 1 [file oncotarget-09-1785-s001.pdf]

Comparative proteomics as a tool for identifying specific alterations within interferon response pathways in human glioblastoma multiforme cells

SUPPLEMENTARY MATERIALS

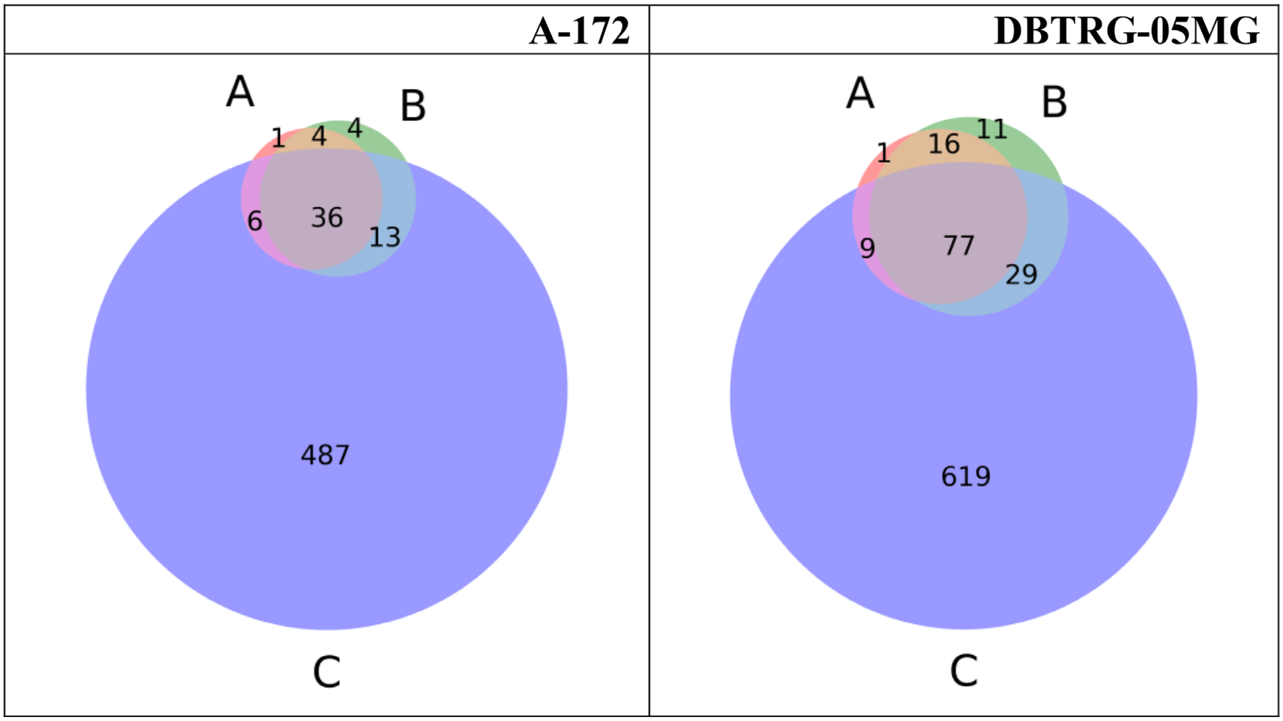

Supplementary Figure 1: Comparison between the statistical workflows. (**A**) Paired *t*-test applied to both ND and NND data / no missing value imputation, BH FDR < 0.05; (**B**) Paired *t*-test (ND data, BH FDR < 0.05) / Kruskal-Wallis test (NND, BH FDR < 0.05) / no missing value imputation; (**C**) Paired *t*-test (ND data, BH FDR < 0.05) / Kruskal-Wallis test (NND, BH FDR < 0.05) / missing value imputation. Venn diagrams show intersections between the differentially expressed proteins identified using workflows A, B and C.

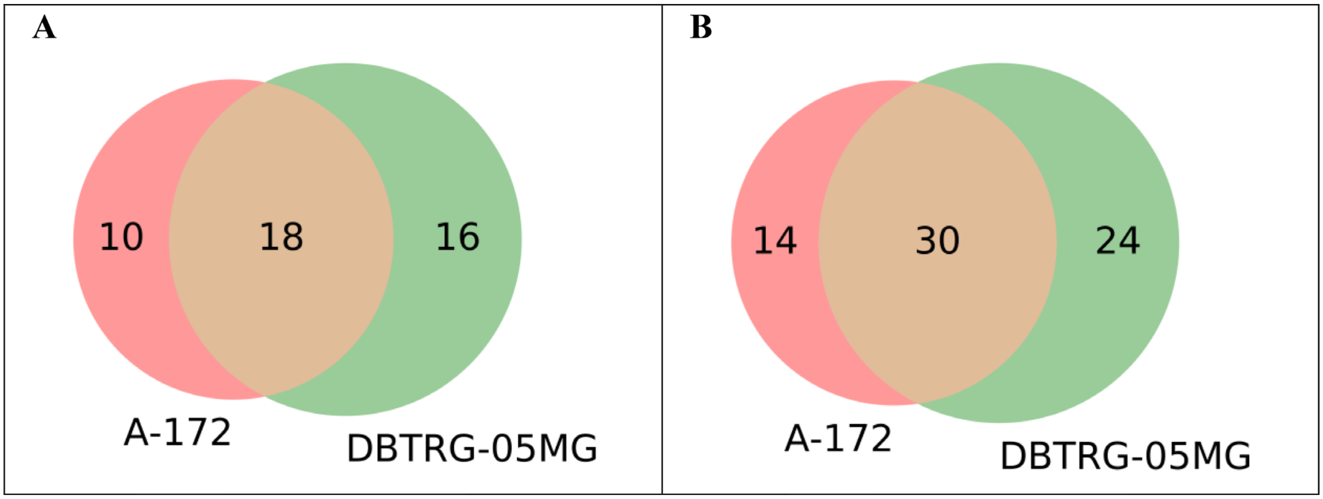

Supplementary Figure 2: Intersection of the identified IRGs proteins. (**A**) IRGs proteins identified among 47 and 103 differentially regulated proteins in A-172 and DBTRG-05MG, respectively, obtained using workflow A; (**B**) IRGs proteins identified among 542 and 734 differentially regulated proteins in A-172 and DBTRG-05MG, respectively, obtained using less conservative statistical workflow C.

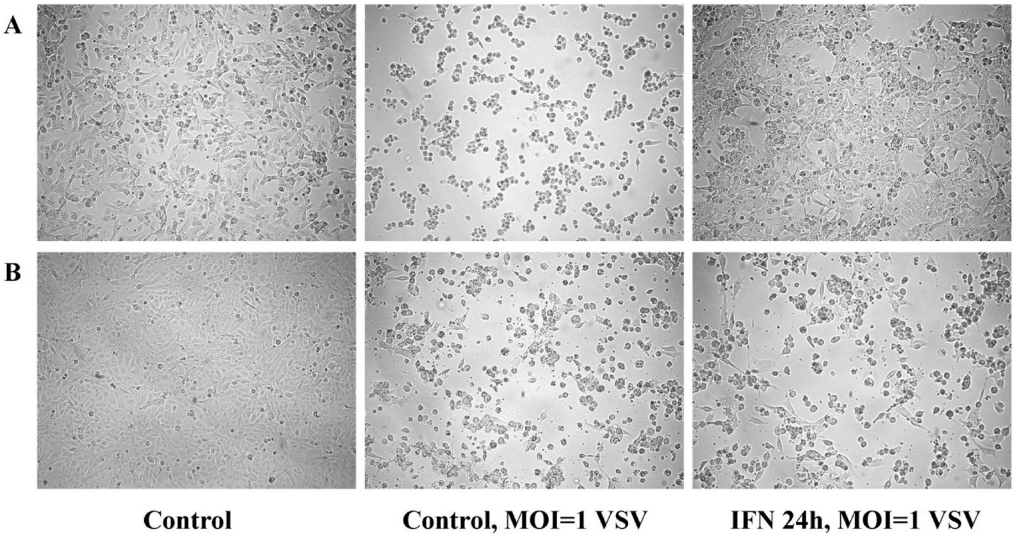

Supplementary Figure 3: Microphotographs of the control and IFN-treated samples before and after vesicular stomatitis virus infection. (**A**) DBTRG-05MG, (**B**) A-172.

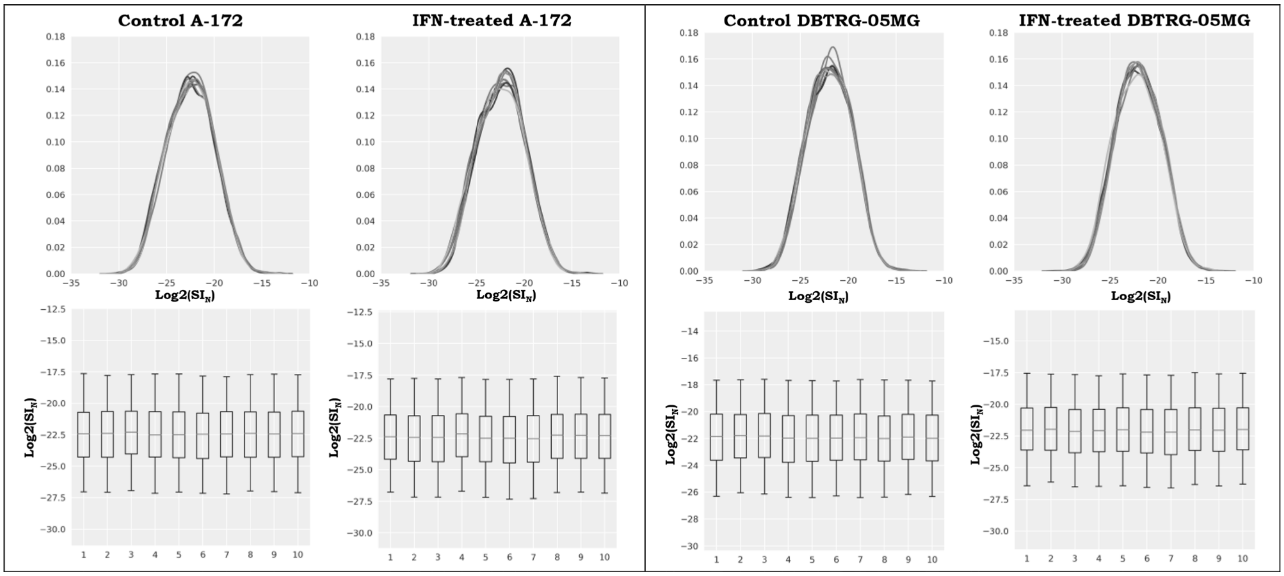

Supplementary Figure 4: Log2(SI_N) density plots and box-plots showing absence of technical bias between technical and biological replicates. Whickers in box-plots cover from 2.5 to 97.5 percentile.

Supplementary Table 1: Proteins with Benjamini-Hochberg FDR below 0.05: 109 and 199 proteins from A-172 and DBTRG-05MG glioblastoma lines, respectively. See Supplementary_Table_1

Supplementary Table 2: Enriched biological processes identified in glioblastoma lines. See Supplementary_Table_2

Supplementary Table 3: Results of GO analyses for A-172 and DBTRG-05MG lines evaluated using the combined statistical analysis Paired *t-test* (ND) / Kruskal-Wallis test (NND) after missing value imputation. See Supplementary_Table_3

Supplementary Table 4: Major protein components of the JAK/STAT cascade identified in glioblastoma proteomes. See Supplementary_Table_4
