# Supplementary material for: Comparative proteomics as a tool for identifying specific alterations within interferon response pathways in human glioblastoma multiforme cells
Source: Oncotarget. 2017 Nov 29;9(2):1785–802. doi: 10.18632/oncotarget.22751 (PMC5788599; doi:10.18632/oncotarget.22751)
Supplement: Supplementary file 2 [file oncotarget-09-1785-s002.docx]

**TABLE S1.** Entire sets of proteins derived basing on paired t-test, Benjamini-Hochberg FDR below 0.05. Differentially regulated proteins satisfy two criteria: Benjamini-Hochberg FDR < 0.05 and 2.5 ≤ FC ≤ 0.4. P-value (Shapiro-Wilk test) < 5.00E-02 means that null hypothesis on normal distribution should be rejected and paired t-test p-value can be inaccurate. Fractions of such proteins are 15 and 9% for A-172 and DBTRG-05MG lines, respectively. Differential expression of these proteins (exceptions: O75534-2 in A172 and P07602-3 in DBTRG05MG) was supported by *Kruskal-Wallis* test applied to NND data after missing value imputation *(BH FDR < 0.05)*; Kruskal-Wallis p-values are provided. Red and green rows highlight proteins falling out of intersections A∩B and (AUC)∩B, respectively, shown in Venn diagrams in Supplemental Figure S3.

Legends:

LFQ, SIn IFN –relative protein abundances measured in different replicates after the treatment;

LFQ, SIn Control – relative protein abundances measured in different replicates before the treatment;

FC, IFN/Control – protein abundance fold change, ratio of the means of normalized spectral indexes SI_N_ [DOI:10.1038/nbt.1592] in the IFN-treated and control samples.

| **A-172 (VSV-sensitive after IFN-alpha treatment)** | | | | | | | | | | |
| --- | --- | --- | --- | --- | --- | --- | --- | --- | --- | --- |
| **#** | **Uniprot I.D. \| Protein** | **Gene** | **p-value,**  **Shapiro**  **Wilk test** | **p-value, paired**  **t-test (ND + NND data)** | **Benjamini-Hochberg**  **FDR** | **p-value, Kruskal-Wallis test**  **(NND data)** | **LFQ,**  **SIn IFN** | **LFQ,**  **SIn Control** | **FC,**  **IFN/Control** | **Regulation** |
| **Proteins identified using *paired t-test (ND + NND data) / No Imputation* (Workflow A)** | | | | | | | | | | |
| 1 | Q16666\|IF16_HUMAN | IFI16 | 8.26E-05 | 1.16E-05 | 4.18E-03 | 4.88E-04 | ['3.37E-07', '3.44E-07', '3.59E-07', '3.28E-07', '3.40E-07', '0.00E+00', '3.32E-07', '3.92E-07', '3.38E-07', '4.19E-07'] | ['0.00E+00', '5.40E-08', '0.00E+00', '0.00E+00', '0.00E+00', '0.00E+00', '0.00E+00', '0.00E+00', '0.00E+00', '2.60E-08'] | 39.9 | Upregulated |
| 2 | P29590-8\|PML_HUMAN | PML | 6.01E-04 | 2.32E-04 | 2.75E-02 | 6.43E-04 | ['8.09E-07', '8.24E-07', '0.00E+00', '8.66E-07', '7.22E-07', '8.06E-07', '0.00E+00', '9.40E-07', '7.28E-07', '7.39E-07'] | ['0.00E+00', '0.00E+00', '0.00E+00', '0.00E+00', '0.00E+00', '0.00E+00', '0.00E+00', '0.00E+00', '0.00E+00', '0.00E+00'] | NA in controls | Upregulated |
| 3 | O75534-2\|CSDE1_HUMAN | CSDE1 | 6.54E-04 | 1.17E-04 | 1.85E-02 | 3.25E-04 | ['0.00E+00', '0.00E+00', '0.00E+00', '0.00E+00', '0.00E+00', '2.88E-07', '0.00E+00', '0.00E+00', '0.00E+00', '0.00E+00'] | ['4.18E-07', '3.99E-07', '0.00E+00', '3.79E-07', '3.98E-07', '3.48E-07', '3.96E-07', '4.38E-07', '4.85E-07', '4.06E-07'] | 0.079 | Downregulated |
| 4 | P59998\|ARPC4_HUMAN | ARPC4 | 2.00E-03 | 3.27E-04 | 3.54E-02 | 3.26E-04 | ['0.00E+00', '0.00E+00', '9.54E-07', '0.00E+00', '1.25E-06', '0.00E+00', '0.00E+00', '0.00E+00', '0.00E+00', '0.00E+00'] | ['1.28E-06', '1.47E-06', '1.02E-06', '1.33E-06', '1.11E-06', '1.09E-06', '1.26E-06', '1.33E-06', '1.50E-06', '1.10E-06'] | 0.176 | Downregulated |
| 5 | P42224\|STAT1_HUMAN | STAT1 | 2.50E-03 | 9.48E-05 | 1.63E-02 | 1.90E-03 | ['1.02E-06', '1.10E-06', '1.10E-06', '1.08E-06', '0.00E+00', '9.59E-07', '1.13E-06', '7.47E-07', '9.32E-07', '1.11E-06'] | ['1.46E-07', '9.12E-08', '0.00E+00', '1.39E-07', '1.95E-07', '8.44E-08', '0.00E+00', '1.27E-07', '1.85E-07', '8.67E-08'] | 8.7 | Upregulated |
| 6 | Q9HB58-3\|SP110_HUMAN | SP110 | 8.35E-03 | 6.46E-08 | 6.12E-05 | 5.34E-05 | ['2.37E-07', '2.53E-07', '3.04E-07', '3.18E-07', '2.26E-07', '2.41E-07', '2.41E-07', '3.83E-07', '2.28E-07', '2.25E-07'] | ['0.00E+00', '0.00E+00', '0.00E+00', '0.00E+00', '0.00E+00', '0.00E+00', '0.00E+00', '0.00E+00', '0.00E+00', '0.00E+00'] | NA in controls | Upregulated |
| 7 | P20591\|MX1_HUMAN | MX1 | 2.40E-02 | 3.07E-11 | 2.33E-07 | 5.34E-05 | ['2.83E-06', '2.65E-06', '2.90E-06', '2.94E-06', '2.34E-06', '2.60E-06', '2.90E-06', '2.88E-06', '2.92E-06', '2.38E-06'] | ['0.00E+00', '0.00E+00', '0.00E+00', '0.00E+00', '0.00E+00', '0.00E+00', '0.00E+00', '0.00E+00', '0.00E+00', '0.00E+00'] | NA in controls | Upregulated |
| 8 | P05161\|ISG15_HUMAN | ISG15 | 6.02E-02 | 2.40E-06 | 1.30E-03 |  | ['3.22E-06', '1.74E-06', '2.22E-06', '2.17E-06', '1.79E-06', '2.04E-06', '1.91E-06', '3.23E-06', '1.60E-06', '3.19E-06'] | ['0.00E+00', '3.17E-08', '0.00E+00', '1.56E-07', '1.15E-07', '0.00E+00', '0.00E+00', '0.00E+00', '1.72E-07', '7.67E-08'] | 41.9 | Upregulated |
| 9 | P30508\|1C12_HUMAN | HLA-C | 1.02E-01 | 6.28E-04 | 4.58E-02 |  | ['1.55E-06', '1.06E-06', '1.09E-06', '2.36E-06', '1.17E-06', '8.76E-07', '1.01E-06', '1.11E-06', '1.10E-06', '0.00E+00'] | ['8.47E-07', '0.00E+00', '4.00E-07', '0.00E+00', '0.00E+00', '0.00E+00', '3.79E-07', '0.00E+00', '0.00E+00', '0.00E+00'] | 7 | Upregulated |
| 10 | Q92572\|AP3S1_HUMAN | AP3S1 | 1.45E-01 | 4.69E-05 | 1.02E-02 |  | ['2.27E-07', '0.00E+00', '1.27E-07', '2.01E-07', '0.00E+00', '6.60E-08', '1.27E-07', '0.00E+00', '1.70E-07', '1.08E-07'] | ['2.76E-07', '3.43E-07', '4.94E-07', '2.86E-07', '2.30E-07', '4.43E-07', '4.14E-07', '2.63E-07', '4.18E-07', '4.21E-07'] | 0.286 | Downregulated |
| 11 | Q9BRX5-2\|PSF3_HUMAN | GINS3 | 1.57E-01 | 4.68E-04 | 3.92E-02 |  | ['4.50E-08', '3.63E-08', '3.27E-08', '4.05E-08', '0.00E+00', '7.27E-08', '3.43E-08', '5.03E-08', '4.11E-08', '4.00E-08'] | ['3.25E-08', '0.00E+00', '0.00E+00', '0.00E+00', '0.00E+00', '2.71E-08', '0.00E+00', '3.67E-08', '0.00E+00', '2.87E-08'] | 3.1 | Upregulated |
| 12 | Q08380\|LG3BP_HUMAN | LGALS3BP | 1.57E-01 | 2.23E-08 | 2.42E-05 |  | ['7.60E-07', '8.02E-07', '8.45E-07', '7.21E-07', '4.64E-07', '8.69E-07', '8.41E-07', '6.88E-07', '5.96E-07', '7.93E-07'] | ['3.05E-08', '0.00E+00', '3.90E-08', '0.00E+00', '1.76E-08', '0.00E+00', '0.00E+00', '0.00E+00', '0.00E+00', '0.00E+00'] | 84.7 | Upregulated |
| 13 | Q9BXK5\|B2L13_HUMAN | BCL2L13 | 2.13E-01 | 4.41E-04 | 3.85E-02 |  | ['6.30E-08', '9.42E-09', '2.62E-08', '4.43E-08', '7.60E-08', '5.19E-08', '5.24E-08', '7.23E-08', '5.89E-08', '4.64E-08'] | ['0.00E+00', '1.62E-08', '0.00E+00', '2.46E-08', '1.43E-08', '0.00E+00', '3.08E-08', '2.56E-08', '0.00E+00', '0.00E+00'] | 4.5 | Upregulated |
| 14 | Q9BQE5\|APOL2_HUMAN | APOL2 | 2.34E-01 | 1.63E-04 | 2.36E-02 |  | ['2.56E-07', '2.89E-07', '1.78E-07', '1.00E-07', '7.81E-08', '2.41E-07', '2.05E-07', '1.81E-07', '2.38E-07', '7.17E-08'] | ['1.76E-08', '2.64E-08', '0.00E+00', '1.90E-08', '4.04E-08', '2.31E-08', '2.55E-08', '9.20E-08', '1.44E-08', '0.00E+00'] | 7.1 | Upregulated |
| 15 | P21399\|ACOC_HUMAN | ACO1 | 3.04E-01 | 7.19E-05 | 1.33E-02 |  | ['6.29E-08', '7.30E-08', '3.05E-08', '0.00E+00', '6.69E-08', '2.13E-08', '9.05E-08', '3.59E-08', '4.67E-08', '5.78E-08'] | ['2.49E-07', '1.03E-07', '3.03E-07', '2.22E-07', '3.14E-07', '2.35E-07', '2.73E-07', '1.83E-07', '2.12E-07', '1.08E-07'] | 0.22 | Downregulated |
| 16 | P48556\|PSMD8_HUMAN | PSMD8 | 3.46E-01 | 1.65E-04 | 2.36E-02 |  | ['1.43E-07', '0.00E+00', '3.89E-08', '0.00E+00', '1.41E-07', '2.01E-07', '1.47E-07', '0.00E+00', '4.16E-08', '1.29E-07'] | ['3.86E-07', '1.73E-07', '3.37E-07', '5.01E-07', '4.42E-07', '2.86E-07', '4.37E-07', '1.47E-07', '1.61E-07', '3.26E-07'] | 0.263 | Downregulated |
| 17 | P30626\|SORCN_HUMAN | SRI | 3.51E-01 | 9.21E-07 | 6.35E-04 |  | ['1.37E-07', '9.40E-08', '9.27E-08', '2.33E-07', '1.27E-07', '1.21E-07', '1.17E-07', '1.98E-07', '1.62E-07', '1.16E-07'] | ['4.41E-07', '3.72E-07', '4.57E-07', '4.28E-07', '3.36E-07', '3.49E-07', '4.80E-07', '4.10E-07', '4.33E-07', '5.32E-07'] | 0.33 | Downregulated |
| 18 | Q9Y6K5\|OAS3_HUMAN | OAS3 | 3.88E-01 | 5.11E-06 | 2.15E-03 |  | ['1.25E-07', '1.86E-07', '7.49E-08', '1.46E-07', '6.47E-08', '1.15E-07', '1.11E-07', '6.72E-08', '1.22E-07', '1.23E-07'] | ['0.00E+00', '0.00E+00', '0.00E+00', '0.00E+00', '0.00E+00', '0.00E+00', '0.00E+00', '0.00E+00', '0.00E+00', '0.00E+00'] | NA in controls | Upregulated |
| 19 | Q9BYK8\|HELZ2_HUMAN | HELZ2 | 3.90E-01 | 3.41E-06 | 1.62E-03 |  | ['9.19E-09', '2.32E-08', '2.71E-08', '2.58E-08', '2.36E-08', '3.56E-08', '1.39E-08', '2.82E-08', '2.26E-08', '2.43E-08'] | ['0.00E+00', '0.00E+00', '0.00E+00', '0.00E+00', '0.00E+00', '0.00E+00', '0.00E+00', '0.00E+00', '0.00E+00', '0.00E+00'] | NA in controls | Upregulated |
| 20 | Q13287\|NMI_HUMAN | NMI | 3.92E-01 | 2.15E-04 | 2.67E-02 |  | ['0.00E+00', '1.59E-07', '2.04E-07', '2.02E-07', '1.38E-07', '1.93E-07', '2.21E-07', '2.49E-07', '3.66E-07', '1.65E-07'] | ['2.26E-08', '0.00E+00', '0.00E+00', '0.00E+00', '1.93E-08', '0.00E+00', '0.00E+00', '0.00E+00', '0.00E+00', '0.00E+00'] | 45.3 | Upregulated |
| 21 | Q5K651\|SAMD9_HUMAN | SAMD9 | 3.92E-01 | 2.83E-05 | 7.47E-03 |  | ['3.06E-08', '3.10E-08', '2.99E-08', '2.65E-08', '1.86E-08', '2.84E-08', '2.77E-08', '4.48E-08', '5.38E-08', '9.57E-09'] | ['0.00E+00', '0.00E+00', '0.00E+00', '0.00E+00', '0.00E+00', '0.00E+00', '0.00E+00', '0.00E+00', '0.00E+00', '0.00E+00'] | NA in controls | Upregulated |
| 22 | Q96GD4\|AURKB_HUMAN | AURKB | 3.99E-01 | 1.02E-04 | 1.69E-02 |  | ['7.36E-08', '6.51E-08', '1.23E-07', '9.24E-08', '6.07E-08', '1.16E-07', '6.15E-08', '1.45E-07', '7.71E-08', '1.47E-07'] | ['0.00E+00', '0.00E+00', '0.00E+00', '7.69E-08', '0.00E+00', '0.00E+00', '0.00E+00', '0.00E+00', '0.00E+00', '0.00E+00'] | 12.5 | Upregulated |
| 23 | P28838\|AMPL_HUMAN | LAP3 | 4.07E-01 | 4.62E-06 | 2.06E-03 |  | ['5.08E-07', '2.29E-07', '4.36E-07', '3.63E-07', '4.68E-07', '4.57E-07', '2.65E-07', '2.87E-07', '4.08E-07', '3.02E-07'] | ['8.00E-08', '6.66E-08', '9.28E-08', '4.77E-08', '2.73E-08', '4.64E-08', '6.74E-08', '6.16E-08', '8.40E-08', '7.32E-08'] | 5.8 | Upregulated |
| 24 | P09914\|IFIT1_HUMAN | IFIT1 | 4.14E-01 | 3.03E-10 | 7.66E-07 |  | ['6.46E-07', '6.26E-07', '6.79E-07', '7.32E-07', '5.68E-07', '5.88E-07', '5.77E-07', '7.38E-07', '7.04E-07', '7.62E-07'] | ['0.00E+00', '0.00E+00', '0.00E+00', '0.00E+00', '0.00E+00', '0.00E+00', '0.00E+00', '0.00E+00', '0.00E+00', '0.00E+00'] | NA in controls | Upregulated |
| 25 | P19525\|E2AK2_HUMAN | EIF2AK2 | 4.34E-01 | 1.40E-05 | 4.81E-03 |  | ['4.17E-07', '2.08E-07', '3.00E-07', '3.82E-07', '3.48E-07', '3.09E-07', '3.14E-07', '4.09E-07', '3.16E-07', '3.89E-07'] | ['1.92E-07', '1.56E-07', '1.11E-07', '1.26E-07', '2.01E-07', '1.03E-07', '1.29E-07', '1.92E-07', '1.06E-07', '3.73E-08'] | 2.5 | Upregulated |
| 26 | O43847-2\|NRDC_HUMAN | NRDC | 4.89E-01 | 4.20E-04 | 3.85E-02 |  | ['0.00E+00', '0.00E+00', '9.08E-09', '0.00E+00', '2.56E-08', '0.00E+00', '2.47E-08', '0.00E+00', '0.00E+00', '0.00E+00'] | ['4.41E-08', '6.38E-08', '4.07E-08', '1.13E-07', '8.40E-08', '1.51E-08', '4.82E-08', '7.99E-08', '5.92E-08', '2.54E-08'] | 0.104 | Downregulated |
| 27 | O14879\|IFIT3_HUMAN | IFIT3 | 4.94E-01 | 8.29E-09 | 1.26E-05 |  | ['4.00E-07', '4.34E-07', '5.00E-07', '5.76E-07', '4.73E-07', '5.33E-07', '4.75E-07', '6.14E-07', '6.17E-07', '5.92E-07'] | ['8.55E-09', '8.10E-09', '0.00E+00', '0.00E+00', '2.07E-08', '0.00E+00', '0.00E+00', '0.00E+00', '0.00E+00', '0.00E+00'] | 139.6 | Upregulated |
| 28 | Q03518\|TAP1_HUMAN | TAP1 | 5.47E-01 | 2.34E-05 | 6.56E-03 |  | ['1.74E-07', '7.74E-08', '1.25E-07', '1.23E-07', '1.01E-07', '6.22E-08', '6.77E-08', '9.84E-08', '4.56E-08', '7.65E-08'] | ['0.00E+00', '0.00E+00', '0.00E+00', '0.00E+00', '0.00E+00', '0.00E+00', '0.00E+00', '0.00E+00', '0.00E+00', '0.00E+00'] | NA in controls | Upregulated |
| 29 | P80217\|IN35_HUMAN | IFI35 | 5.76E-01 | 3.69E-04 | 3.68E-02 |  | ['6.27E-08', '2.24E-07', '2.71E-07', '0.00E+00', '4.31E-07', '2.65E-07', '1.99E-07', '2.54E-07', '2.67E-07', '1.40E-07'] | ['0.00E+00', '0.00E+00', '0.00E+00', '0.00E+00', '0.00E+00', '0.00E+00', '0.00E+00', '0.00E+00', '0.00E+00', '0.00E+00'] | NA in controls | Upregulated |
| 30 | P20592\|MX2_HUMAN | MX2 | 5.76E-01 | 2.92E-10 | 7.66E-07 |  | ['1.62E-06', '1.56E-06', '1.51E-06', '1.44E-06', '1.27E-06', '1.30E-06', '1.33E-06', '1.75E-06', '1.55E-06', '1.34E-06'] | ['0.00E+00', '0.00E+00', '0.00E+00', '0.00E+00', '0.00E+00', '0.00E+00', '0.00E+00', '0.00E+00', '0.00E+00', '0.00E+00'] | NA in controls | Upregulated |
| 31 | P29728\|OAS2_HUMAN | OAS2 | 5.83E-01 | 1.48E-04 | 2.20E-02 |  | ['6.05E-08', '0.00E+00', '5.93E-08', '8.12E-08', '1.39E-07', '5.94E-08', '1.28E-07', '6.55E-08', '1.02E-07', '1.00E-07'] | ['0.00E+00', '0.00E+00', '0.00E+00', '0.00E+00', '0.00E+00', '0.00E+00', '0.00E+00', '0.00E+00', '0.00E+00', '0.00E+00'] | NA in controls | Upregulated |
| 32 | Q9Y6M5\|ZNT1_HUMAN | SLC30A1 | 6.05E-01 | 2.17E-04 | 2.67E-02 |  | ['4.99E-08', '8.26E-08', '5.70E-08', '5.68E-08', '3.13E-08', '8.65E-08', '4.70E-08', '5.80E-08', '6.24E-08', '3.00E-08'] | ['0.00E+00', '2.31E-08', '0.00E+00', '0.00E+00', '0.00E+00', '0.00E+00', '2.05E-08', '0.00E+00', '3.72E-08', '2.84E-08'] | 5.1 | Upregulated |
| 33 | Q8NFH4\|NUP37_HUMAN | NUP37 | 6.08E-01 | 1.57E-05 | 5.19E-03 |  | ['1.39E-07', '1.73E-07', '1.07E-07', '1.64E-07', '1.56E-07', '1.48E-07', '1.85E-07', '2.56E-07', '1.15E-07', '1.62E-07'] | ['0.00E+00', '0.00E+00', '0.00E+00', '6.98E-08', '6.74E-08', '9.31E-08', '0.00E+00', '1.16E-07', '5.16E-08', '0.00E+00'] | 4 | Upregulated |
| 34 | P57678\|GEMI4_HUMAN | GEMIN4 | 6.52E-01 | 1.87E-04 | 2.57E-02 |  | ['0.00E+00', '1.90E-08', '0.00E+00', '1.63E-08', '0.00E+00', '0.00E+00', '0.00E+00', '0.00E+00', '1.26E-08', '0.00E+00'] | ['1.99E-08', '3.47E-08', '0.00E+00', '5.78E-08', '4.47E-08', '2.87E-08', '1.99E-08', '4.00E-08', '3.46E-08', '2.96E-08'] | 0.155 | Downregulated |
| 35 | Q8TDB6\|DTX3L_HUMAN | DTX3L | 6.89E-01 | 3.88E-05 | 8.91E-03 |  | ['2.78E-08', '1.05E-07', '8.11E-08', '8.17E-08', '7.10E-08', '4.82E-08', '1.15E-07', '7.33E-08', '7.82E-08', '1.56E-07'] | ['0.00E+00', '0.00E+00', '0.00E+00', '0.00E+00', '0.00E+00', '0.00E+00', '0.00E+00', '0.00E+00', '0.00E+00', '0.00E+00'] | NA in controls | Upregulated |
| 36 | Q9NZ08-2\|ERAP1_HUMAN | ERAP1 | 6.97E-01 | 1.10E-06 | 6.94E-04 |  | ['1.19E-07', '1.79E-07', '2.17E-07', '1.77E-07', '2.82E-07', '1.92E-07', '1.99E-07', '1.10E-07', '2.01E-07', '2.17E-07'] | ['4.02E-08', '7.52E-08', '2.29E-08', '1.29E-08', '7.97E-08', '4.96E-08', '4.56E-08', '5.99E-09', '3.21E-08', '6.96E-08'] | 4.4 | Upregulated |
| 37 | Q96L92\|SNX27_HUMAN | SNX27 | 7.13E-01 | 3.54E-04 | 3.65E-02 |  | ['0.00E+00', '7.53E-08', '0.00E+00', '1.17E-07', '6.41E-08', '0.00E+00', '4.17E-08', '0.00E+00', '0.00E+00', '1.39E-07'] | ['1.77E-07', '1.94E-07', '1.46E-07', '2.12E-07', '2.20E-07', '2.16E-07', '1.32E-07', '0.00E+00', '1.51E-07', '1.64E-07'] | 0.271 | Downregulated |
| 38 | Q14258\|TRI25_HUMAN | TRIM25 | 7.48E-01 | 6.57E-04 | 4.74E-02 |  | ['3.32E-07', '4.34E-07', '2.92E-07', '2.19E-07', '2.45E-07', '4.40E-07', '3.58E-07', '2.47E-07', '3.42E-07', '2.97E-07'] | ['1.24E-07', '7.19E-08', '3.28E-08', '2.05E-07', '1.39E-07', '2.91E-08', '9.94E-08', '2.08E-07', '1.46E-07', '9.61E-08'] | 2.8 | Upregulated |
| 39 | Q9NXW2\|DJB12_HUMAN | DNAJB12 | 7.57E-01 | 1.29E-04 | 1.98E-02 |  | ['1.06E-07', '1.26E-07', '1.67E-07', '1.31E-07', '1.70E-07', '2.26E-07', '2.54E-07', '1.82E-07', '2.87E-07', '1.49E-07'] | ['8.23E-08', '5.63E-08', '4.08E-08', '0.00E+00', '4.46E-08', '3.93E-08', '1.33E-07', '4.11E-08', '5.79E-08', '9.04E-08'] | 3.1 | Upregulated |
| 40 | Q9Y6C2\|EMIL1_HUMAN | EMILIN1 | 7.60E-01 | 3.60E-04 | 3.65E-02 |  | ['2.52E-08', '4.35E-08', '3.90E-08', '3.54E-08', '5.70E-08', '5.37E-08', '3.81E-08', '4.54E-08', '1.67E-08', '4.22E-08'] | ['0.00E+00', '1.93E-08', '0.00E+00', '1.06E-08', '2.28E-08', '0.00E+00', '2.21E-08', '0.00E+00', '2.23E-08', '1.10E-08'] | 3.7 | Upregulated |
| 41 | O15533-3\|TPSN_HUMAN | TAPBP | 7.98E-01 | 1.06E-04 | 1.71E-02 |  | ['2.70E-08', '3.47E-07', '1.62E-07', '2.58E-07', '2.06E-07', '2.29E-07', '1.59E-07', '1.70E-07', '2.73E-07', '1.84E-07'] | ['0.00E+00', '0.00E+00', '0.00E+00', '0.00E+00', '0.00E+00', '0.00E+00', '8.60E-08', '0.00E+00', '7.14E-08', '0.00E+00'] | 12.8 | Upregulated |
| 42 | P23497\|SP100_HUMAN | SP100 | 8.30E-01 | 9.96E-09 | 1.26E-05 |  | ['7.02E-07', '7.51E-07', '8.00E-07', '1.02E-06', '7.83E-07', '5.49E-07', '8.52E-07', '8.47E-07', '6.90E-07', '8.56E-07'] | ['0.00E+00', '0.00E+00', '0.00E+00', '0.00E+00', '0.00E+00', '0.00E+00', '0.00E+00', '0.00E+00', '0.00E+00', '0.00E+00'] | NA in controls | Upregulated |
| 43 | Q13325\|IFIT5_HUMAN | IFIT5 | 9.00E-01 | 2.16E-05 | 6.31E-03 |  | ['1.97E-07', '1.69E-07', '9.37E-08', '2.44E-07', '6.53E-08', '1.52E-07', '1.60E-07', '1.56E-07', '3.04E-07', '2.08E-07'] | ['0.00E+00', '0.00E+00', '0.00E+00', '0.00E+00', '0.00E+00', '0.00E+00', '0.00E+00', '0.00E+00', '0.00E+00', '0.00E+00'] | NA in controls | Upregulated |
| 44 | O00468\|AGRIN_HUMAN | AGRN | 9.24E-01 | 2.88E-05 | 7.47E-03 |  | ['2.84E-08', '3.58E-08', '4.74E-08', '4.70E-08', '5.02E-08', '3.54E-08', '5.98E-08', '3.26E-08', '3.62E-08', '3.33E-08'] | ['2.10E-08', '7.37E-09', '0.00E+00', '1.27E-08', '1.31E-08', '7.87E-09', '5.12E-09', '2.81E-09', '0.00E+00', '1.27E-08'] | 4.9 | Upregulated |
| 45 | P32455\|GBP1_HUMAN | GBP1 | 9.24E-01 | 2.88E-07 | 2.42E-04 |  | ['2.28E-07', '2.04E-07', '2.47E-07', '3.94E-07', '2.77E-07', '3.51E-07', '3.35E-07', '3.34E-07', '2.01E-07', '2.78E-07'] | ['0.00E+00', '0.00E+00', '0.00E+00', '0.00E+00', '2.51E-08', '3.47E-08', '0.00E+00', '2.35E-08', '1.67E-08', '0.00E+00'] | 28.5 | Upregulated |
| 46 | P61769\|B2MG_HUMAN | B2M | 9.79E-01 | 7.20E-05 | 1.33E-02 |  | ['3.21E-06', '6.70E-06', '5.08E-06', '3.51E-06', '5.18E-06', '5.52E-06', '4.72E-06', '3.17E-06', '2.42E-06', '4.63E-06'] | ['1.20E-06', '6.99E-07', '1.73E-06', '1.30E-06', '4.86E-07', '1.24E-06', '7.74E-07', '8.83E-07', '1.62E-06', '1.13E-06'] | 4 | Upregulated |
| 47 | P69905\|HBA_HUMAN | HBA1 | 9.84E-01 | 8.10E-09 | 1.26E-05 |  | ['1.55E-06', '1.61E-06', '1.54E-06', '1.92E-06', '1.50E-06', '1.28E-06', '1.72E-06', '1.75E-06', '1.57E-06', '1.86E-06'] | ['0.00E+00', '2.40E-07', '0.00E+00', '7.67E-08', '8.51E-08', '2.12E-07', '2.56E-07', '0.00E+00', '3.47E-07', '2.51E-07'] | 11.1 | Upregulated |
| **EXTRA proteins identified using *paired t-test (ND) + Kruskal-Wallis test (NND) / No Imputation* (Workflow B)** | | | | | | | | | | |
| 1 | O75882-3\|ATRN_HUMAN | ATRN | 1.28E-01 | 9.75E-04 | 3.35E-02 |  | ['0.00E+00', '8.42E-09', '4.74E-09', '4.67E-09', '8.23E-09', '4.43E-09', '3.86E-09', '3.74E-09', '0.00E+00', '4.30E-09'] | ['0.00E+00', '0.00E+00', '0.00E+00', '0.00E+00', '0.00E+00', '0.00E+00', '0.00E+00', '0.00E+00', '0.00E+00', '0.00E+00'] | NA in controls | Upregulated |
| 2 | P30490\|1B52_HUMAN | HLA-B | 6.57E-01 | 1.53E-03 | 4.47E-02 |  | ['9.11E-07', '7.83E-07', '1.11E-06', '2.14E-06', '7.42E-07', '6.48E-07', '8.61E-07', '1.00E-06', '6.79E-07', '8.75E-07'] | ['8.51E-07', '0.00E+00', '2.51E-07', '7.59E-07', '7.73E-07', '0.00E+00', '0.00E+00', '3.66E-07', '4.47E-07', '3.12E-07'] | 2.6 | Upregulated |
| 3 | P05187\|PPB1_HUMAN | ALPP | 2.52E-01 | 9.60E-04 | 3.35E-02 |  | ['0.00E+00', '0.00E+00', '1.09E-08', '0.00E+00', '0.00E+00', '0.00E+00', '1.25E-08', '0.00E+00', '0.00E+00', '0.00E+00'] | ['3.47E-08', '5.06E-08', '1.07E-08', '3.15E-08', '3.48E-08', '4.77E-08', '4.71E-08', '0.00E+00', '3.98E-08', '7.89E-08'] | 0.1 | Downregulated |
| 4 | P24941\|CDK2_HUMAN | CDK2 | 3.13E-01 | 8.98E-04 | 3.24E-02 |  | ['1.47E-07', '1.43E-08', '0.00E+00', '0.00E+00', '1.26E-07', '1.58E-07', '0.00E+00', '0.00E+00', '0.00E+00', '2.67E-07'] | ['3.40E-07', '2.87E-07', '0.00E+00', '3.22E-07', '2.66E-07', '2.76E-07', '3.14E-07', '1.75E-07', '2.68E-07', '2.69E-07'] | 0.3 | Downregulated |
| 5 | Q9UJK0\|TSR3_HUMAN | TSR3 | 4.14E-01 | 1.06E-03 | 3.45E-02 |  | ['0.00E+00', '3.51E-08', '0.00E+00', '0.00E+00', '0.00E+00', '0.00E+00', '3.72E-08', '0.00E+00', '2.51E-08', '0.00E+00'] | ['6.39E-08', '3.75E-08', '0.00E+00', '4.29E-08', '6.18E-08', '3.96E-08', '5.85E-08', '4.54E-08', '6.64E-08', '1.79E-08'] | 0.2 | Downregulated |
| 6 | Q9P2R3\|ANFY1_HUMAN | ANKFY1 | 5.97E-01 | 1.29E-03 | 3.98E-02 |  | ['9.90E-09', '5.31E-08', '5.07E-08', '4.68E-08', '3.65E-08', '6.00E-08', '1.94E-08', '4.07E-08', '1.40E-08', '3.23E-08'] | ['5.42E-09', '8.14E-09', '2.45E-08', '3.68E-08', '8.21E-09', '0.00E+00', '0.00E+00', '1.17E-08', '1.04E-08', '0.00E+00'] | 3.5 | Upregulated |
| 7 | P51003\|PAPOA_HUMAN | PAPOLA | 1.19E-01 | 7.66E-04 | 3.03E-02 |  | ['0.00E+00', '4.34E-08', '4.59E-08', '7.63E-08', '6.20E-08', '5.07E-08', '4.40E-08', '5.24E-08', '0.00E+00', '0.00E+00'] | ['1.53E-07', '8.81E-08', '8.78E-08', '1.44E-07', '1.28E-07', '8.37E-08', '1.14E-07', '1.21E-07', '7.56E-08', '0.00E+00'] | 0.4 | Downregulated |
| 8 | O75534-3\|CSDE1_HUMAN | CSDE1 | 8.49E-01 | 1.59E-03 | 4.61E-02 |  | ['1.93E-07', '5.04E-07', '3.45E-07', '3.60E-07', '3.12E-07', '0.00E+00', '3.04E-07', '2.34E-07', '2.52E-07', '1.33E-07'] | ['0.00E+00', '0.00E+00', '3.70E-07', '0.00E+00', '0.00E+00', '0.00E+00', '0.00E+00', '0.00E+00', '0.00E+00', '0.00E+00'] | 7.1 | Upregulated |
| 9 | P08962-3\|CD63_HUMAN | CD63 | 2.04E-01 | 7.97E-04 | 3.12E-02 |  | ['0.00E+00', '0.00E+00', '0.00E+00', '0.00E+00', '0.00E+00', '0.00E+00', '1.03E-07', '0.00E+00', '0.00E+00', '0.00E+00'] | ['9.53E-08', '3.94E-08', '9.44E-08', '1.16E-07', '8.69E-08', '1.58E-07', '2.10E-07', '0.00E+00', '1.16E-07', '0.00E+00'] | 0.1 | Downregulated |
| 10 | P02679\|FIBG_HUMAN | FGG | 4.54E-01 | 8.86E-04 | 3.24E-02 |  | ['7.03E-08', '3.50E-08', '0.00E+00', '5.39E-08', '5.54E-08', '4.66E-08', '5.00E-08', '0.00E+00', '7.71E-08', '7.18E-08'] | ['0.00E+00', '0.00E+00', '0.00E+00', '0.00E+00', '0.00E+00', '0.00E+00', '2.68E-08', '0.00E+00', '0.00E+00', '2.58E-08'] | 8.7 | Upregulated |
| 11 | O43402\|EMC8_HUMAN | EMC8 | 1.24E-01 | 8.53E-04 | 3.24E-02 |  | ['2.52E-07', '1.73E-07', '3.41E-07', '3.35E-07', '8.09E-08', '0.00E+00', '0.00E+00', '2.50E-07', '2.35E-07', '2.95E-07'] | ['0.00E+00', '0.00E+00', '0.00E+00', '3.93E-08', '0.00E+00', '0.00E+00', '0.00E+00', '0.00E+00', '0.00E+00', '0.00E+00'] | 49.9 | Upregulated |
| 12 | P01034\|CYTC_HUMAN | CST3 | 1.78E-01 | 8.94E-04 | 3.24E-02 |  | ['7.40E-08', '7.17E-08', '6.02E-08', '6.97E-08', '0.00E+00', '5.63E-08', '6.77E-08', '0.00E+00', '5.24E-08', '5.86E-08'] | ['4.34E-08', '3.67E-08', '0.00E+00', '4.82E-08', '0.00E+00', '0.00E+00', '0.00E+00', '0.00E+00', '0.00E+00', '0.00E+00'] | 4 | Upregulated |
| 13 | Q15042-4\|RB3GP_HUMAN | RAB3GAP1 | 4.29E-01 | 1.36E-03 | 4.10E-02 |  | ['1.46E-08', '0.00E+00', '0.00E+00', '0.00E+00', '7.75E-09', '0.00E+00', '0.00E+00', '0.00E+00', '0.00E+00', '9.15E-09'] | ['6.61E-08', '4.19E-08', '2.24E-08', '3.73E-08', '3.20E-08', '1.08E-07', '4.40E-08', '0.00E+00', '2.88E-08', '8.08E-08'] | 0.1 | Downregulated |
| 14 | Q15646\|OASL_HUMAN | OASL | 5.12E-01 | 1.64E-03 | 4.72E-02 |  | ['2.36E-08', '4.79E-08', '2.15E-08', '0.00E+00', '1.15E-08', '2.78E-08', '4.33E-08', '2.58E-08', '2.05E-08', '0.00E+00'] | ['0.00E+00', '0.00E+00', '0.00E+00', '0.00E+00', '0.00E+00', '0.00E+00', '0.00E+00', '0.00E+00', '0.00E+00', '0.00E+00'] | NA in controls | Upregulated |
| 15 | Q96A65\|EXOC4_HUMAN | EXOC4 | 8.18E-01 | 1.12E-03 | 3.60E-02 |  | ['0.00E+00', '0.00E+00', '5.56E-09', '2.70E-08', '6.63E-09', '1.19E-08', '0.00E+00', '9.40E-09', '1.51E-08', '0.00E+00'] | ['2.82E-08', '4.21E-08', '5.36E-09', '4.33E-08', '2.57E-08', '3.12E-08', '6.39E-09', '1.49E-08', '3.77E-08', '2.42E-08'] | 0.3 | Downregulated |
| 16 | Q8IVL6\|P3H3_HUMAN | P3H3 | 1.79E-01 | 1.05E-03 | 3.45E-02 |  | ['0.00E+00', '6.19E-08', '5.31E-08', '4.71E-08', '5.67E-08', '4.88E-08', '4.52E-08', '4.23E-08', '8.53E-08', '3.79E-08'] | ['0.00E+00', '0.00E+00', '0.00E+00', '0.00E+00', '0.00E+00', '4.71E-08', '0.00E+00', '0.00E+00', '6.43E-08', '2.25E-08'] | 3.6 | Upregulated |
| 17 | P36507\|MP2K2_HUMAN | MAP2K2 | 9.65E-01 | 8.14E-04 | 3.16E-02 |  | ['1.69E-07', '4.15E-08', '4.28E-08', '0.00E+00', '0.00E+00', '1.57E-07', '1.44E-07', '1.30E-07', '1.60E-07', '0.00E+00'] | ['2.95E-07', '3.43E-07', '1.12E-07', '4.28E-07', '3.12E-07', '1.41E-07', '2.77E-07', '3.22E-07', '4.96E-07', '2.45E-07'] | 0.3 | Downregulated |

| **DBTRG-05MG (VSV-resistant after IFN-alpha treatment)** | | | | | | | | | | |
| --- | --- | --- | --- | --- | --- | --- | --- | --- | --- | --- |
| **#** | **Uniprot I.D. \| Protein** | **Gene** | **p-value,**  **Shapiro**  **Wilk test** | **p-value,**  **paired t-test** | **Benjamini-Hochberg**  **FDR** | **p-value, Kruskal-Wallis test (NND data)** | **LFQ,**  **SIn IFN** | **LFQ,**  **SIn Control** | **FC,**  **IFN/Control** | **Regulation** |
| **Proteins identified using *paired t-test (ND + NND data) / No Imputation* (Workflow A)** | | | | | | | | | | |
| 1 | Q96DG6\|CMBL_HUMAN | CMBL | 1.45E-03 | 5.33E-05 | 6.17E-03 | 5.38E-05 | ['4.07E-08', '1.44E-07', '6.18E-08', '7.09E-08', '5.90E-08', '5.66E-08', '4.95E-08', '5.35E-08', '4.97E-08', '7.91E-08'] | ['0.00E+00', '0.00E+00', '0.00E+00', '0.00E+00', '0.00E+00', '0.00E+00', '0.00E+00', '0.00E+00', '0.00E+00', '0.00E+00'] | NA in controls | Upregulated |
| 2 | O00635\|TRI38_HUMAN | TRIM38 | 2.39E-03 | 2.91E-04 | 1.99E-02 | 6.43E-04 | ['0.00E+00', '2.93E-08', '2.65E-08', '3.03E-08', '2.36E-08', '1.80E-08', '0.00E+00', '2.69E-08', '2.68E-08', '3.02E-08'] | ['0.00E+00', '0.00E+00', '0.00E+00', '0.00E+00', '0.00E+00', '0.00E+00', '0.00E+00', '0.00E+00', '0.00E+00', '0.00E+00'] | NA in controls | Upregulated |
| 3 | Q9ULW0\|TPX2_HUMAN | TPX2 | 2.87E-03 | 3.16E-05 | 4.76E-03 | 2.11E-04 | ['8.96E-08', '1.02E-07', '9.07E-08', '1.33E-07', '1.41E-07', '9.59E-08', '1.14E-07', '1.08E-07', '6.71E-08', '1.13E-07'] | ['2.52E-08', '0.00E+00', '0.00E+00', '1.46E-08', '4.50E-08', '1.37E-08', '2.41E-08', '1.80E-08', '7.33E-08', '1.35E-08'] | 4.6 | Upregulated |
| 4 | Q9Y6K5\|OAS3_HUMAN | OAS3 | 8.24E-03 | 7.74E-06 | 1.79E-03 | 5.38E-05 | ['1.16E-07', '1.44E-07', '2.91E-07', '1.33E-07', '1.42E-07', '2.25E-07', '1.34E-07', '1.19E-07', '1.89E-07', '1.31E-07'] | ['0.00E+00', '0.00E+00', '0.00E+00', '0.00E+00', '0.00E+00', '0.00E+00', '0.00E+00', '0.00E+00', '0.00E+00', '0.00E+00'] | NA in controls | Upregulated |
| 5 | P02042\|HBD_HUMAN | HBD | 9.28E-03 | 4.73E-05 | 5.84E-03 | 3.25E-04 | ['0.00E+00', '0.00E+00', '0.00E+00', '0.00E+00', '0.00E+00', '1.24E-07', '0.00E+00', '0.00E+00', '0.00E+00', '0.00E+00'] | ['8.00E-07', '6.25E-07', '6.17E-07', '0.00E+00', '8.32E-07', '8.07E-07', '2.74E-07', '7.08E-07', '6.99E-07', '6.48E-07'] | 0.021 | Downregulated |
| 6 | P07602-3\|SAP_HUMAN | PSAP | 1.77E-02 | 1.04E-03 | 3.84E-02 | 9.23E-04 | ['1.23E-06', '1.84E-06', '1.09E-06', '0.00E+00', '1.66E-06', '1.88E-06', '1.56E-06', '1.63E-06', '1.21E-06', '2.06E-06'] | ['0.00E+00', '0.00E+00', '1.56E-06', '0.00E+00', '0.00E+00', '0.00E+00', '0.00E+00', '0.00E+00', '0.00E+00', '0.00E+00'] | 9.1 | Upregulated |
| 7 | Q15646\|OASL_HUMAN | OASL | 2.08E-02 | 4.33E-05 | 5.70E-03 | 1.95E-04 | ['4.98E-08', '8.25E-08', '1.24E-07', '1.03E-07', '9.91E-08', '1.20E-07', '9.15E-08', '1.17E-07', '1.20E-07', '0.00E+00'] | ['0.00E+00', '0.00E+00', '0.00E+00', '0.00E+00', '0.00E+00', '0.00E+00', '0.00E+00', '0.00E+00', '0.00E+00', '0.00E+00'] | NA in controls | Upregulated |
| 8 | Q14919\|NC2A_HUMAN | DRAP1 | 2.25E-02 | 3.27E-04 | 2.17E-02 | 6.43E-04 | ['0.00E+00', '1.39E-07', '0.00E+00', '1.34E-07', '1.85E-07', '1.12E-07', '1.10E-07', '1.57E-07', '1.33E-07', '1.61E-07'] | ['0.00E+00', '0.00E+00', '0.00E+00', '0.00E+00', '0.00E+00', '0.00E+00', '0.00E+00', '0.00E+00', '0.00E+00', '0.00E+00'] | NA in controls | Upregulated |
| 9 | Q8TCS8\|PNPT1_HUMAN | PNPT1 | 3.62E-02 | 4.89E-06 | 1.45E-03 | 1.57E-04 | ['7.98E-07', '7.72E-07', '7.11E-07', '7.31E-07', '9.84E-07', '9.22E-07', '7.86E-07', '1.10E-06', '7.95E-07', '9.25E-07'] | ['3.73E-07', '3.40E-07', '3.61E-07', '3.71E-07', '2.53E-07', '2.11E-07', '3.93E-07', '3.12E-07', '1.95E-07', '5.26E-07'] | 2.6 | Upregulated |
| 10 | P24941\|CDK2_HUMAN | CDK2 | 4.63E-02 | 2.58E-04 | 1.85E-02 | 5.38E-05 | ['2.61E-07', '1.36E-07', '2.29E-07', '2.50E-07', '2.05E-07', '4.86E-07', '5.01E-07', '1.42E-07', '2.20E-07', '9.63E-08'] | ['0.00E+00', '0.00E+00', '0.00E+00', '0.00E+00', '0.00E+00', '0.00E+00', '0.00E+00', '0.00E+00', '0.00E+00', '0.00E+00'] | NA in controls | Upregulated |
| 11 | O60232\|SSA27_HUMAN | SSSCA1 | 6.15E-02 | 3.98E-04 | 2.37E-02 |  | ['6.30E-08', '0.00E+00', '4.91E-08', '9.56E-08', '6.77E-08', '5.28E-08', '6.68E-08', '7.22E-08', '5.97E-08', '0.00E+00'] | ['0.00E+00', '0.00E+00', '0.00E+00', '0.00E+00', '0.00E+00', '0.00E+00', '0.00E+00', '0.00E+00', '0.00E+00', '0.00E+00'] | NA in controls | Upregulated |
| 12 | Q86V85\|GP180_HUMAN | GPR180 | 7.55E-02 | 8.20E-06 | 1.83E-03 |  | ['3.59E-08', '3.10E-08', '4.74E-08', '6.40E-08', '3.52E-08', '3.30E-08', '3.39E-08', '2.91E-08', '4.92E-08', '4.72E-08'] | ['0.00E+00', '0.00E+00', '2.58E-08', '0.00E+00', '0.00E+00', '0.00E+00', '0.00E+00', '0.00E+00', '0.00E+00', '2.34E-08'] | 8.3 | Upregulated |
| 13 | A4D1S5-2\|RAB19_HUMAN | RAB19 | 7.58E-02 | 5.73E-04 | 2.83E-02 |  | ['0.00E+00', '0.00E+00', '0.00E+00', '0.00E+00', '0.00E+00', '0.00E+00', '0.00E+00', '0.00E+00', '0.00E+00', '0.00E+00'] | ['8.01E-08', '1.04E-07', '0.00E+00', '1.47E-07', '1.45E-07', '0.00E+00', '8.97E-08', '1.58E-07', '8.98E-08', '1.59E-07'] | NA in IFN-treated | Downregulated |
| 14 | O75828\|CBR3_HUMAN | CBR3 | 8.23E-02 | 7.87E-04 | 3.36E-02 |  | ['9.80E-07', '8.65E-07', '8.66E-07', '9.69E-07', '7.15E-07', '6.70E-07', '0.00E+00', '8.97E-07', '6.73E-07', '5.19E-07'] | ['0.00E+00', '0.00E+00', '3.92E-07', '0.00E+00', '4.03E-07', '0.00E+00', '2.92E-07', '0.00E+00', '0.00E+00', '0.00E+00'] | 6.6 | Upregulated |
| 15 | Q9H8H3\|MET7A_HUMAN | METTL7A | 9.81E-02 | 7.56E-04 | 3.27E-02 |  | ['0.00E+00', '2.50E-07', '2.36E-07', '2.30E-07', '3.23E-07', '1.53E-07', '1.98E-07', '2.09E-07', '2.13E-07', '2.04E-07'] | ['8.42E-07', '7.62E-07', '0.00E+00', '6.63E-07', '9.49E-07', '6.10E-07', '5.55E-07', '8.17E-07', '5.20E-07', '8.19E-07'] | 0.308 | Downregulated |
| 16 | Q8NHP6-2\|MSPD2_HUMAN | MOSPD2 | 1.08E-01 | 1.24E-03 | 4.27E-02 |  | ['0.00E+00', '0.00E+00', '0.00E+00', '0.00E+00', '0.00E+00', '0.00E+00', '0.00E+00', '0.00E+00', '0.00E+00', '0.00E+00'] | ['1.22E-07', '1.02E-07', '1.08E-07', '2.40E-08', '0.00E+00', '0.00E+00', '9.92E-08', '5.39E-08', '1.12E-07', '6.83E-08'] | NA in IFN-treated | Downregulated |
| 17 | Q9BZL4-4\|PP12C_HUMAN | PPP1R12C | 1.13E-01 | 6.34E-04 | 3.00E-02 |  | ['1.96E-08', '3.47E-08', '9.50E-09', '1.90E-08', '3.50E-08', '0.00E+00', '0.00E+00', '1.13E-08', '2.18E-08', '3.15E-08'] | ['0.00E+00', '1.80E-08', '0.00E+00', '0.00E+00', '1.07E-08', '0.00E+00', '0.00E+00', '0.00E+00', '0.00E+00', '8.90E-09'] | 4.9 | Upregulated |
| 18 | P07602-2\|SAP_HUMAN | PSAP | 1.29E-01 | 5.14E-05 | 6.06E-03 |  | ['0.00E+00', '0.00E+00', '0.00E+00', '1.93E-06', '0.00E+00', '0.00E+00', '0.00E+00', '0.00E+00', '0.00E+00', '0.00E+00'] | ['2.76E-06', '2.09E-06', '0.00E+00', '3.21E-06', '3.12E-06', '2.93E-06', '2.44E-06', '3.23E-06', '1.99E-06', '2.35E-06'] | 0.08 | Downregulated |
| 19 | Q9NQP4\|PFD4_HUMAN | PFDN4 | 1.38E-01 | 1.95E-04 | 1.54E-02 |  | ['3.17E-07', '1.34E-07', '2.15E-07', '3.11E-07', '3.16E-07', '8.81E-08', '3.32E-07', '3.93E-07', '2.74E-07', '2.51E-07'] | ['0.00E+00', '7.58E-08', '7.89E-08', '0.00E+00', '7.33E-08', '8.35E-08', '6.88E-08', '8.76E-08', '8.09E-08', '0.00E+00'] | 4.8 | Upregulated |
| 20 | P22102\|PUR2_HUMAN | GART | 1.41E-01 | 2.41E-06 | 8.85E-04 |  | ['2.64E-07', '2.23E-07', '2.03E-07', '2.34E-07', '1.49E-07', '2.17E-07', '1.14E-07', '1.67E-07', '1.03E-07', '1.84E-07'] | ['1.19E-07', '5.84E-08', '7.37E-08', '8.67E-08', '5.85E-08', '6.98E-08', '4.75E-08', '4.48E-08', '3.54E-08', '1.58E-08'] | 3 | Upregulated |
| 21 | Q01995\|TAGL_HUMAN | TAGLN | 1.47E-01 | 1.22E-03 | 4.25E-02 |  | ['3.17E-07', '1.96E-07', '3.26E-07', '0.00E+00', '1.79E-07', '2.43E-07', '1.74E-07', '4.26E-07', '3.23E-07', '3.59E-07'] | ['0.00E+00', '0.00E+00', '0.00E+00', '1.04E-07', '0.00E+00', '1.34E-07', '7.10E-08', '1.39E-07', '9.78E-08', '8.30E-08'] | 4 | Upregulated |
| 22 | Q9C002\|NMES1_HUMAN | NMES1 | 1.49E-01 | 1.59E-04 | 1.38E-02 |  | ['0.00E+00', '6.85E-07', '6.51E-07', '8.09E-07', '7.81E-07', '6.50E-07', '2.02E-07', '7.55E-07', '6.86E-07', '6.74E-07'] | ['0.00E+00', '0.00E+00', '0.00E+00', '0.00E+00', '0.00E+00', '0.00E+00', '0.00E+00', '3.89E-07', '0.00E+00', '2.53E-07'] | 9.2 | Upregulated |
| 23 | Q9ULC4-3\|MCTS1_HUMAN | MCTS1 | 1.80E-01 | 2.41E-04 | 1.81E-02 |  | ['1.34E-07', '1.36E-07', '2.96E-07', '0.00E+00', '2.19E-07', '1.10E-07', '2.42E-07', '1.31E-07', '1.36E-07', '1.61E-07'] | ['0.00E+00', '0.00E+00', '0.00E+00', '0.00E+00', '1.03E-07', '0.00E+00', '0.00E+00', '0.00E+00', '0.00E+00', '0.00E+00'] | 15.2 | Upregulated |
| 24 | Q14142\|TRI14_HUMAN | TRIM14 | 1.88E-01 | 1.05E-04 | 1.08E-02 |  | ['8.59E-08', '1.18E-07', '2.46E-07', '3.87E-07', '2.03E-07', '3.33E-07', '2.91E-07', '1.25E-07', '2.87E-07', '1.60E-07'] | ['0.00E+00', '0.00E+00', '0.00E+00', '2.77E-08', '5.46E-08', '0.00E+00', '0.00E+00', '0.00E+00', '3.39E-08', '4.23E-08'] | 14.1 | Upregulated |
| 25 | Q9H3Q1\|BORG4_HUMAN | CDC42EP4 | 1.96E-01 | 4.86E-05 | 5.84E-03 |  | ['2.27E-08', '2.78E-08', '3.85E-08', '2.62E-08', '0.00E+00', '3.31E-08', '2.05E-08', '2.02E-08', '2.36E-08', '3.65E-08'] | ['0.00E+00', '0.00E+00', '0.00E+00', '0.00E+00', '0.00E+00', '0.00E+00', '0.00E+00', '0.00E+00', '0.00E+00', '0.00E+00'] | NA in controls | Upregulated |
| 26 | Q5U5X0\|LYRM7_HUMAN | LYRM7 | 2.17E-01 | 3.74E-04 | 2.33E-02 |  | ['0.00E+00', '1.61E-07', '0.00E+00', '1.92E-07', '1.63E-07', '0.00E+00', '1.54E-07', '1.62E-07', '1.73E-07', '1.48E-07'] | ['2.34E-07', '2.09E-07', '2.28E-07', '2.95E-07', '4.38E-07', '3.13E-07', '2.03E-07', '3.19E-07', '2.65E-07', '4.53E-07'] | 0.39 | Downregulated |
| 27 | P52630\|STAT2_HUMAN | STAT2 | 2.61E-01 | 4.98E-04 | 2.68E-02 |  | ['1.85E-08', '2.54E-08', '0.00E+00', '2.95E-08', '5.55E-08', '5.12E-08', '2.48E-08', '2.12E-08', '1.72E-08', '2.68E-08'] | ['0.00E+00', '0.00E+00', '0.00E+00', '0.00E+00', '0.00E+00', '0.00E+00', '0.00E+00', '0.00E+00', '0.00E+00', '0.00E+00'] | NA in controls | Upregulated |
| 28 | P09913\|IFIT2_HUMAN | IFIT2 | 2.62E-01 | 3.96E-05 | 5.49E-03 |  | ['7.51E-08', '2.92E-07', '1.59E-07', '2.63E-07', '9.25E-08', '1.27E-07', '1.23E-07', '2.41E-07', '2.56E-07', '2.30E-07'] | ['0.00E+00', '0.00E+00', '0.00E+00', '0.00E+00', '0.00E+00', '0.00E+00', '0.00E+00', '0.00E+00', '0.00E+00', '0.00E+00'] | NA in controls | Upregulated |
| 29 | P19525\|E2AK2_HUMAN | EIF2AK2 | 2.67E-01 | 1.51E-05 | 2.78E-03 |  | ['1.19E-07', '3.56E-07', '3.38E-07', '3.75E-07', '3.80E-07', '2.35E-07', '3.04E-07', '2.91E-07', '3.80E-07', '3.33E-07'] | ['8.21E-08', '0.00E+00', '1.00E-07', '8.64E-08', '0.00E+00', '2.33E-08', '9.21E-08', '2.87E-08', '1.45E-07', '2.52E-08'] | 5.3 | Upregulated |
| 30 | Q14240\|IF4A2_HUMAN | EIF4A2 | 2.69E-01 | 1.71E-04 | 1.41E-02 |  | ['2.36E-06', '2.14E-06', '2.69E-06', '2.35E-06', '1.72E-06', '1.55E-06', '2.33E-06', '2.11E-06', '1.96E-06', '1.88E-06'] | ['1.54E-06', '1.10E-06', '1.54E-06', '0.00E+00', '9.86E-07', '0.00E+00', '1.76E-06', '0.00E+00', '1.41E-06', '0.00E+00'] | 2.5 | Upregulated |
| 31 | P32455\|GBP1_HUMAN | GBP1 | 2.80E-01 | 6.52E-04 | 3.04E-02 |  | ['1.29E-07', '0.00E+00', '7.67E-08', '2.59E-07', '2.28E-07', '2.60E-07', '2.00E-07', '2.24E-07', '8.18E-08', '1.72E-07'] | ['0.00E+00', '4.41E-08', '0.00E+00', '6.11E-08', '0.00E+00', '3.77E-08', '0.00E+00', '6.27E-08', '0.00E+00', '6.46E-08'] | 6 | Upregulated |
| 32 | Q6PJT7\|ZC3HE_HUMAN | ZC3H14 | 2.88E-01 | 4.92E-04 | 2.68E-02 |  | ['1.29E-07', '6.37E-08', '2.01E-07', '1.06E-07', '8.65E-08', '7.92E-08', '7.22E-08', '9.57E-08', '1.31E-07', '1.75E-07'] | ['9.24E-08', '9.12E-08', '5.47E-08', '0.00E+00', '0.00E+00', '0.00E+00', '0.00E+00', '0.00E+00', '0.00E+00', '7.68E-08'] | 3.6 | Upregulated |
| 33 | Q92616\|GCN1_HUMAN | GCN1 | 2.88E-01 | 8.76E-04 | 3.47E-02 |  | ['4.35E-08', '5.18E-08', '6.47E-08', '4.49E-08', '3.33E-08', '2.67E-08', '7.92E-08', '2.10E-08', '4.93E-08', '4.89E-08'] | ['0.00E+00', '4.79E-09', '4.60E-08', '2.15E-08', '1.56E-08', '1.53E-08', '0.00E+00', '1.16E-08', '0.00E+00', '1.27E-08'] | 3.6 | Upregulated |
| 34 | Q9Y6G9\|DC1L1_HUMAN | DYNC1LI1 | 3.09E-01 | 1.07E-03 | 3.90E-02 |  | ['3.87E-08', '1.22E-08', '4.58E-08', '0.00E+00', '4.62E-08', '0.00E+00', '2.60E-08', '2.88E-08', '2.44E-08', '2.75E-08'] | ['0.00E+00', '0.00E+00', '0.00E+00', '0.00E+00', '0.00E+00', '0.00E+00', '0.00E+00', '0.00E+00', '0.00E+00', '0.00E+00'] | NA in controls | Upregulated |
| 35 | O95786\|DDX58_HUMAN | DDX58 | 3.20E-01 | 2.59E-05 | 4.15E-03 |  | ['1.60E-07', '1.77E-07', '3.29E-07', '3.60E-07', '2.25E-07', '2.35E-07', '1.24E-07', '1.98E-07', '1.07E-07', '1.52E-07'] | ['0.00E+00', '0.00E+00', '0.00E+00', '0.00E+00', '0.00E+00', '0.00E+00', '0.00E+00', '0.00E+00', '0.00E+00', '0.00E+00'] | NA in controls | Upregulated |
| 36 | Q13472-2\|TOP3A_HUMAN | TOP3A | 3.27E-01 | 1.21E-03 | 4.23E-02 |  | ['1.02E-08', '0.00E+00', '1.84E-08', '9.12E-09', '0.00E+00', '1.79E-08', '8.59E-09', '2.14E-08', '1.11E-08', '8.87E-09'] | ['0.00E+00', '0.00E+00', '0.00E+00', '0.00E+00', '0.00E+00', '0.00E+00', '0.00E+00', '0.00E+00', '0.00E+00', '0.00E+00'] | NA in controls | Upregulated |
| 37 | O00182-3\|LEG9_HUMAN | LGALS9 | 3.53E-01 | 1.35E-03 | 4.54E-02 |  | ['8.04E-08', '1.36E-07', '4.86E-07', '2.77E-07', '4.96E-07', '4.58E-07', '2.41E-07', '1.13E-07', '0.00E+00', '2.77E-07'] | ['0.00E+00', '0.00E+00', '0.00E+00', '0.00E+00', '0.00E+00', '0.00E+00', '0.00E+00', '0.00E+00', '0.00E+00', '0.00E+00'] | NA in controls | Upregulated |
| 38 | P05161\|ISG15_HUMAN | ISG15 | 3.53E-01 | 1.13E-06 | 5.90E-04 |  | ['5.00E-06', '5.43E-06', '3.89E-06', '3.17E-06', '5.04E-06', '3.04E-06', '4.03E-06', '2.47E-06', '4.80E-06', '2.78E-06'] | ['8.87E-08', '0.00E+00', '0.00E+00', '0.00E+00', '0.00E+00', '0.00E+00', '0.00E+00', '0.00E+00', '0.00E+00', '2.21E-07'] | 128 | Upregulated |
| 39 | O95864\|FADS2_HUMAN | FADS2 | 3.57E-01 | 1.18E-04 | 1.17E-02 |  | ['0.00E+00', '0.00E+00', '0.00E+00', '0.00E+00', '2.94E-07', '0.00E+00', '1.57E-07', '0.00E+00', '1.07E-07', '0.00E+00'] | ['4.56E-07', '3.97E-07', '5.37E-07', '2.24E-07', '2.58E-07', '3.20E-07', '5.18E-07', '2.67E-07', '5.74E-07', '2.94E-07'] | 0.145 | Downregulated |
| 40 | P13612\|ITA4_HUMAN | ITGA4 | 3.58E-01 | 7.96E-04 | 3.36E-02 |  | ['3.45E-08', '1.26E-08', '3.07E-08', '1.43E-08', '6.40E-09', '0.00E+00', '0.00E+00', '2.86E-08', '3.09E-08', '4.70E-08'] | ['5.73E-08', '6.94E-08', '1.09E-07', '9.86E-08', '2.53E-08', '8.91E-08', '6.02E-08', '6.78E-08', '3.59E-08', '6.76E-08'] | 0.301 | Downregulated |
| 41 | P21291\|CSRP1_HUMAN | CSRP1 | 3.59E-01 | 7.44E-05 | 8.45E-03 |  | ['1.14E-06', '7.52E-07', '1.24E-06', '8.87E-07', '1.04E-06', '6.60E-07', '9.73E-07', '6.30E-07', '1.30E-06', '1.19E-06'] | ['3.37E-07', '2.77E-07', '3.22E-07', '4.54E-07', '5.39E-07', '4.31E-07', '0.00E+00', '4.25E-07', '4.17E-07', '4.96E-07'] | 2.7 | Upregulated |
| 42 | P23497\|SP100_HUMAN | SP100 | 3.78E-01 | 5.14E-08 | 5.65E-05 |  | ['3.54E-07', '4.32E-07', '2.89E-07', '4.33E-07', '3.71E-07', '4.16E-07', '2.56E-07', '3.37E-07', '3.21E-07', '2.59E-07'] | ['0.00E+00', '0.00E+00', '0.00E+00', '0.00E+00', '0.00E+00', '0.00E+00', '0.00E+00', '0.00E+00', '0.00E+00', '0.00E+00'] | NA in controls | Upregulated |
| 43 | O75976\|CBPD_HUMAN | CPD | 3.86E-01 | 4.97E-04 | 2.68E-02 |  | ['0.00E+00', '0.00E+00', '1.32E-08', '4.43E-08', '0.00E+00', '1.13E-08', '2.78E-08', '0.00E+00', '1.30E-08', '0.00E+00'] | ['7.55E-08', '4.70E-08', '1.03E-07', '7.99E-08', '3.28E-08', '2.38E-08', '4.63E-08', '3.43E-08', '3.36E-08', '6.27E-08'] | 0.203 | Downregulated |
| 44 | P30622-2\|CLIP1_HUMAN | CLIP1 | 3.88E-01 | 1.15E-03 | 4.10E-02 |  | ['2.31E-08', '8.45E-08', '4.05E-08', '5.34E-08', '5.32E-08', '4.25E-08', '3.75E-08', '4.33E-08', '7.08E-08', '3.22E-08'] | ['1.22E-08', '9.31E-09', '0.00E+00', '2.77E-08', '2.79E-08', '5.69E-09', '1.22E-08', '1.84E-08', '2.81E-08', '3.41E-08'] | 2.7 | Upregulated |
| 45 | O00217\|NDUS8_HUMAN | NDUFS8 | 3.98E-01 | 4.05E-06 | 1.27E-03 |  | ['1.57E-07', '1.42E-07', '4.22E-07', '4.51E-07', '2.80E-07', '5.91E-07', '4.69E-07', '1.64E-07', '1.52E-07', '3.89E-07'] | ['6.59E-07', '4.30E-07', '1.14E-06', '7.69E-07', '7.50E-07', '1.14E-06', '9.11E-07', '8.60E-07', '4.63E-07', '1.03E-06'] | 0.395 | Downregulated |
| 46 | Q96C86\|DCPS_HUMAN | DCPS | 3.98E-01 | 8.35E-04 | 3.43E-02 |  | ['7.44E-08', '2.72E-07', '2.95E-07', '1.97E-07', '1.95E-07', '2.21E-07', '2.35E-07', '6.30E-08', '2.55E-07', '3.04E-07'] | ['0.00E+00', '1.86E-08', '0.00E+00', '1.53E-07', '1.39E-07', '3.72E-08', '0.00E+00', '3.10E-08', '8.11E-08', '1.79E-07'] | 3.3 | Upregulated |
| 47 | O43432-3\|IF4G3_HUMAN | EIF4G3 | 4.02E-01 | 3.88E-04 | 2.37E-02 |  | ['1.87E-08', '1.68E-08', '3.19E-08', '1.86E-08', '1.97E-08', '3.02E-08', '3.20E-08', '2.13E-08', '0.00E+00', '7.01E-09'] | ['0.00E+00', '7.50E-09', '0.00E+00', '0.00E+00', '0.00E+00', '0.00E+00', '0.00E+00', '0.00E+00', '0.00E+00', '0.00E+00'] | 26.2 | Upregulated |
| 48 | Q14258\|TRI25_HUMAN | TRIM25 | 4.03E-01 | 4.85E-05 | 5.84E-03 |  | ['2.16E-07', '9.03E-08', '1.62E-07', '8.66E-08', '2.41E-07', '1.20E-07', '1.79E-07', '1.72E-07', '1.59E-07', '2.46E-07'] | ['4.23E-08', '0.00E+00', '0.00E+00', '7.06E-08', '0.00E+00', '0.00E+00', '0.00E+00', '0.00E+00', '0.00E+00', '0.00E+00'] | 14.8 | Upregulated |
| 49 | O75525\|KHDR3_HUMAN | KHDRBS3 | 4.05E-01 | 3.28E-05 | 4.76E-03 |  | ['1.77E-07', '0.00E+00', '2.30E-07', '1.88E-07', '1.82E-07', '1.50E-07', '1.27E-07', '0.00E+00', '0.00E+00', '0.00E+00'] | ['4.78E-07', '5.34E-07', '3.45E-07', '4.31E-07', '3.54E-07', '3.47E-07', '4.84E-07', '2.64E-07', '3.01E-07', '2.80E-07'] | 0.276 | Downregulated |
| 50 | P40937-2\|RFC5_HUMAN | RFC5 | 4.21E-01 | 2.99E-04 | 2.01E-02 |  | ['0.00E+00', '4.84E-07', '3.09E-07', '6.78E-07', '4.24E-07', '1.80E-07', '3.54E-07', '2.80E-07', '4.18E-07', '4.71E-07'] | ['0.00E+00', '0.00E+00', '0.00E+00', '1.53E-07', '0.00E+00', '0.00E+00', '1.47E-07', '6.69E-08', '1.98E-07', '0.00E+00'] | 6.4 | Upregulated |
| 51 | P29728\|OAS2_HUMAN | OAS2 | 4.38E-01 | 7.63E-09 | 1.19E-05 |  | ['3.11E-07', '2.83E-07', '2.70E-07', '2.23E-07', '2.80E-07', '3.37E-07', '2.29E-07', '2.66E-07', '3.41E-07', '3.49E-07'] | ['0.00E+00', '0.00E+00', '0.00E+00', '0.00E+00', '0.00E+00', '0.00E+00', '0.00E+00', '0.00E+00', '0.00E+00', '0.00E+00'] | NA in controls | Upregulated |
| 52 | Q03519\|TAP2_HUMAN | TAP2 | 4.57E-01 | 4.07E-05 | 5.53E-03 |  | ['4.53E-07', '3.32E-07', '6.64E-07', '7.07E-07', '6.11E-07', '4.91E-07', '3.01E-07', '4.95E-07', '5.03E-07', '3.11E-07'] | ['2.11E-07', '1.98E-07', '0.00E+00', '2.21E-07', '2.36E-07', '1.76E-07', '0.00E+00', '2.59E-07', '1.18E-07', '0.00E+00'] | 3.4 | Upregulated |
| 53 | O43854\|EDIL3_HUMAN | EDIL3 | 4.70E-01 | 1.26E-05 | 2.46E-03 |  | ['3.30E-07', '3.35E-07', '2.91E-07', '3.41E-07', '2.75E-07', '3.07E-07', '3.73E-07', '3.94E-07', '2.28E-07', '4.75E-07'] | ['8.71E-07', '6.21E-07', '7.49E-07', '1.04E-06', '1.08E-06', '1.14E-06', '7.27E-07', '1.02E-06', '5.51E-07', '9.14E-07'] | 0.384 | Downregulated |
| 54 | Q9NQW6\|ANLN_HUMAN | ANLN | 4.72E-01 | 6.86E-06 | 1.71E-03 |  | ['1.34E-07', '1.47E-07', '6.93E-08', '1.78E-07', '1.48E-07', '1.64E-07', '1.12E-07', '1.74E-07', '1.25E-07', '1.62E-07'] | ['2.98E-08', '4.97E-08', '1.46E-08', '3.91E-08', '5.24E-08', '4.03E-08', '8.08E-08', '4.64E-08', '3.98E-08', '5.64E-08'] | 3.1 | Upregulated |
| 55 | P17931\|LEG3_HUMAN | LGALS3 | 4.95E-01 | 5.70E-07 | 3.96E-04 |  | ['2.30E-06', '1.94E-06', '1.80E-06', '2.02E-06', '2.07E-06', '2.45E-06', '2.06E-06', '2.24E-06', '1.74E-06', '2.02E-06'] | ['7.81E-07', '9.32E-07', '7.35E-07', '1.15E-07', '1.24E-06', '1.03E-06', '6.22E-07', '1.01E-06', '7.23E-07', '9.26E-07'] | 2.5 | Upregulated |
| 56 | Q14517\|FAT1_HUMAN | FAT1 | 5.02E-01 | 1.16E-03 | 4.10E-02 |  | ['0.00E+00', '0.00E+00', '2.87E-09', '0.00E+00', '0.00E+00', '0.00E+00', '0.00E+00', '0.00E+00', '6.97E-09', '2.55E-09'] | ['3.92E-09', '8.42E-09', '1.36E-08', '1.90E-08', '9.36E-09', '4.86E-09', '9.17E-09', '7.74E-09', '5.89E-09', '2.09E-08'] | 0.12 | Downregulated |
| 57 | Q63HN8\|RN213_HUMAN | RNF213 | 5.17E-01 | 5.39E-06 | 1.53E-03 |  | ['3.62E-08', '5.17E-08', '4.00E-08', '7.21E-08', '6.60E-08', '8.66E-08', '5.57E-08', '4.97E-08', '3.19E-08', '3.89E-08'] | ['0.00E+00', '0.00E+00', '0.00E+00', '0.00E+00', '0.00E+00', '0.00E+00', '0.00E+00', '0.00E+00', '0.00E+00', '0.00E+00'] | NA in controls | Upregulated |
| 58 | P28838\|AMPL_HUMAN | LAP3 | 5.27E-01 | 1.36E-06 | 6.56E-04 |  | ['1.06E-06', '8.00E-07', '1.22E-06', '1.04E-06', '1.26E-06', '9.28E-07', '1.35E-06', '1.28E-06', '1.18E-06', '9.92E-07'] | ['3.42E-07', '4.94E-07', '4.15E-07', '3.57E-07', '1.76E-07', '2.94E-07', '3.51E-07', '3.09E-07', '3.04E-07', '1.35E-07'] | 3.5 | Upregulated |
| 59 | P13645\|K1C10_HUMAN | KRT10 | 5.31E-01 | 9.26E-05 | 1.01E-02 |  | ['0.00E+00', '0.00E+00', '1.79E-07', '3.54E-07', '1.25E-07', '1.36E-07', '1.20E-07', '1.13E-07', '0.00E+00', '1.57E-07'] | ['4.15E-07', '2.61E-07', '3.12E-07', '6.72E-07', '6.84E-07', '6.43E-07', '4.86E-07', '7.43E-07', '1.15E-07', '7.59E-07'] | 0.233 | Downregulated |
| 60 | Q8IYM9-2\|TRI22_HUMAN | TRIM22 | 5.31E-01 | 4.72E-04 | 2.61E-02 |  | ['2.87E-07', '1.34E-07', '4.38E-07', '1.51E-07', '2.37E-07', '1.36E-07', '1.92E-07', '2.60E-07', '2.20E-07', '1.92E-07'] | ['0.00E+00', '0.00E+00', '0.00E+00', '9.59E-08', '5.73E-08', '1.08E-07', '0.00E+00', '3.35E-08', '0.00E+00', '0.00E+00'] | 7.6 | Upregulated |
| 61 | O96019\|ACL6A_HUMAN | ACTL6A | 5.35E-01 | 1.05E-03 | 3.84E-02 |  | ['2.50E-07', '2.69E-07', '2.25E-07', '0.00E+00', '2.55E-07', '1.34E-07', '1.89E-07', '2.13E-07', '1.88E-07', '9.24E-08'] | ['1.78E-07', '0.00E+00', '0.00E+00', '0.00E+00', '0.00E+00', '0.00E+00', '0.00E+00', '1.95E-07', '0.00E+00', '0.00E+00'] | 4.9 | Upregulated |
| 62 | P11586\|C1TC_HUMAN | MTHFD1 | 5.51E-01 | 5.30E-04 | 2.76E-02 |  | ['3.21E-07', '1.69E-07', '2.67E-07', '1.99E-07', '3.10E-07', '2.15E-07', '2.42E-07', '1.78E-07', '1.50E-07', '2.10E-07'] | ['5.22E-08', '1.68E-07', '4.62E-08', '5.62E-08', '8.87E-08', '9.07E-08', '5.07E-08', '6.51E-08', '1.34E-07', '6.84E-08'] | 2.8 | Upregulated |
| 63 | P26639\|SYTC_HUMAN | TARS | 5.54E-01 | 5.40E-04 | 2.79E-02 |  | ['1.93E-07', '1.13E-07', '2.03E-07', '1.83E-07', '1.93E-07', '9.53E-08', '1.14E-07', '1.34E-07', '2.05E-07', '1.65E-07'] | ['1.03E-07', '9.82E-08', '7.81E-08', '5.69E-08', '0.00E+00', '6.70E-08', '7.93E-08', '4.04E-08', '2.73E-08', '2.01E-08'] | 2.8 | Upregulated |
| 64 | P23921\|RIR1_HUMAN | RRM1 | 5.81E-01 | 1.37E-03 | 4.55E-02 |  | ['2.82E-08', '1.61E-08', '6.74E-08', '7.39E-08', '4.29E-08', '4.39E-08', '3.43E-08', '6.55E-08', '8.17E-08', '0.00E+00'] | ['0.00E+00', '0.00E+00', '2.55E-08', '0.00E+00', '1.75E-08', '0.00E+00', '2.43E-08', '1.95E-08', '8.25E-09', '0.00E+00'] | 4.8 | Upregulated |
| 65 | Q9GZZ1\|NAA50_HUMAN | NAA50 | 5.82E-01 | 8.45E-04 | 3.45E-02 |  | ['0.00E+00', '1.50E-07', '1.10E-07', '2.87E-07', '3.23E-07', '1.06E-07', '1.81E-07', '2.45E-07', '2.42E-07', '0.00E+00'] | ['0.00E+00', '0.00E+00', '0.00E+00', '1.62E-07', '1.74E-07', '2.89E-08', '0.00E+00', '5.15E-08', '0.00E+00', '0.00E+00'] | 3.9 | Upregulated |
| 66 | Q9BVC6\|TM109_HUMAN | TMEM109 | 5.88E-01 | 1.20E-03 | 4.20E-02 |  | ['2.41E-07', '0.00E+00', '5.77E-07', '4.40E-07', '9.20E-07', '3.58E-07', '4.98E-07', '1.42E-07', '1.78E-07', '2.47E-07'] | ['8.99E-07', '7.43E-07', '7.47E-07', '1.60E-06', '7.52E-07', '1.05E-06', '7.42E-07', '7.50E-07', '8.80E-07', '1.21E-06'] | 0.384 | Downregulated |
| 67 | P60891\|PRPS1_HUMAN | PRPS1 | 5.98E-01 | 9.06E-06 | 1.94E-03 |  | ['2.57E-07', '2.86E-07', '4.31E-07', '2.04E-07', '3.44E-07', '4.75E-07', '3.71E-07', '2.81E-07', '3.22E-07', '2.04E-07'] | ['0.00E+00', '0.00E+00', '2.16E-07', '8.26E-08', '2.34E-07', '1.26E-07', '0.00E+00', '0.00E+00', '0.00E+00', '0.00E+00'] | 4.8 | Upregulated |
| 68 | Q9Y508\|RN114_HUMAN | RNF114 | 5.99E-01 | 1.01E-04 | 1.05E-02 |  | ['1.92E-07', '1.73E-07', '2.30E-07', '3.96E-07', '1.75E-07', '2.75E-07', '1.40E-07', '2.05E-07', '1.65E-07', '7.41E-08'] | ['0.00E+00', '0.00E+00', '0.00E+00', '3.09E-08', '0.00E+00', '0.00E+00', '0.00E+00', '1.38E-07', '0.00E+00', '0.00E+00'] | 12 | Upregulated |
| 69 | P09914\|IFIT1_HUMAN | IFIT1 | 6.02E-01 | 1.02E-06 | 5.78E-04 |  | ['7.85E-07', '3.93E-07', '6.13E-07', '1.04E-06', '8.07E-07', '9.78E-07', '9.98E-07', '7.54E-07', '5.32E-07', '7.78E-07'] | ['0.00E+00', '0.00E+00', '0.00E+00', '0.00E+00', '0.00E+00', '0.00E+00', '0.00E+00', '0.00E+00', '0.00E+00', '0.00E+00'] | NA in controls | Upregulated |
| 70 | P35908\|K22E_HUMAN | KRT2 | 6.36E-01 | 9.15E-05 | 1.01E-02 |  | ['0.00E+00', '0.00E+00', '0.00E+00', '0.00E+00', '0.00E+00', '0.00E+00', '0.00E+00', '0.00E+00', '0.00E+00', '0.00E+00'] | ['0.00E+00', '2.87E-07', '4.76E-07', '4.23E-07', '4.47E-07', '5.90E-07', '3.19E-07', '3.58E-07', '3.14E-07', '6.71E-07'] | NA in IFN-treated | Downregulated |
| 71 | Q9BYK8\|HELZ2_HUMAN | HELZ2 | 6.45E-01 | 1.37E-04 | 1.27E-02 |  | ['6.23E-08', '6.96E-08', '8.77E-08', '9.37E-08', '0.00E+00', '4.30E-08', '5.41E-08', '9.70E-08', '4.99E-08', '3.67E-08'] | ['0.00E+00', '0.00E+00', '0.00E+00', '0.00E+00', '0.00E+00', '0.00E+00', '0.00E+00', '0.00E+00', '0.00E+00', '0.00E+00'] | NA in controls | Upregulated |
| 72 | P53004\|BIEA_HUMAN | BLVRA | 6.47E-01 | 7.56E-04 | 3.27E-02 |  | ['3.73E-07', '2.34E-07', '3.82E-07', '1.95E-07', '1.76E-07', '1.40E-07', '3.03E-07', '3.56E-07', '9.86E-08', '1.51E-07'] | ['5.30E-08', '3.51E-08', '0.00E+00', '8.62E-08', '2.82E-08', '5.99E-08', '0.00E+00', '7.45E-08', '0.00E+00', '1.39E-07'] | 5.1 | Upregulated |
| 73 | P23381-2\|SYWC_HUMAN | WARS | 6.73E-01 | 3.55E-06 | 1.17E-03 |  | ['7.54E-07', '8.04E-07', '1.32E-06', '8.54E-07', '7.85E-07', '8.72E-07', '7.38E-07', '5.80E-07', '5.22E-07', '6.54E-07'] | ['1.83E-07', '1.71E-07', '2.64E-07', '1.50E-07', '0.00E+00', '1.88E-07', '2.38E-07', '0.00E+00', '2.25E-07', '1.35E-07'] | 5.1 | Upregulated |
| 74 | Q9BQE5\|APOL2_HUMAN | APOL2 | 6.76E-01 | 1.48E-05 | 2.78E-03 |  | ['9.53E-07', '1.13E-06', '9.04E-07', '6.86E-07', '8.64E-07', '9.43E-07', '6.32E-07', '6.31E-07', '9.77E-07', '8.66E-07'] | ['2.44E-07', '1.82E-07', '6.71E-08', '3.93E-07', '4.31E-08', '3.28E-07', '1.55E-07', '3.45E-07', '4.85E-07', '3.23E-07'] | 3.3 | Upregulated |
| 75 | Q99575\|POP1_HUMAN | POP1 | 6.87E-01 | 8.68E-04 | 3.47E-02 |  | ['2.64E-08', '1.40E-08', '4.65E-08', '5.78E-08', '4.39E-08', '3.04E-08', '0.00E+00', '2.51E-08', '3.62E-08', '5.49E-08'] | ['8.61E-09', '9.69E-09', '0.00E+00', '4.04E-08', '0.00E+00', '1.02E-08', '0.00E+00', '0.00E+00', '0.00E+00', '2.97E-08'] | 3.4 | Upregulated |
| 76 | Q5EBM0-3\|CMPK2_HUMAN | CMPK2 | 6.90E-01 | 1.06E-03 | 3.86E-02 |  | ['1.52E-07', '1.35E-07', '5.84E-08', '8.05E-08', '9.84E-08', '1.98E-07', '1.63E-07', '0.00E+00', '1.11E-07', '0.00E+00'] | ['0.00E+00', '0.00E+00', '0.00E+00', '0.00E+00', '0.00E+00', '0.00E+00', '0.00E+00', '0.00E+00', '0.00E+00', '0.00E+00'] | NA in controls | Upregulated |
| 77 | P09429\|HMGB1_HUMAN | HMGB1 | 6.93E-01 | 9.32E-06 | 1.94E-03 |  | ['2.30E-06', '2.74E-06', '1.34E-06', '2.55E-06', '1.75E-06', '1.96E-06', '2.65E-06', '2.45E-06', '2.00E-06', '2.71E-06'] | ['2.16E-07', '8.91E-07', '5.93E-07', '9.12E-07', '9.22E-07', '4.50E-07', '1.75E-07', '7.90E-07', '9.75E-07', '1.12E-06'] | 3.2 | Upregulated |
| 78 | P20592\|MX2_HUMAN | MX2 | 6.97E-01 | 6.31E-11 | 1.97E-07 |  | ['2.07E-06', '2.20E-06', '2.00E-06', '2.30E-06', '1.81E-06', '2.11E-06', '1.71E-06', '2.11E-06', '1.87E-06', '2.13E-06'] | ['0.00E+00', '0.00E+00', '0.00E+00', '0.00E+00', '0.00E+00', '0.00E+00', '0.00E+00', '0.00E+00', '0.00E+00', '0.00E+00'] | NA in controls | Upregulated |
| 79 | O60701\|UGDH_HUMAN | UGDH | 6.98E-01 | 6.19E-06 | 1.68E-03 |  | ['2.83E-07', '3.08E-07', '3.54E-07', '3.78E-07', '4.15E-07', '2.73E-07', '2.88E-07', '3.24E-07', '3.41E-07', '3.33E-07'] | ['6.03E-08', '1.12E-07', '3.39E-08', '1.99E-07', '1.68E-07', '1.21E-07', '8.37E-08', '1.86E-07', '2.89E-08', '2.26E-07'] | 2.7 | Upregulated |
| 80 | Q8NC51-2\|PAIRB_HUMAN | SERBP1 | 7.14E-01 | 1.31E-04 | 1.26E-02 |  | ['9.22E-07', '9.66E-07', '6.96E-07', '7.17E-07', '1.35E-06', '1.07E-06', '1.45E-06', '1.07E-06', '1.37E-06', '1.26E-06'] | ['7.00E-07', '0.00E+00', '0.00E+00', '0.00E+00', '7.51E-07', '7.27E-07', '0.00E+00', '0.00E+00', '7.35E-07', '7.26E-07'] | 3 | Upregulated |
| 81 | P17858\|PFKAL_HUMAN | PFKL | 7.22E-01 | 5.75E-04 | 2.83E-02 |  | ['8.88E-08', '1.05E-07', '1.80E-07', '1.38E-07', '1.76E-07', '1.13E-07', '1.20E-07', '9.95E-08', '7.03E-08', '9.41E-08'] | ['5.17E-08', '0.00E+00', '0.00E+00', '2.99E-08', '9.04E-08', '9.92E-08', '0.00E+00', '4.83E-08', '2.77E-08', '3.09E-08'] | 3.1 | Upregulated |
| 82 | Q969M3\|YIPF5_HUMAN | YIPF5 | 7.47E-01 | 6.86E-04 | 3.13E-02 |  | ['6.29E-08', '0.00E+00', '1.20E-07', '0.00E+00', '0.00E+00', '4.28E-08', '0.00E+00', '7.37E-08', '0.00E+00', '5.86E-08'] | ['9.02E-08', '1.86E-07', '9.87E-08', '2.28E-07', '2.80E-07', '1.54E-07', '1.85E-07', '3.14E-07', '1.48E-07', '1.83E-07'] | 0.192 | Downregulated |
| 83 | Q9H6Z4-3\|RANB3_HUMAN | RANBP3 | 7.52E-01 | 5.47E-04 | 2.80E-02 |  | ['5.63E-08', '1.59E-07', '9.89E-08', '1.45E-07', '2.53E-07', '1.14E-07', '2.43E-07', '6.10E-08', '1.54E-08', '1.12E-07'] | ['0.00E+00', '0.00E+00', '0.00E+00', '3.72E-08', '8.20E-08', '0.00E+00', '0.00E+00', '0.00E+00', '0.00E+00', '4.11E-08'] | 7.8 | Upregulated |
| 84 | Q96KP4\|CNDP2_HUMAN | CNDP2 | 7.64E-01 | 1.65E-05 | 2.90E-03 |  | ['6.61E-07', '5.38E-07', '5.33E-07', '4.50E-07', '3.76E-07', '2.61E-07', '5.69E-07', '4.97E-07', '4.25E-07', '2.15E-07'] | ['8.99E-08', '1.53E-07', '8.69E-08', '3.64E-08', '8.03E-08', '1.02E-07', '3.88E-08', '1.94E-07', '1.13E-07', '3.94E-08'] | 4.8 | Upregulated |
| 85 | Q13287\|NMI_HUMAN | NMI | 7.69E-01 | 5.43E-08 | 5.65E-05 |  | ['2.12E-07', '2.45E-07', '1.78E-07', '2.38E-07', '2.90E-07', '2.76E-07', '1.73E-07', '1.60E-07', '2.19E-07', '2.31E-07'] | ['0.00E+00', '0.00E+00', '0.00E+00', '0.00E+00', '0.00E+00', '0.00E+00', '0.00E+00', '0.00E+00', '0.00E+00', '0.00E+00'] | NA in controls | Upregulated |
| 86 | O14879\|IFIT3_HUMAN | IFIT3 | 7.84E-01 | 2.60E-09 | 5.41E-06 |  | ['5.74E-07', '6.83E-07', '4.55E-07', '6.61E-07', '7.54E-07', '6.90E-07', '5.95E-07', '6.68E-07', '5.57E-07', '5.88E-07'] | ['0.00E+00', '0.00E+00', '0.00E+00', '0.00E+00', '0.00E+00', '0.00E+00', '0.00E+00', '0.00E+00', '0.00E+00', '0.00E+00'] | NA in controls | Upregulated |
| 87 | Q8TDB6\|DTX3L_HUMAN | DTX3L | 7.89E-01 | 2.15E-06 | 8.39E-04 |  | ['1.03E-07', '1.30E-07', '1.94E-07', '1.74E-07', '2.44E-07', '2.30E-07', '1.55E-07', '1.28E-07', '1.78E-07', '1.17E-07'] | ['8.63E-09', '0.00E+00', '0.00E+00', '0.00E+00', '0.00E+00', '0.00E+00', '0.00E+00', '0.00E+00', '0.00E+00', '0.00E+00'] | 191.5 | Upregulated |
| 88 | O15347\|HMGB3_HUMAN | HMGB3 | 7.97E-01 | 2.39E-04 | 1.81E-02 |  | ['2.33E-07', '3.05E-07', '6.19E-07', '5.74E-07', '2.49E-07', '5.09E-07', '4.40E-07', '2.27E-07', '2.91E-07', '3.60E-07'] | ['0.00E+00', '0.00E+00', '1.88E-07', '0.00E+00', '0.00E+00', '0.00E+00', '0.00E+00', '1.99E-07', '1.03E-07', '1.70E-07'] | 5.8 | Upregulated |
| 89 | Q5K651\|SAMD9_HUMAN | SAMD9 | 8.08E-01 | 2.47E-05 | 4.07E-03 |  | ['8.47E-08', '4.99E-08', '8.30E-08', '7.25E-08', '1.09E-07', '1.14E-07', '5.47E-08', '5.01E-08', '2.31E-08', '6.20E-08'] | ['0.00E+00', '0.00E+00', '0.00E+00', '0.00E+00', '0.00E+00', '0.00E+00', '0.00E+00', '0.00E+00', '0.00E+00', '0.00E+00'] | NA in controls | Upregulated |
| 90 | P29279\|CTGF_HUMAN | CTGF | 8.32E-01 | 1.59E-04 | 1.38E-02 |  | ['0.00E+00', '0.00E+00', '0.00E+00', '0.00E+00', '0.00E+00', '0.00E+00', '0.00E+00', '0.00E+00', '0.00E+00', '0.00E+00'] | ['1.13E-07', '2.04E-07', '1.09E-07', '1.91E-07', '1.65E-07', '1.74E-07', '2.82E-07', '1.17E-07', '2.64E-08', '3.23E-07'] | NA in IFN-treated | Downregulated |
| 91 | P20962\|PTMS_HUMAN | PTMS | 8.39E-01 | 1.21E-04 | 1.18E-02 |  | ['7.89E-07', '9.28E-07', '1.03E-06', '1.18E-06', '2.08E-06', '1.78E-06', '8.27E-07', '9.84E-07', '1.64E-06', '1.63E-06'] | ['2.28E-07', '0.00E+00', '0.00E+00', '0.00E+00', '3.20E-07', '0.00E+00', '1.06E-07', '7.85E-07', '2.44E-07', '8.46E-07'] | 5.1 | Upregulated |
| 92 | P30511-3\|HLAF_HUMAN | HLA-F | 8.44E-01 | 3.23E-06 | 1.12E-03 |  | ['6.76E-08', '7.71E-08', '3.93E-08', '4.48E-08', '5.69E-08', '9.70E-08', '5.95E-08', '8.28E-08', '3.63E-08', '7.43E-08'] | ['0.00E+00', '0.00E+00', '0.00E+00', '0.00E+00', '0.00E+00', '0.00E+00', '0.00E+00', '0.00E+00', '0.00E+00', '0.00E+00'] | NA in controls | Upregulated |
| 93 | Q06323\|PSME1_HUMAN | PSME1 | 8.48E-01 | 1.54E-06 | 6.88E-04 |  | ['5.74E-07', '5.60E-07', '6.81E-07', '6.73E-07', '9.92E-07', '8.43E-07', '5.52E-07', '6.62E-07', '5.36E-07', '5.14E-07'] | ['9.10E-08', '1.07E-07', '3.57E-07', '0.00E+00', '6.26E-07', '4.63E-07', '1.07E-07', '1.24E-07', '1.90E-07', '2.69E-07'] | 2.8 | Upregulated |
| 94 | Q9Y3Z3-2\|SAMH1_HUMAN | SAMHD1 | 8.55E-01 | 1.52E-04 | 1.37E-02 |  | ['8.28E-07', '3.97E-07', '1.16E-06', '1.56E-06', '0.00E+00', '1.12E-06', '1.04E-06', '6.38E-07', '9.62E-07', '8.55E-07'] | ['0.00E+00', '0.00E+00', '0.00E+00', '0.00E+00', '0.00E+00', '0.00E+00', '0.00E+00', '0.00E+00', '0.00E+00', '0.00E+00'] | NA in controls | Upregulated |
| 95 | Q9NZT2-2\|OGFR_HUMAN | OGFR | 8.70E-01 | 1.48E-04 | 1.36E-02 |  | ['5.50E-08', '8.10E-08', '1.92E-07', '1.29E-07', '2.05E-07', '1.39E-07', '1.23E-07', '1.18E-07', '7.29E-08', '1.22E-07'] | ['2.56E-08', '2.34E-08', '5.73E-08', '4.16E-08', '4.04E-08', '2.24E-08', '2.93E-08', '1.52E-08', '5.62E-08', '2.59E-08'] | 3.7 | Upregulated |
| 96 | P30085\|KCY_HUMAN | CMPK1 | 8.85E-01 | 1.14E-03 | 4.10E-02 |  | ['3.09E-07', '2.09E-07', '3.99E-07', '3.78E-07', '4.98E-07', '3.45E-07', '4.65E-07', '5.43E-07', '3.37E-07', '0.00E+00'] | ['1.71E-07', '4.41E-08', '2.18E-07', '3.15E-07', '0.00E+00', '0.00E+00', '1.62E-07', '2.78E-07', '1.57E-07', '0.00E+00'] | 2.6 | Upregulated |
| 97 | P62807\|H2B1C_HUMAN | HIST1H2BC | 8.92E-01 | 1.66E-04 | 1.40E-02 |  | ['2.80E-05', '1.82E-05', '0.00E+00', '1.67E-05', '0.00E+00', '0.00E+00', '0.00E+00', '0.00E+00', '1.49E-05', '0.00E+00'] | ['6.78E-05', '1.91E-05', '2.54E-05', '2.52E-05', '2.24E-05', '3.04E-05', '1.96E-05', '1.80E-05', '4.60E-05', '2.25E-05'] | 0.262 | Downregulated |
| 98 | Q8NCW5\|NNRE_HUMAN | NAXE | 9.06E-01 | 1.41E-03 | 4.65E-02 |  | ['1.84E-07', '2.02E-07', '7.96E-08', '0.00E+00', '4.08E-08', '1.09E-07', '1.11E-07', '1.55E-07', '2.57E-07', '1.52E-07'] | ['0.00E+00', '0.00E+00', '0.00E+00', '0.00E+00', '0.00E+00', '0.00E+00', '0.00E+00', '0.00E+00', '0.00E+00', '1.16E-07'] | 11.1 | Upregulated |
| 99 | P61916-2\|NPC2_HUMAN | NPC2 | 9.17E-01 | 3.79E-04 | 2.35E-02 |  | ['2.86E-06', '2.60E-06', '2.76E-06', '0.00E+00', '2.03E-06', '1.73E-06', '2.43E-06', '3.37E-06', '3.20E-06', '1.82E-06'] | ['1.42E-06', '1.80E-06', '0.00E+00', '0.00E+00', '0.00E+00', '0.00E+00', '0.00E+00', '2.45E-06', '2.40E-06', '0.00E+00'] | 2.8 | Upregulated |
| 100 | P20591\|MX1_HUMAN | MX1 | 9.50E-01 | 1.88E-11 | 1.18E-07 |  | ['2.94E-06', '3.06E-06', '2.96E-06', '3.29E-06', '3.51E-06', '3.23E-06', '2.67E-06', '3.36E-06', '3.11E-06', '2.95E-06'] | ['0.00E+00', '0.00E+00', '0.00E+00', '0.00E+00', '0.00E+00', '0.00E+00', '0.00E+00', '0.00E+00', '0.00E+00', '0.00E+00'] | NA in controls | Upregulated |
| 101 | Q9BW19\|KIFC1_HUMAN | KIFC1 | 9.70E-01 | 1.59E-03 | 4.99E-02 |  | ['2.76E-08', '4.64E-08', '1.60E-07', '8.58E-08', '6.49E-08', '7.32E-08', '1.25E-07', '5.90E-08', '4.17E-08', '9.88E-08'] | ['3.43E-08', '2.84E-08', '1.47E-08', '0.00E+00', '1.13E-08', '3.29E-08', '9.69E-09', '0.00E+00', '0.00E+00', '1.06E-08'] | 5.5 | Upregulated |
| 102 | Q9NUQ6\|SPS2L_HUMAN | SPATS2L | 9.81E-01 | 4.73E-05 | 5.84E-03 |  | ['7.44E-08', '1.84E-07', '2.55E-07', '2.24E-07', '2.60E-07', '3.40E-07', '3.23E-07', '1.74E-07', '1.92E-07', '2.36E-07'] | ['1.47E-08', '0.00E+00', '0.00E+00', '7.19E-08', '1.93E-08', '0.00E+00', '9.60E-08', '5.42E-08', '8.47E-08', '2.43E-08'] | 6.2 | Upregulated |
| 103 | P42224\|STAT1_HUMAN | STAT1 | 9.94E-01 | 1.84E-07 | 1.65E-04 |  | ['2.72E-07', '5.38E-07', '4.09E-07', '6.03E-07', '4.54E-07', '5.01E-07', '4.32E-07', '5.31E-07', '3.43E-07', '3.86E-07'] | ['0.00E+00', '0.00E+00', '0.00E+00', '0.00E+00', '0.00E+00', '0.00E+00', '0.00E+00', '0.00E+00', '0.00E+00', '0.00E+00'] | NA in controls | Upregulated |
| **EXTRA proteins identified using *paired t-test (ND) + Kruskal-Wallis test (NND) / No Imputation* (Workflow B)** | | | | | | | | | | |
| 1 | P60673\|PROF3_HUMAN | PFN3 | 5.58E-02 | 2.36E-03 | 3.72E-02 |  | ['7.79E-07', '5.73E-07', '0.00E+00', '0.00E+00', '0.00E+00', '4.52E-07', '4.14E-07', '7.09E-07', '8.48E-07', '7.78E-07'] | ['0.00E+00', '0.00E+00', '0.00E+00', '0.00E+00', '0.00E+00', '0.00E+00', '0.00E+00', '0.00E+00', '0.00E+00', '0.00E+00'] | NA in controls | Upregulated |
| 2 | Q92930\|RAB8B_HUMAN | RAB8B | 5.85E-02 | 2.11E-03 | 3.42E-02 |  | ['0.00E+00', '0.00E+00', '2.17E-06', '1.97E-06', '0.00E+00', '2.44E-06', '1.70E-06', '2.57E-06', '2.81E-06', '1.38E-06'] | ['0.00E+00', '0.00E+00', '0.00E+00', '0.00E+00', '0.00E+00', '0.00E+00', '0.00E+00', '0.00E+00', '0.00E+00', '0.00E+00'] | NA in controls | Upregulated |
| 3 | Q92785-2\|REQU_HUMAN | DPF2 | 7.20E-02 | 2.84E-03 | 4.07E-02 |  | ['0.00E+00', '0.00E+00', '1.06E-07', '1.11E-07', '0.00E+00', '1.09E-07', '7.37E-08', '6.04E-08', '5.61E-08', '5.91E-08'] | ['0.00E+00', '0.00E+00', '0.00E+00', '0.00E+00', '0.00E+00', '0.00E+00', '0.00E+00', '0.00E+00', '0.00E+00', '0.00E+00'] | NA in controls | Upregulated |
| 4 | P48668\|K2C6C_HUMAN | KRT6C | 7.35E-02 | 3.52E-03 | 4.53E-02 |  | ['0.00E+00', '0.00E+00', '0.00E+00', '0.00E+00', '0.00E+00', '0.00E+00', '0.00E+00', '0.00E+00', '0.00E+00', '0.00E+00'] | ['0.00E+00', '0.00E+00', '0.00E+00', '1.99E-06', '2.06E-06', '2.25E-06', '8.23E-07', '1.26E-06', '9.91E-07', '2.01E-06'] | NA in IFN-treated | Downregulated |
| 5 | Q9NPA8-2\|ENY2_HUMAN | ENY2 | 7.89E-02 | 2.78E-03 | 4.04E-02 |  | ['0.00E+00', '9.83E-07', '4.25E-07', '1.17E-06', '1.14E-06', '0.00E+00', '8.54E-07', '5.56E-07', '0.00E+00', '7.81E-07'] | ['0.00E+00', '2.60E-07', '0.00E+00', '4.13E-07', '7.91E-07', '0.00E+00', '0.00E+00', '0.00E+00', '0.00E+00', '0.00E+00'] | 4 | Upregulated |
| 6 | Q9H2H8-2\|PPIL3_HUMAN | PPIL3 | 8.78E-02 | 2.45E-03 | 3.76E-02 |  | ['1.42E-07', '0.00E+00', '1.56E-07', '1.08E-07', '0.00E+00', '0.00E+00', '1.11E-07', '6.76E-08', '1.34E-07', '1.51E-07'] | ['4.99E-08', '0.00E+00', '0.00E+00', '0.00E+00', '0.00E+00', '0.00E+00', '0.00E+00', '0.00E+00', '0.00E+00', '0.00E+00'] | 17.4 | Upregulated |
| 7 | P30443\|1A01_HUMAN | HLA-A | 8.94E-02 | 1.76E-03 | 3.06E-02 |  | ['1.76E-06', '2.27E-06', '4.86E-07', '1.58E-06', '3.06E-07', '1.80E-06', '1.60E-06', '1.85E-06', '2.89E-07', '4.34E-07'] | ['0.00E+00', '1.46E-06', '0.00E+00', '2.84E-07', '0.00E+00', '0.00E+00', '0.00E+00', '1.34E-06', '0.00E+00', '2.77E-07'] | 3.7 | Upregulated |
| 8 | P28482\|MK01_HUMAN | MAPK1 | 9.86E-02 | 3.44E-03 | 4.51E-02 |  | ['0.00E+00', '0.00E+00', '3.71E-07', '3.26E-07', '3.58E-07', '3.41E-07', '1.84E-07', '2.02E-07', '0.00E+00', '1.98E-07'] | ['0.00E+00', '0.00E+00', '0.00E+00', '1.62E-07', '0.00E+00', '0.00E+00', '0.00E+00', '0.00E+00', '0.00E+00', '0.00E+00'] | 12.2 | Upregulated |
| 9 | Q7RTV0\|PHF5A_HUMAN | PHF5A | 1.07E-01 | 2.59E-03 | 3.87E-02 |  | ['0.00E+00', '1.21E-07', '1.06E-06', '2.23E-07', '5.81E-07', '0.00E+00', '4.16E-07', '1.14E-07', '0.00E+00', '1.47E-07'] | ['7.26E-07', '8.38E-07', '7.76E-07', '1.47E-06', '1.04E-06', '1.34E-06', '2.97E-07', '1.35E-06', '1.03E-06', '1.30E-06'] | 0.3 | Downregulated |
| 10 | O75487\|GPC4_HUMAN | GPC4 | 1.21E-01 | 3.71E-03 | 4.62E-02 |  | ['6.45E-08', '4.99E-08', '8.75E-08', '1.96E-07', '2.08E-07', '1.16E-07', '9.13E-08', '8.07E-08', '2.00E-08', '0.00E+00'] | ['3.61E-07', '3.00E-07', '1.62E-07', '2.09E-07', '1.45E-07', '1.61E-07', '3.46E-07', '2.75E-07', '2.63E-07', '2.88E-07'] | 0.4 | Downregulated |
| 11 | Q15334\|L2GL1_HUMAN | LLGL1 | 1.39E-01 | 3.04E-03 | 4.24E-02 |  | ['3.19E-08', '2.49E-08', '2.99E-08', '2.96E-08', '0.00E+00', '0.00E+00', '1.31E-08', '3.33E-08', '0.00E+00', '1.59E-08'] | ['1.25E-08', '0.00E+00', '0.00E+00', '0.00E+00', '0.00E+00', '0.00E+00', '0.00E+00', '0.00E+00', '0.00E+00', '0.00E+00'] | 14.3 | Upregulated |
| 12 | Q15004\|PAF15_HUMAN | KIAA0101 | 1.40E-01 | 2.82E-03 | 4.06E-02 |  | ['1.49E-07', '1.29E-07', '2.76E-07', '1.68E-07', '4.20E-07', '4.23E-07', '4.05E-07', '0.00E+00', '1.26E-07', '0.00E+00'] | ['0.00E+00', '0.00E+00', '0.00E+00', '0.00E+00', '0.00E+00', '0.00E+00', '0.00E+00', '0.00E+00', '0.00E+00', '0.00E+00'] | NA in controls | Upregulated |
| 13 | P03923\|NU6M_HUMAN | MT-ND6 | 1.82E-01 | 3.26E-03 | 4.36E-02 |  | ['0.00E+00', '0.00E+00', '0.00E+00', '0.00E+00', '0.00E+00', '0.00E+00', '0.00E+00', '0.00E+00', '0.00E+00', '0.00E+00'] | ['1.53E-07', '1.54E-07', '2.59E-07', '1.41E-07', '8.35E-08', '0.00E+00', '1.30E-07', '0.00E+00', '1.68E-07', '0.00E+00'] | NA in IFN-treated | Downregulated |
| 14 | Q10589-2\|BST2_HUMAN | BST2 | 1.91E-01 | 3.08E-03 | 4.25E-02 |  | ['0.00E+00', '5.52E-07', '1.06E-06', '9.26E-07', '0.00E+00', '9.08E-07', '1.22E-06', '4.44E-07', '0.00E+00', '7.50E-07'] | ['0.00E+00', '0.00E+00', '0.00E+00', '0.00E+00', '0.00E+00', '0.00E+00', '0.00E+00', '0.00E+00', '0.00E+00', '0.00E+00'] | NA in controls | Upregulated |
| 15 | Q99829\|CPNE1_HUMAN | CPNE1 | 2.43E-01 | 3.49E-03 | 4.52E-02 |  | ['7.19E-08', '5.19E-08', '1.19E-07', '1.12E-07', '9.79E-08', '1.49E-07', '1.34E-07', '0.00E+00', '1.40E-07', '6.72E-08'] | ['8.57E-08', '0.00E+00', '0.00E+00', '4.66E-08', '0.00E+00', '4.06E-08', '0.00E+00', '3.38E-08', '0.00E+00', '0.00E+00'] | 4.6 | Upregulated |
| 16 | Q6PCE3\|PGM2L_HUMAN | PGM2L1 | 2.52E-01 | 2.79E-03 | 4.04E-02 |  | ['3.05E-08', '0.00E+00', '1.80E-08', '7.36E-08', '3.03E-08', '5.30E-08', '4.23E-08', '3.92E-08', '0.00E+00', '1.67E-08'] | ['0.00E+00', '0.00E+00', '0.00E+00', '1.84E-08', '0.00E+00', '0.00E+00', '0.00E+00', '0.00E+00', '0.00E+00', '1.38E-08'] | 9.4 | Upregulated |
| 17 | Q9Y3D9\|RT23_HUMAN | MRPS23 | 3.20E-01 | 4.03E-03 | 4.92E-02 |  | ['0.00E+00', '0.00E+00', '4.25E-07', '2.29E-07', '3.98E-07', '0.00E+00', '2.50E-07', '3.04E-07', '3.62E-07', '0.00E+00'] | ['6.55E-07', '7.52E-07', '1.98E-06', '3.02E-07', '3.03E-07', '7.14E-07', '7.53E-07', '7.81E-07', '5.95E-07', '6.17E-07'] | 0.3 | Downregulated |
| 18 | Q0P6H9\|TMM62_HUMAN | TMEM62 | 3.32E-01 | 2.51E-03 | 3.81E-02 |  | ['1.38E-08', '3.14E-08', '6.68E-08', '2.70E-08', '0.00E+00', '6.17E-08', '1.89E-08', '0.00E+00', '5.29E-08', '5.44E-08'] | ['0.00E+00', '0.00E+00', '0.00E+00', '0.00E+00', '0.00E+00', '0.00E+00', '0.00E+00', '0.00E+00', '0.00E+00', '0.00E+00'] | NA in controls | Upregulated |
| 19 | Q99439\|CNN2_HUMAN | CNN2 | 3.33E-01 | 2.42E-03 | 3.76E-02 |  | ['9.98E-07', '9.94E-07', '1.03E-06', '9.72E-07', '1.06E-06', '5.55E-07', '9.16E-07', '6.16E-07', '7.10E-07', '7.83E-07'] | ['3.68E-07', '3.69E-07', '2.38E-07', '0.00E+00', '0.00E+00', '8.74E-07', '6.35E-07', '0.00E+00', '0.00E+00', '6.69E-07'] | 2.7 | Upregulated |
| 20 | Q96B97\|SH3K1_HUMAN | SH3KBP1 | 3.80E-01 | 1.67E-03 | 2.99E-02 |  | ['9.16E-08', '2.57E-07', '2.38E-07', '2.93E-07', '1.57E-07', '2.03E-07', '3.88E-08', '9.74E-08', '2.88E-07', '1.22E-07'] | ['2.47E-08', '0.00E+00', '0.00E+00', '1.42E-07', '5.52E-08', '4.77E-08', '0.00E+00', '9.62E-08', '2.10E-08', '6.30E-08'] | 4 | Upregulated |
| 21 | Q9BXJ9\|NAA15_HUMAN | NAA15 | 3.94E-01 | 1.73E-03 | 3.02E-02 |  | ['1.32E-07', '5.50E-08', '3.46E-08', '9.99E-08', '8.41E-08', '8.82E-08', '6.75E-08', '1.05E-07', '3.56E-08', '7.91E-08'] | ['2.68E-08', '7.94E-08', '0.00E+00', '6.54E-08', '2.16E-08', '4.84E-08', '0.00E+00', '0.00E+00', '0.00E+00', '0.00E+00'] | 3.2 | Upregulated |
| 22 | Q9Y6A5\|TACC3_HUMAN | TACC3 | 3.98E-01 | 3.25E-03 | 4.36E-02 |  | ['0.00E+00', '1.00E-08', '4.46E-08', '1.75E-08', '1.40E-08', '3.08E-08', '1.78E-08', '2.03E-08', '1.32E-08', '0.00E+00'] | ['0.00E+00', '0.00E+00', '0.00E+00', '0.00E+00', '0.00E+00', '0.00E+00', '0.00E+00', '0.00E+00', '0.00E+00', '0.00E+00'] | NA in controls | Upregulated |
| 23 | O95456-2\|PSMG1_HUMAN | PSMG1 | 4.46E-01 | 1.68E-03 | 2.99E-02 |  | ['1.43E-07', '1.75E-07', '1.73E-07', '2.96E-07', '1.76E-07', '1.86E-07', '1.54E-07', '1.41E-07', '2.86E-07', '1.29E-07'] | ['7.57E-08', '0.00E+00', '0.00E+00', '0.00E+00', '9.67E-08', '1.70E-07', '0.00E+00', '0.00E+00', '0.00E+00', '1.21E-07'] | 4 | Upregulated |
| 24 | P11177-3\|ODPB_HUMAN | PDHB | 4.59E-01 | 3.10E-03 | 4.26E-02 |  | ['0.00E+00', '2.65E-07', '0.00E+00', '0.00E+00', '2.53E-07', '0.00E+00', '0.00E+00', '8.60E-08', '2.35E-07', '2.78E-07'] | ['6.78E-07', '2.27E-07', '1.54E-06', '1.43E-06', '9.47E-07', '3.16E-07', '4.80E-07', '9.23E-07', '3.23E-07', '7.77E-07'] | 0.1 | Downregulated |
| 25 | P32456\|GBP2_HUMAN | GBP2 | 4.66E-01 | 2.57E-03 | 3.85E-02 |  | ['9.16E-08', '3.80E-07', '4.45E-08', '2.58E-07', '2.14E-07', '2.21E-07', '1.82E-07', '2.49E-07', '0.00E+00', '0.00E+00'] | ['0.00E+00', '0.00E+00', '0.00E+00', '0.00E+00', '0.00E+00', '0.00E+00', '0.00E+00', '0.00E+00', '0.00E+00', '0.00E+00'] | NA in controls | Upregulated |
| 26 | P41567\|EIF1_HUMAN | EIF1 | 4.74E-01 | 1.79E-03 | 3.10E-02 |  | ['7.29E-07', '1.48E-06', '1.57E-06', '1.17E-06', '1.27E-06', '1.24E-06', '1.52E-06', '1.30E-06', '0.00E+00', '1.37E-06'] | ['0.00E+00', '8.85E-07', '3.59E-07', '7.35E-07', '0.00E+00', '0.00E+00', '0.00E+00', '8.68E-07', '2.64E-07', '9.75E-07'] | 2.9 | Upregulated |
| 27 | P31350-2\|RIR2_HUMAN | RRM2 | 5.07E-01 | 2.84E-03 | 4.07E-02 |  | ['8.05E-08', '8.97E-08', '2.23E-07', '7.70E-08', '8.08E-08', '1.95E-07', '9.70E-08', '2.04E-07', '1.10E-07', '1.04E-07'] | ['7.82E-08', '0.00E+00', '0.00E+00', '4.74E-08', '5.01E-08', '4.43E-08', '0.00E+00', '4.87E-08', '4.93E-08', '5.50E-08'] | 3.4 | Upregulated |
| 28 | Q9NX46\|ARHL2_HUMAN | ADPRHL2 | 5.11E-01 | 3.33E-03 | 4.42E-02 |  | ['9.96E-08', '0.00E+00', '1.07E-07', '2.57E-08', '8.71E-08', '1.07E-07', '8.58E-08', '1.58E-07', '0.00E+00', '6.13E-08'] | ['0.00E+00', '0.00E+00', '0.00E+00', '0.00E+00', '0.00E+00', '8.29E-08', '0.00E+00', '0.00E+00', '0.00E+00', '0.00E+00'] | 8.8 | Upregulated |
| 29 | Q03001\|DYST_HUMAN | DST | 5.23E-01 | 3.68E-03 | 4.61E-02 |  | ['0.00E+00', '2.37E-08', '0.00E+00', '1.78E-08', '2.21E-08', '0.00E+00', '0.00E+00', '0.00E+00', '0.00E+00', '1.95E-08'] | ['0.00E+00', '4.51E-08', '2.06E-08', '4.77E-08', '1.88E-08', '4.74E-08', '2.68E-08', '4.09E-08', '2.26E-08', '2.18E-08'] | 0.3 | Downregulated |
| 30 | Q96FW1\|OTUB1_HUMAN | OTUB1 | 5.80E-01 | 3.61E-03 | 4.57E-02 |  | ['0.00E+00', '2.56E-07', '6.72E-08', '6.71E-08', '2.38E-07', '0.00E+00', '4.17E-07', '1.50E-07', '2.97E-07', '2.69E-07'] | ['0.00E+00', '4.30E-08', '0.00E+00', '0.00E+00', '0.00E+00', '0.00E+00', '0.00E+00', '0.00E+00', '0.00E+00', '4.16E-08'] | 20.8 | Upregulated |
| 31 | P07203\|GPX1_HUMAN | GPX1 | 6.83E-01 | 1.92E-03 | 3.23E-02 |  | ['1.41E-07', '1.28E-07', '2.03E-07', '2.11E-07', '0.00E+00', '1.56E-07', '6.11E-08', '1.51E-07', '1.37E-07', '1.29E-07'] | ['0.00E+00', '0.00E+00', '0.00E+00', '1.12E-07', '0.00E+00', '1.29E-07', '0.00E+00', '1.05E-07', '0.00E+00', '9.44E-08'] | 3 | Upregulated |
| 32 | P30519\|HMOX2_HUMAN | HMOX2 | 6.85E-01 | 3.23E-03 | 4.36E-02 |  | ['0.00E+00', '2.27E-07', '3.06E-07', '0.00E+00', '0.00E+00', '2.29E-07', '2.18E-07', '0.00E+00', '0.00E+00', '2.99E-07'] | ['5.76E-07', '3.79E-07', '5.37E-07', '2.94E-07', '4.45E-07', '2.84E-07', '5.39E-07', '3.48E-07', '0.00E+00', '2.98E-07'] | 0.3 | Downregulated |
| 33 | Q9H223\|EHD4_HUMAN | EHD4 | 6.95E-01 | 1.72E-03 | 3.02E-02 |  | ['1.99E-07', '2.86E-07', '3.23E-07', '3.38E-07', '4.70E-07', '4.15E-07', '2.89E-07', '2.39E-07', '1.79E-07', '2.39E-07'] | ['7.11E-08', '7.72E-08', '6.63E-08', '1.29E-07', '6.74E-08', '5.57E-08', '1.84E-07', '1.22E-07', '2.36E-07', '1.12E-07'] | 2.7 | Upregulated |
| 34 | O14524\|NEMP1_HUMAN | NEMP1 | 7.02E-01 | 1.85E-03 | 3.16E-02 |  | ['0.00E+00', '0.00E+00', '3.49E-08', '0.00E+00', '9.52E-08', '3.84E-08', '0.00E+00', '0.00E+00', '0.00E+00', '0.00E+00'] | ['1.02E-07', '9.54E-08', '1.78E-07', '2.10E-07', '1.37E-07', '9.03E-08', '2.94E-07', '0.00E+00', '1.55E-07', '9.28E-08'] | 0.1 | Downregulated |
| 35 | O94808\|GFPT2_HUMAN | GFPT2 | 7.13E-01 | 2.88E-03 | 4.10E-02 |  | ['1.11E-07', '1.37E-07', '9.93E-08', '1.16E-07', '4.12E-08', '7.97E-08', '1.34E-07', '1.07E-07', '1.17E-07', '1.14E-07'] | ['0.00E+00', '4.84E-08', '1.29E-08', '4.77E-08', '6.63E-08', '5.86E-08', '0.00E+00', '5.24E-08', '0.00E+00', '1.07E-07'] | 2.7 | Upregulated |
| 36 | O15355\|PPM1G_HUMAN | PPM1G | 7.23E-01 | 2.50E-03 | 3.81E-02 |  | ['2.31E-07', '1.36E-07', '3.57E-07', '2.81E-07', '1.66E-07', '3.96E-07', '1.61E-07', '1.73E-07', '1.08E-07', '1.38E-07'] | ['0.00E+00', '2.51E-08', '1.89E-08', '7.84E-08', '1.06E-07', '1.59E-08', '0.00E+00', '8.51E-08', '1.44E-08', '1.59E-07'] | 4.3 | Upregulated |
| 37 | Q07020\|RL18_HUMAN | RPL18 | 7.66E-01 | 2.77E-03 | 4.04E-02 |  | ['6.89E-06', '7.65E-06', '6.69E-06', '8.13E-06', '4.85E-06', '4.51E-06', '7.17E-06', '6.13E-06', '3.55E-06', '3.64E-06'] | ['0.00E+00', '2.95E-06', '6.19E-06', '0.00E+00', '0.00E+00', '0.00E+00', '5.30E-06', '0.00E+00', '5.49E-06', '0.00E+00'] | 3 | Upregulated |
| 38 | Q06124\|PTN11_HUMAN | PTPN11 | 7.94E-01 | 3.20E-03 | 4.36E-02 |  | ['1.71E-08', '1.69E-08', '3.37E-08', '6.40E-08', '4.70E-08', '3.45E-08', '4.82E-08', '1.91E-08', '2.99E-08', '1.59E-08'] | ['2.94E-08', '0.00E+00', '0.00E+00', '1.21E-08', '2.43E-08', '2.48E-08', '0.00E+00', '0.00E+00', '0.00E+00', '0.00E+00'] | 3.6 | Upregulated |
| 39 | P49915-2\|GUAA_HUMAN | GMPS | 8.81E-01 | 3.46E-03 | 4.51E-02 |  | ['1.70E-07', '7.88E-08', '2.40E-07', '1.86E-07', '7.94E-08', '8.95E-08', '1.31E-07', '1.12E-07', '1.67E-07', '1.79E-07'] | ['0.00E+00', '1.75E-08', '0.00E+00', '9.18E-08', '6.50E-08', '9.86E-08', '0.00E+00', '8.59E-08', '7.78E-08', '4.45E-08'] | 3 | Upregulated |
| 40 | Q8WUM4-2\|PDC6I_HUMAN | PDCD6IP | 9.87E-01 | 3.25E-03 | 4.36E-02 |  | ['1.06E-07', '1.16E-07', '1.03E-07', '1.29E-07', '9.17E-08', '1.62E-07', '5.07E-08', '5.24E-08', '1.47E-07', '8.08E-08'] | ['3.88E-08', '3.05E-08', '5.25E-08', '1.25E-08', '0.00E+00', '0.00E+00', '8.90E-08', '3.56E-08', '2.00E-08', '3.44E-08'] | 3.3 | Upregulated |
