# Supplementary material for: Comparative proteomics as a tool for identifying specific alterations within interferon response pathways in human glioblastoma multiforme cells
Source: Oncotarget. 2017 Nov 29;9(2):1785–802. doi: 10.18632/oncotarget.22751 (PMC5788599; doi:10.18632/oncotarget.22751)
Supplement: Supplementary file 3 [file oncotarget-09-1785-s003.docx]

Supplementary Table 2. Enriched biological processes identified in glioblastoma lines. Target list: differentially expressed proteins derived using *workflow A*. Top 20 biological processes are shown.

(A)

| **A-172** | | | | | | | | | | | |
| --- | --- | --- | --- | --- | --- | --- | --- | --- | --- | --- | --- |
| **INTERFEROME** | | | | **GOrilla** | | | | **STRING** | | | |
| Accession | Process | Enriched  IRGs | p Value | Accession | Process | Enriched  genes | p Value | Accession | Process | Enriched genes | p Value |
| GO:0019221 | cytokine-mediated signaling pathway | 17 | < 1E-015 | GO:0060337 | type I IFN signaling pathway | 11 | 5.29E-17 | GO.0051607 | defense response to virus | 14 | 2.95E-015 |
| GO:0060337 | type I IFN-mediated signaling pathway | 15 | < 1E-015 | GO:0051607 | defense response to virus | 14 | 7.11E-17 | GO.0060337 | type I IFN signaling pathway | 11 | 1.07E-014 |
| GO:0044419 | interspecies interaction between organisms | 12 | < 1E-015 | GO:0060333 | IFN-gamma-mediated signaling pathway | 10 | 3.29E-15 | GO.0071357 | cellular response to type I IFN | 11 | 1.07E-014 |
| GO:0009615 | response to virus | 12 | < 1E-015 | GO:0006952 | defense response | 19 | 4.55E-15 | GO.0019221 | cytokine-mediated signaling pathway | 16 | 1.34E-013 |
| GO:0060333 | IFN-gamma-mediated signaling pathway | 11 | < 1E-015 | GO:0019221 | cytokine-mediated signaling pathway | 17 | 9.57E-15 | GO.0006952 | defense response | 23 | 1.41E-012 |
| GO:0002474 | antigen processing and presentation of peptide antigen via MHC class I | 10 | < 1E-015 | GO:0098542 | defense response to other organism | 14 | 4.10E-14 | GO.0045087 | innate immune response | 20 | 1.5E-012 |
| GO:0002479 | antigen processing and presentation of exogenous peptide antigen via MHC class I;TAP-dependent | 8 | < 1E-015 | GO:0009615 | response to virus | 14 | 5.29E-14 | GO.0048525 | negative regulation of viral process | 10 | 2.89E-012 |
| GO:0042590 | antigen processing and presentation of exogenous peptide antigen via MHC class I | 8 | < 1E-015 | GO:0048525 | negative regulation of viral process | 10 | 7.28E-13 | GO.0002252 | immune effector process | 15 | 1.27E-011 |
| GO:0006955 | immune response | 11 | 1E-015 | GO:0051707 | response to other organism | 15 | 1.20E-12 | GO.0060333 | IFN-gamma-mediated signaling pathway | 9 | 1.96E-011 |
| GO:0002480 | antigen processing and presentation of exogenous peptide antigen via MHC class I;TAP-independent | 5 | 1.27E-012 | GO:1903901 | negative regulation of viral life cycle | 9 | 6.14E-12 | GO.1903901 | negative regulation of viral life cycle | 9 | 1.07E-010 |
| GO:0019882 | antigen processing and presentation | 6 | 1.83E-011 | GO:0043207 | response to external biotic stimulus | 16 | 9.92E-12 | GO.0034097 | response to cytokine | 16 | 1.15E-010 |
| GO:0019885 | antigen processing and presentation of endogenous peptide antigen via MHC class I | 4 | 1.88E-010 | GO:0009607 | response to biotic stimulus | 16 | 1.86E-11 | GO.0051707 | response to other organism | 16 | 2.69E-010 |
| GO:0050776 | regulation of immune response | 5 | 2.47E-009 | GO:0043901 | negative regulation of multi-organism process | 10 | 4.28E-11 | GO.0071346 | cellular response to IFN-gamma | 9 | 3.21E-010 |
| GO:0006952 | defense response | 4 | 8.52E-007 | GO:0045071 | negative regulation of viral genome replication | 7 | 1.10E-09 | GO.0071345 | cellular response to cytokine stimulus | 14 | 1.92E-009 |
| GO:0050823 | peptide antigen stabilization | 2 | 1.79E-006 | GO:0051704 | multi-organism process | 21 | 2.46E-09 | GO.0050792 | regulation of viral process | 9 | 1.00E-007 |
| GO:0019060 | intracellular transport of viral proteins in host cell | 2 | 2.03E-005 | GO:1903900 | regulation of viral life cycle | 9 | 4.33E-09 | GO.0051704 | multi-organism process | 21 | 3.44E-007 |
| GO:0034097 | response to cytokine stimulus | 3 | 2.04E-005 | GO:0050792 | regulation of viral process | 10 | 1.36E-08 | GO.0002376 | immune system process | 20 | 3.57E-007 |
| GO:0006461 | protein complex assembly | 3 | 3.23E-005 | GO:0002483 | antigen processing and presentation of endogenous peptide antigen | 4 | 1.58E-08 | GO.0045071 | negative regulation of viral genome replication | 6 | 3.57E-007 |
| GO:0048384 | retinoic acid receptor signaling pathway | 2 | 5.51E-005 | GO:0019883 | antigen processing and presentation of endogenous antigen | 4 | 1.58E-08 | GO.0006950 | response to stress | 25 | 6.36E-007 |
| GO:0032480 | negative regulation of type I IFN production | 2 | 6.64E-005 | GO:0019885 | antigen processing and presentation of endogenous peptide antigen via MHC class I | 4 | 1.58E-08 | GO.1903900 | regulation of viral life cycle | 8 | 1.42E-006 |

(B)

| **DBTRG-05MG** | | | | | | | | | | | |
| --- | --- | --- | --- | --- | --- | --- | --- | --- | --- | --- | --- |
| **INTERFEROME** | | | | **GOrilla** | | | | **STRING** | | | |
| Accession | Process | Enriched  IRGs | p Value | Accession | Process | Enriched  genes | p Value | Accession | Process | Enriched genes | FDR_STRING_ |
| GO:0009615 | response to virus | 14 | < 1E-015 | GO:0060337 | type I IFN signaling pathway | 14 | 3.65E-17 | GO.0051607 | defense response to virus | 17 | 1.48E-014 |
| GO:0019221 | cytokine-mediated signaling pathway | 13 | < 1E-015 | GO:0051607 | defense response to virus | 17 | 4.47E-15 | GO.0060337 | type I IFN signaling pathway | 13 | 3.27E-014 |
| GO:0060337 | type I IFN-mediated signaling pathway | 12 | < 1E-015 | GO:0009615 | response to virus | 19 | 7.63E-14 | GO.0071357 | cellular response to type I IFN | 13 | 3.27E-014 |
| GO:0060333 | IFN-gamma-mediated signaling pathway | 6 | 3.11E-011 | GO:0006952 | defense response | 25 | 3.39E-13 | GO.0019221 | cytokine-mediated signaling pathway | 19 | 3.05E-011 |
| GO:0044419 | interspecies interaction between organisms | 7 | 4.49E-011 | GO:0098542 | defense response to other organism | 18 | 5.86E-13 | GO.0071345 | cellular response to cytokine stimulus | 19 | 5.82E-009 |
| GO:0032480 | negative regulation of type I IFN production | 3 | 2.34E-007 | GO:0060333 | IFN-gamma-mediated signaling pathway | 11 | 4.43E-12 | GO.0048525 | negative regulation of viral process | 10 | 9.08E-009 |
| GO:0045071 | negative regulation of viral genome replication | 3 | 3.30E-007 | GO:0051707 | response to other organism | 20 | 2.11E-11 | GO.0034097 | response to cytokine | 20 | 1.13E-008 |
| GO:0007259 | JAK-STAT cascade | 3 | 1.14E-006 | GO:0019221 | cytokine-mediated signaling pathway | 20 | 3.76E-11 | GO.0060333 | IFN-gamma-mediated signaling pathway | 9 | 2.64E-008 |
| GO:0035457 | cellular response to IFN-alpha | 2 | 9.29E-006 | GO:0043207 | response to external biotic stimulus | 21 | 3.16E-10 | GO.0045087 | innate immune response | 23 | 2.75E-008 |
| GO:0019060 | intracellular transport of viral proteins in host cell | 2 | 2.03E-005 | GO:0009607 | response to biotic stimulus | 21 | 7.05E-10 | GO.0043901 | negative regulation of multi-organism process | 11 | 8.88E-008 |
| GO:0045087 | innate immune response | 4 | 2.33E-005 | GO:0002376 | immune system process | 35 | 4.49E-08 | GO.1903901 | negative regulation of viral life cycle | 9 | 1.21E-007 |
| GO:0006401 | RNA catabolic process | 2 | 4.48E-005 | GO:0002252 | immune effector process | 25 | 8.74E-08 | GO.0071346 | cellular response to IFN-gamma | 9 | 4.32E-007 |
| GO:0060338 | regulation of type I IFN-mediated signaling pathway | 2 | 4.81E-005 | GO:0048525 | negative regulation of viral process | 9 | 1.36E-07 | GO.0051707 | Response to other organism | 18 | 1.2E-006 |
| GO:0043330 | response to exogenous dsRNA | 2 | 5.87E-005 | GO:0034097 | response to cytokine | 14 | 9.97E-07 | GO.0006952 | defense response | 25 | 1.66E-006 |
| GO:0032091 | negative regulation of protein binding | 2 | 1.17E-004 | GO:0009605 | response to external stimulus | 22 | 1.86E-06 | GO.0045069 | regulation of viral genome replication | 7 | 2.37E-005 |
| GO:0006955 | immune response | 4 | 1.27E-004 | GO:0043901 | negative regulation of multi-organism process | 9 | 3.79E-06 | GO.0045071 | negative regulation of viral genome replication | 6 | 4.34E-005 |
| GO:0030683 | evasion or tolerance by virus of host immune response | 1 | 3.51E-004 | GO:0034340 | response to type I IFN | 4 | 4.94E-06 | GO.0070887 | cellular response to chemical stimulus | 29 | 8.16E-005 |
| GO:0006139 | nucleobase-containing compound metabolic process | 2 | 4.86E-004 | GO:1903901 | negative regulation of viral life cycle | 7 | 7.62E-06 | GO.0050792 | regulation of viral process | 9 | 9.25E-005 |
| GO:0034344 | regulation of type III IFN production | 1 | 7.01E-004 | GO:0045087 | innate immune response | 10 | 9.46E-06 | GO.0006950 | response to stress | 36 | 1.33E-004 |
| GO:0039528 | cytoplasmic pattern recognition receptor signaling pathway in response to virus | 1 | 7.01E-004 | GO:1903900 | regulation of viral life cycle | 9 | 1.03E-05 | GO.0002376 | immune system process | 26 | 2.16E-004 |
