# Supplementary material for: Comparative proteomics as a tool for identifying specific alterations within interferon response pathways in human glioblastoma multiforme cells
Source: Oncotarget. 2017 Nov 29;9(2):1785–802. doi: 10.18632/oncotarget.22751 (PMC5788599; doi:10.18632/oncotarget.22751)
Supplement: Supplementary file 4 [file oncotarget-09-1785-s004.docx]

**Supplementary Table 3.** Results of GO analyses for A-172 and DBTRG-05MG lines evaluated using the combined statistical analysis *paired t-test (ND) / Kruskal-Wallis test (NND)* after missing value imputation (Workflow C). Top 20 processes are shown.

| **A-172** | | | | | | | | | | | |
| --- | --- | --- | --- | --- | --- | --- | --- | --- | --- | --- | --- |
| **INTERFEROME** | | | | **GOrilla** | | | | **STRING** | | | |
| Accession | Process | Enriched  IRGs | p Value | Accession | Process | Enriched  genes | p Value | Accession | Process | Enriched genes | p Value |
| GO:0019221 | cytokine-mediated signaling pathway | 24 | < 1.00E-12 | GO:0060337 | type I interferon signaling pathway | 18 | 1.05E-12 | GO.0016043 | cellular component organization | 178 | 6.06E-09 |
| GO:0060337 | type I interferon-mediated signaling pathway | 21 | < 1.00E-12 | GO:0060333 | interferon-gamma-mediated signaling pathway | 14 | 1.78E-08 | GO.0071840 | cellular component organization or biogenesis | 182 | 6.06E-09 |
| GO:0044419 | interspecies interaction between organisms | 16 | < 1.00E-12 | GO:0051607 | defense response to virus | 23 | 2.12E-08 | GO.0051607 | defense response to virus | 23 | 3.28E-08 |
| GO:0009615 | response to virus | 16 | < 1.00E-12 | GO:0006952 | defense response | 48 | 7.80E-08 | GO.1902589 | single-organism organelle organization | 94 | 5.62E-08 |
| GO:0060333 | interferon-gamma-mediated signaling pathway | 15 | < 1.00E-12 | GO:0098542 | defense response to other organism | 28 | 1.91E-07 | GO.0006996 | organelle organization | 119 | 2.08E-07 |
| GO:0006955 | immune response | 14 | < 1.00E-12 | GO:0009615 | response to virus | 26 | 3.46E-06 | GO.0060337 | type I interferon signaling pathway | 15 | 2.08E-07 |
| GO:0002474 | antigen processing and presentation of peptide antigen via MHC class I | 14 | < 1.00E-12 | GO:0002483 | antigen processing and presentation of endogenous peptide antigen | 5 | 5.19E-06 | GO.0071357 | cellular response to type I interferon | 15 | 2.08E-07 |
| GO:0002479 | antigen processing and presentation of exogenous peptide antigen via MHC class I;TAP-dependent | 12 | < 1.00E-12 | GO:0019883 | antigen processing and presentation of endogenous antigen | 5 | 5.19E-06 | GO.0009615 | response to virus | 25 | 8.77E-07 |
| GO:0042590 | antigen processing and presentation of exogenous peptide antigen via MHC class I | 12 | < 1.00E-12 | GO:0019885 | antigen processing and presentation of endogenous peptide antigen via MHC class I | 5 | 5.19E-06 | GO.0008152 | metabolic process | 283 | 1.88E-06 |
| GO:0019882 | antigen processing and presentation | 9 | < 1.00E-12 | GO:0051707 | response to other organism | 32 | 3.52E-05 | GO.0043933 | macromolecular complex subunit organization | 93 | 1.88E-06 |
| GO:0002480 | antigen processing and presentation of exogenous peptide antigen via MHC class I;TAP-independent | 7 | < 1.00E-12 | GO:0019221 | cytokine-mediated signaling pathway | 33 | 5.69E-05 | GO.0006950 | response to stress | 129 | 8.27E-06 |
| GO:0019885 | antigen processing and presentation of endogenous peptide antigen via MHC class I | 5 | 2.27E-12 | GO:0048525 | negative regulation of viral process | 13 | 8.57E-05 | GO.0048525 | negative regulation of viral process | 14 | 1.36E-05 |
| GO:0050776 | regulation of immune response | 7 | 2.50E-12 | GO:0043207 | response to external biotic stimulus | 38 | 2.05E-04 | GO.0019222 | regulation of metabolic process | 201 | 3.66E-05 |
| GO:0019060 | intracellular transport of viral proteins in host cell | 3 | 1.36E-07 | GO:0009605 | response to external stimulus | 58 | 2.92E-04 | GO.0050792 | regulation of viral process | 18 | 4.20E-04 |
| GO:0032480 | negative regulation of type I interferon production | 3 | 8.34E-07 | GO:1903901 | negative regulation of viral life cycle | 11 | 2.94E-04 | GO.0060333 | interferon-gamma-mediated signaling pathway | 11 | 4.20E-04 |
| GO:0045071 | negative regulation of viral genome replication | 3 | 1.17E-06 | GO:0044236 | multicellular organism metabolic process | 11 | 3.78E-04 | GO.0071822 | protein complex subunit organization | 64 | 4.20E-04 |
| GO:0007259 | JAK-STAT cascade | 3 | 4.03E-06 | GO:0045071 | negative regulation of viral genome replication | 9 | 4.45E-04 | GO.1903901 | negative regulation of viral life cycle | 12 | 4.20E-04 |
| GO:0050823 | peptide antigen stabilization | 2 | 4.15E-06 | GO:0009607 | response to biotic stimulus | 38 | 4.78E-04 | GO.0098542 | defense response to other organism | 26 | 4.56E-04 |
| GO:0006952 | defense response | 4 | 4.59E-06 | GO:0006955 | immune response | 31 | 6.00E-04 | GO.0045069 | regulation of viral genome replication | 11 | 7.19E-04 |
| GO:0032020 | ISG15-protein conjugation | 2 | 1.82E-05 | GO:0006954 | inflammatory response | 16 | 6.55E-04 | GO.0009987 | cellular process | 330 | 9.01E-04 |

(B)

| **DBTRG-05MG** | | | | | | | | | | | |
| --- | --- | --- | --- | --- | --- | --- | --- | --- | --- | --- | --- |
| **INTERFEROME** | | | | **GOrilla** | | | | **STRING** | | | |
| Accession | Process | Enriched  IRGs | p Value | Accession | Process | Enriched  genes | p Value | Accession | Process | Enriched genes | p Value |
| GO:0009615 | response to virus | 20 | 0.00E+00 | GO:0051607 | defense response to virus | 33 | 3.82E-12 | GO.0007049 | cell cycle | 106 | 3.40E-14 |
| GO:0019221 | cytokine-mediated signaling pathway | 17 | 0.00E+00 | GO:0060337 | type I interferon signaling pathway | 20 | 1.77E-11 | GO.0051607 | defense response to virus | 33 | 1.35E-13 |
| GO:0060337 | type I interferon-mediated signaling pathway | 14 | 0.00E+00 | GO:0098542 | defense response to other organism | 39 | 6.42E-10 | GO.0022402 | cell cycle process | 89 | 1.41E-13 |
| GO:0060333 | interferon-gamma-mediated signaling pathway | 8 | 7.90E-14 | GO:0009615 | response to virus | 37 | 2.39E-08 | GO.0006996 | organelle organization | 163 | 6.72E-11 |
| GO:0045071 | negative regulation of viral genome replication | 5 | 2.51E-11 | GO:0060333 | interferon-gamma-mediated signaling pathway | 17 | 4.84E-08 | GO.0000278 | mitotic cell cycle | 72 | 7.61E-11 |
| GO:0044419 | interspecies interaction between organisms | 8 | 3.61E-11 | GO:0006952 | defense response | 58 | 4.06E-07 | GO.1902589 | single-organism organelle organization | 125 | 8.45E-11 |
| GO:0051607 | defense response to virus | 6 | 1.01E-10 | GO:0032479 | regulation of type I interferon production | 21 | 3.16E-06 | GO.0009615 | response to virus | 35 | 1.19E-10 |
| GO:0032480 | negative regulation of type I interferon production | 4 | 4.37E-09 | GO:0051707 | response to other organism | 44 | 3.68E-06 | GO.1903047 | mitotic cell cycle process | 65 | 6.62E-10 |
| GO:0035457 | cellular response to interferon-alpha | 3 | 5.03E-08 | GO:0019221 | cytokine-mediated signaling pathway | 43 | 2.08E-05 | GO.0034340 | response to type I interferon | 19 | 1.35E-09 |
| GO:0006955 | immune response | 7 | 2.26E-07 | GO:0009607 | response to biotic stimulus | 50 | 1.18E-04 | GO.0044237 | cellular metabolic process | 348 | 2.63E-09 |
| GO:0045087 | innate immune response | 6 | 3.30E-07 | GO:0018105 | peptidyl-serine phosphorylation | 17 | 1.46E-04 | GO.0008152 | metabolic process | 383 | 5.64E-09 |
| GO:0060700 | regulation of ribonuclease activity | 2 | 9.62E-07 | GO:0043207 | response to external biotic stimulus | 48 | 1.53E-04 | GO.0071840 | cellular component organization or biogenesis | 229 | 5.67E-09 |
| GO:0034340 | response to type I interferon | 2 | 9.62E-07 | GO:0048525 | negative regulation of viral process | 16 | 1.60E-04 | GO.0044699 | single-organism process | 413 | 6.01E-09 |
| GO:0007259 | JAK-STAT cascade | 3 | 5.02E-06 | GO:0000731 | DNA synthesis involved in DNA repair | 13 | 1.82E-04 | GO.0044763 | single-organism cellular process | 398 | 6.01E-09 |
| GO:0032020 | ISG15-protein conjugation | 2 | 2.11E-05 | GO:0009888 | tissue development | 33 | 2.36E-04 | GO.0016043 | cellular component organization | 224 | 6.14E-09 |
| GO:0050688 | regulation of defense response to virus | 2 | 2.49E-05 | GO:0030517 | negative regulation of axon extension | 5 | 2.77E-04 | GO.0045087 | innate immune response | 72 | 6.14E-09 |
| GO:0070206 | protein trimerization | 2 | 4.33E-05 | GO:1903901 | negative regulation of viral life cycle | 14 | 3.49E-04 | GO.0060337 | type I interferon signaling pathway | 18 | 6.14E-09 |
| GO:0019060 | intracellular transport of viral proteins in host cell | 2 | 5.43E-05 | GO:0000920 | cell separation after cytokinesis | 6 | 4.16E-04 | GO.0071357 | cellular response to type I interferon | 18 | 6.14E-09 |
| GO:0002479 | antigen processing and presentation of exogenous peptide antigen via MHC class I;TAP-dependent | 3 | 6.60E-05 | GO:0032647 | regulation of interferon-alpha production | 7 | 4.18E-04 | GO.0044238 | primary metabolic process | 346 | 3.35E-08 |
| GO:0042590 | antigen processing and presentation of exogenous peptide antigen via MHC class I | 3 | 7.66E-05 | GO:0012501 | programmed cell death | 51 | 4.90E-04 | GO.0009057 | macromolecule catabolic process | 69 | 3.66E-08 |
