# Supplementary material for: Comparative proteomics as a tool for identifying specific alterations within interferon response pathways in human glioblastoma multiforme cells
Source: Oncotarget. 2017 Nov 29;9(2):1785–802. doi: 10.18632/oncotarget.22751 (PMC5788599; doi:10.18632/oncotarget.22751)
Supplement: Supplementary file 5 [file oncotarget-09-1785-s005.docx]

**Supplementary Table 4.** Major protein components of the JAK/STAT cascade identified in glioblastoma proteomes. Protein names were matched against the following keys: "IFN", "INAR", "INGR", "JAK", "TYK", "STAT", "IRF ". Green highlights statistically significant change of protein abundance. Yellow highlights proteins of interest with abundance changes below the threshold of statistical significance. Protein q-value of 1.0 means the protein was absent in a sample. Samples are labeled as “Sb-r”, where S stands for the control (K) or IFN treated (IFN) samples, b is the biological repetition No., and r is the LC-MS/MS technical replicate No.
